# Trends in hypertension prevalence, awareness, treatment, and control in South Korea, 1998–2021: a nationally representative serial study

**DOI:** 10.1038/s41598-023-49055-8

**Published:** 2023-12-08

**Authors:** Myeongcheol Lee, Hojae Lee, Jaeyu Park, Hyeon Jin Kim, Rosie Kwon, Seung Won Lee, Sunyoung Kim, Ai Koyanagi, Lee Smith, Min Seo Kim, Guillaume Fond, Laurent Boyer, Masoud Rahmati, Sang Youl Rhee, Dong Keon Yon

**Affiliations:** 1 Center for Digital Health, Medical Science Research Institute, Kyung Hee University College of Medicine, Seoul, South Korea; 2 Department of Regulatory Science, Kyung Hee University, Seoul, South Korea; 3 Department of Precision Medicine, Sungkyunkwan University School of Medicine, Suwon, South Korea; 4 Department of Family Medicine, Kyung Hee University Medical Center, Kyung Hee University College of Medicine, Seoul, South Korea; 5 Research and Development Unit, Parc Sanitari Sant Joan de Deu, Barcelona, Spain; 6 Centre for Health, Performance and Wellbeing, Anglia Ruskin University, Cambridge, UK; 7 Medical and Population Genetics and Cardiovascular Disease Initiative, Broad Institute of MIT and Harvard, Cambridge, MA USA; 8 Assistance Publique-Hôpitaux de Marseille, Research Centre on Health Services and Quality of Life, Aix Marseille University, Marseille, France; 9 Department of Physical Education and Sport Sciences, Faculty of Literature and Humanities, Vali-E-Asr University of Rafsanjan, Rafsanjan, Iran; 10 Department of Physical Education and Sport Sciences, Faculty of Literature and Human Sciences, Lorestan University, Khoramabad, Iran; 11 Department of Endocrinology and Metabolism, Kyung Hee University College of Medicine, Kyung Hee University School of Medicine, 23 Kyungheedae-ro, Dongdaemun-gu, Seoul, 02447 South Korea; 12 Department of Pediatrics, Kyung Hee University Medical Center, Kyung Hee University College of Medicine, 23 Kyungheedae-ro, Dongdaemun-gu, Seoul, 02447 South Korea

**Keywords:** Cardiology, Health care, Medical research

## Abstract

The impact of the pandemic on hypertension management is unknown, particularly regarding changes in demographic risk factors. We conducted a comprehensive study between 1998 and 2021 on the long-term trends in hypertension prevalence in South Korea, including a comparison of the pre-pandemic and pandemic eras. Data from 1998 to 2021 of 108,687 Korean adults were obtained through a nationwide, large-scale, and serial study. We conducted a weighted complex sampling analysis on the estimates of national prevalence and compared the slope of hypertension prevalence before and during the pandemic to determine the trend dynamics. We included 108,687 participants over 24 years, 1998–2021. While the prevalence of patients with hypertension consistently increased before the pandemic from 25.51% [95% CI: 24.27–26.75] in 1998–2005 to 27.81% [95% CI: 26.97–28.66] in 2016–2019, the increasing slope in hypertension prevalence slowed during the pandemic period (28.07% [95% CI: 26.16–29.98] for 2021; β_diff_, −0.012 [−0.023 to 0.000]). Hypertension awareness, treatment, control, and control rates among patients receiving treatment followed similar trends. Compared to the pre-pandemic era, individuals aged 19–59 years or male had significantly increased control rates among the treated patients during the pandemic. This study investigated long-term trends in hypertension prevalence, awareness, treatment, and control among Korean adults. The absence of a reduction in the health indicators associated with hypertension during the pandemic implies that medical services for individuals with hypertension remain unaffected.

## Introduction

Hypertension is a leading public health concern worldwide and a major risk factor for cardiovascular diseases^1^. In South Korea, hypertension prevalence has been increasing steadily for several decades resulting in a significant burden on healthcare resources^2^. Thus, a thorough understanding of trends in hypertension prevalence, awareness, treatment, and control is crucial for developing effective prevention and control strategies^3^.

Furthermore, the COVID-19 pandemic has caused unprecedented disruptions to healthcare systems worldwide^4–6^. Consequently, the impact of the pandemic on hypertension management remains unclear, particularly regarding changes in demographic risk factors^7^. By comparing hypertension trends before and during the pandemic, it may be possible to ascertain the effect of the pandemic on hypertension management.

Thus, we aimed to present a comprehensive overview of hypertension trends in South Korea between 1998 and 2021, including a pre-pandemic and pandemic era comparison^8,9^. Additionally, we investigated the socioeconomic factors for hypertension in South Korea and these risk factors variations over time. Our findings will help policymakers and healthcare providers develop a focused treatment plan for hypertension management.

## Method

### Study population

The Korea Disease Control and Prevention Agency (KDCA) conducted the Korea National Health and Nutrition Examination Survey (KNHANES), a nationwide health information survey, using a stratified multistage probability sampling design to obtain nationally representative estimates of the Korean population^10,11^. Sample weights were accounted for by using primary sample units, households, and persons to ensure accurate representation. Each year, health interviews and physical examinations were conducted on a representative sample of the entire Korean civilian population^10,11^. Herein we utilized the KNHANES data from 1998 to 2021 to examine the trends in hypertension prevalence, awareness, treatment, control, and control among patients receiving treatment over 24 years. The study protocol was approved by the Institutional Review Board of Kyung Hee University (KHUH 2022-06-042) and the KDCA. All participants provided written informed consent. The research was carried out in accordance with the principles outlined in the Declaration of Helsinki.

We used 108,824 adults who completed hypertension evaluation; however, 137 were excluded owing to missing weight values. Thus, the final sample for data analysis included 108,687 participants at baseline, 47,062 (43.3%) men and 61,625 (56.7%) women.

### Time trend

We aimed to categorize the survey periods based on the investigation phases of KNHANES^10^. To stabilize prevalence, we grouped the first to third phases (1998, 2001, and 2005). Additionally, for the year 2019 corresponding to the 8th phase, we integrated it with the 7th phase and separately analyzed the pandemic period. Finally, to analyze changes during the pandemic period more sensitively, we opted to dissect and analyze the years 2020 and 2021. These years segments were: 1998–2005, 2007–2009, 2010–2012, 2013–2015, 2016–2019, 2020, and 2021. Because the first COVID-19 cases in South Korea were reported on January 20, 2020, we classified 2020 as the early pandemic era and 2021 as the mid-pandemic era^5,12,13^.

### Main outcome

The independent variables of this study were hypertension prevalence, awareness, treatment, control, and control among patients receiving treatment^3^. Participants were asked to self-report whether they had been previously diagnosed with hypertension. Subjects rested for over 5 min by sitting, and then the researcher allowed the subjects to properly place the cuff on their arm circumference. Blood pressure was measured using a Baumanometer Wall Unit 33 (mercury sphygmomanometer) from 1998 to 2019, Greenlight 300 (mercury-free auscultatory device) in 2020, and Microlife WatchBP Office AFIB (mercury-free automated blood pressure device) in 2021^14^. The researcher checked the participant's blood pressure two to three times on their right arm. We used the second value or the average of the second and third value of blood pressure for the study. Hypertension was classified as a systolic blood pressure of ≥ 140 mm Hg or a diastolic blood pressure of ≥ 90 mm Hg or being on medication for hypertension^3^. Awareness was determined by the percentage of participants with hypertension who answered "Yes" to the question, "Have you ever been told by a doctor or other healthcare professional that you had high blood pressure?"^3^. Although the guideline of the KNHANES analysis suggested that treatment is defined as the percentage of participants with hypertension who reported receiving more than 20 days of prescribed antihypertensive medication for its management, there was no detailed question about frequency between 1998 and 2005. To ensure consistent analysis, we have defined treatment as follows. Treatment was calculated as the percentage of participants with hypertension who reported receiving at least one prescribed antihypertensive drug for the management of hypertension^3^. Control was calculated as the percentage of participants with hypertension who had a systolic blood pressure < 140 mm Hg and a diastolic blood pressure < 90 mm Hg during the survey measurements. Control among those receiving treatment refers to individuals who are taking medication for hypertension and have a systolic blood pressure < 140 mm Hg and a diastolic blood pressure < 90 mm Hg as determined through measurements obtained during the survey.

### Covariates

The variables included in the analysis were sex, age (19–29, 30–39, 40–49, 50–59, 60–69, 70–79, and ≥ 80 years), region of residence (urban and rural)^15^, body mass index group (normal or underweight [< 23.0 kg/m^2^], overweight [23.0–25.0 kg/m^2^], obese [≥ 25.0 kg/m^2^], and unknown), central obesity (unknown, yes, and no), household income (unknown, lowest, second, third, and highest quartile), education background (elementary school or lower, middle school, high school, and college or higher), alcohol consumption (1–3 and ≥ 4 times a week and non-drinker), and smoking status (non-smoker, ex-smoker, and smoker). Central obesity was defined as having a waist circumference of ≥ 90 cm for men and ≥ 85 cm for women^3^.

### Statistical analysis

We conducted a weighted complex sampling analysis to examine the estimates of national hypertension prevalence, awareness, treatment, and control among those receiving treatment. We used the weights to calculate the crude rate. In addition, the age-standardized prevalence rates for hypertension were estimated. We utilized weighted linear regression models to examine the trend of hypertension prevalence, awareness, treatment, control, and control among patients receiving treatment over the last 24 years, focusing on the period during the COVID-19 outbreak^16^. A difference of β (β_diff_) and 95% confidence interval (CI) was analyzed to examine the trend changes between 1998 and 2019 vs. 2020–2021 (before vs. during the pandemic)^5,12,17,18^. Additionally, we utilized weighted logistic regression models to obtain the weighted odds ratios (ORs) and 95% CI between 1998 to 2019 versus 2020 to 2021. We performed a stratification analysis by sex, educational background, region of residence, and income through all weighted linear and logistic regression models to confirm our main findings. We calculated the ratio of ORs to estimate the interaction term of each risk factor, which allowed us to identify which groups were more vulnerable to the prevalence of diagnosed hypertension and more likely to have awareness, treatment, control, and control among patients receiving treatment during the pandemic. To robust the hypothesis, we analyzed β_diff_ and ORs by a single year between 2016 to 2019 versus 2020 to 2020.

All statistical analyses were performed using the SAS software (version 9.4; SAS Institute, Cary, NC, USA). We used a two-sided test and considered p-values < 0.05 statistically significant.

## Results

Over 24 years, between 1998 and 2021, a comprehensive examination of the KNHANES included 108,687 participants. Table 1 shows the demographic and baseline characteristics of the participants. Of the total participants (mean age 49.97 [standard deviation, 16.78] years), 47,062 (43.3%) were men, whereas 61,625 (56.7%) were women.

**Table 1:** General characteristics^a^ of Korean adults, 1998–2021 (total N = 108,687).

	Total	Pre-pandemic					During the pandemic	
	1998 to 2021	1998 to 2005	2007 to 2009	2010 to 2012	2013 to 2015	2016 to 2019	2020	2021
Overall, n	108,687	19,759	17,287	18,481	17,000	24,753	5812	5595
Sex, n (%)								
Men	47,062 (43.3)	8665 (43.9)	7328 (42.4)	7853 (42.5)	7275 (42.8)	10,871 (43.9)	2601 (44.8)	2469 (44.1)
Women	61,625 (56.7)	11,094 (56.1)	9959 (57.6)	10,628 (57.5)	9725 (57.2)	13,882 (56.1)	3211 (55.2)	3126 (55.9)
Age, years, n (%)								
19 to 29	14,307 (13.2)	3473 (17.6)	2281 (13.2)	2128 (11.5)	2049 (12.1)	2932 (11.8)	788 (13.6)	656 (11.7)
30 to 39	19,511 (18.0)	4715 (23.9)	3476 (20.1)	3372 (18.2)	2728 (16.0)	3832 (15.5)	751 (12.9)	637 (11.4)
40 to 49	20,511 (18.9)	4354 (22.0)	3388 (19.6)	3277 (17.7)	3078 (18.1)	4512 (18.2)	957 (16.5)	945 (16.9)
50 to 59	19,536 (18.0)	3052 (15.4)	2889 (16.7)	3517 (19.0)	3332 (19.6)	4687 (18.9)	1043 (17.9)	1016 (18.2)
60 to 69	17,925 (16.5)	2541 (12.9)	2779 (16.1)	3139 (17.0)	2902 (17.1)	4348 (17.6)	1110 (19.1)	1106 (19.8)
70 to 79	12,920 (11.9)	1311 (6.6)	1994 (11.5)	2471 (13.4)	2237 (13.2)	3227 (13.0)	833 (14.3)	847 (15.1)
≥ 80	3977 (3.7)	313 (1.6)	480 (2.8)	577 (3.1)	674 (4.0)	1215 (4.9)	330 (5.7)	388 (6.9)
Region of residence, n (%)								
Urban	84,054 (77.3)	14,138 (71.6)	12,693 (73.4)	14,608 (79.0)	13,642 (80.2)	20,040 (81.0)	4604 (79.2)	4329 (77.4)
Rural	24,633 (22.7)	5621 (28.4)	4594 (26.6)	3873 (21.0)	3358 (19.8)	4713 (19.0)	1208 (20.8)	1266 (22.6)
Body mass index group, kg/m^2^, n (%)								
Normal or underweight (> 23)	47,534 (43.7)	9310 (47.1)	7630 (44.1)	8294 (44.9)	7397 (43.5)	10,456 (42.2)	2230 (38.4)	2217 (39.6)
Overweight (23 to 25)	25,267 (23.3)	4634 (23.5)	4112 (23.8)	4303 (23.3)	3976 (23.4)	5649 (22.8)	1318 (22.7)	1275 (22.8)
Obese (≥ 25)	35,398 (32.6)	5765 (29.2)	5456 (31.6)	5821 (31.5)	5594 (32.9)	8559 (34.6)	2186 (37.6)	2017 (36.1)
Unknown	488 (0.5)	50 (0.3)	89 (0.5)	63 (0.3)	33 (0.2)	89 (0.4)	78 (1.3)	86 (1.5)
Central obesity^b^, n (%)								
No	77,310 (71.1)	15,002 (75.9)	12,433 (71.9)	13,601 (73.6)	12,449 (73.2)	16,858 (68.1)	3490 (60.0)	3477 (62.1)
Yes	30,992 (28.5)	4706 (23.8)	4757 (27.5)	4805 (26.0)	4518 (26.6)	7822 (31.6)	2298 (39.5)	2086 (37.3)
Unknown	385 (0.4)	51 (0.3)	97 (0.6)	75 (0.4)	33 (0.2)	73 (0.3)	24 (0.4)	32 (0.6)
Educational background, n (%)								
Elementary school or less	29,609 (27.2)	5200 (26.3)	5091 (29.4)	5260 (28.5)	5294 (31.1)	6037 (24.4)	1378 (23.7)	1349 (24.1)
Middle school	11,452 (10.5)	2558 (12.9)	1924 (11.1)	1946 (10.5)	1639 (9.6)	2336 (9.4)	517 (8.9)	532 (9.5)
High school	35,066 (32.3)	6812 (34.5)	5903 (34.1)	5925 (32.1)	5209 (30.6)	7580 (30.6)	1880 (32.3)	1757 (31.4)
College or more	32,560 (30.0)	5189 (26.3)	4369 (25.3)	5350 (28.9)	4858 (28.6)	8800 (35.6)	2037 (35.0)	1957 (35.0)
Income, n (%)								
Lowest quartile	22,008 (20.3)	4262 (21.6)	3613 (20.9)	3645 (19.7)	3388 (19.9)	4861 (19.6)	1106 (19.0)	1133 (20.3)
Second quartile	26,692 (24.6)	4744 (24.0)	4210 (24.4)	4706 (25.5)	4277 (25.2)	6076 (24.5)	1362 (23.4)	1317 (23.5)
Third quartile	28,865 (26.6)	5242 (26.5)	4485 (25.9)	4920 (26.6)	4554 (26.8)	6576 (26.6)	1598 (27.5)	1490 (26.6)
Highest quartile	29,749 (27.4)	5104 (25.8)	4543 (26.3)	4956 (26.8)	4665 (27.4)	7138 (28.8)	1720 (29.6)	1623 (29.0)
Unknown	1373 (1.3)	407 (2.1)	436 (2.5)	254 (1.4)	116 (0.7)	102 (0.4)	26 (0.4)	32 (0.6)
Alcohol consumption, n (%)								
Non-drinker	37,490 (34.5)	9806 (49.6)	5245 (30.3)	5821 (31.5)	5757 (33.9)	7128 (28.8)	1826 (31.4)	1907 (34.1)
1 to 3 times a week	63,265 (58.2)	7971 (40.3)	10,739 (62.1)	11,412 (61.7)	10,194 (60.0)	15,928 (64.3)	3663 (63.0)	3358 (60.0)
≥ 4 times a week	7932 (7.3)	1982 (10.0)	1303 (7.5)	1248 (6.8)	1049 (6.2)	1697 (6.9)	323 (5.6)	330 (5.9)
Smoking status, n (%)								
Non-smoker	66,282 (61.0)	12,251 (62.0)	10,133 (58.6)	11,200 (60.6)	10,800 (63.5)	14,984 (60.5)	3523 (60.6)	3391 (60.6)
Ex-smoker	22,109 (20.3)	2139 (10.8)	3331 (19.3)	3623 (19.6)	3192 (18.8)	5352 (21.6)	1332 (22.9)	1327 (23.7)
Smoker	20,296 (18.7)	5369 (27.2)	3823 (22.1)	3658 (19.8)	3008 (17.7)	4417 (17.8)	957 (16.5)	877 (15.7)

^a^Numbers and unweighted proportions (%).

^b^Central obesity was defined as having waist circumference ≥ 90 cm for men and ≥ 85 cm for women.

Table 2 shows the weighted crude prevalence, awareness, treatment, control, and control among patients receiving treatment stratified by factor groups over 24 years and the trends before and during the COVID-19 pandemic.

**Table 2:** National trend of the weighted crude prevalence, awareness, treatment, control, control among treated before and during the COVID-19 pandemic, 1998–2021.

Weighted % (95% CI)		Pre-pandemic					During the pandemic	
		1998 to 2005	2007 to 2009	2010 to 2012	2013 to 2015	2016 to 2019	2020	2021
Overall	Hypertension	25.51 (24.27 to 26.75)	25.10 (24.12 to 26.08)	27.04 (26.04 to 28.03)	24.39 (23.49 to 25.30)	27.81 (26.97 to 28.66)	29.00 (27.13 to 30.87)	28.07 (26.16 to 29.98)
	Awareness	43.41 (41.17 to 45.65)	58.85 (56.82 to 60.88)	57.98 (56.08 to 59.88)	62.18 (60.29 to 64.07)	68.08 (66.66 to 69.50)	69.50 (67.05 to 71.94)	74.08 (71.34 to 76.81)
	Treatment	38.69 (36.55 to 40.83)	52.61 (50.51 to 54.72)	54.15 (52.28 to 56.03)	59.42 (57.53 to 61.31)	64.92 (63.44 to 66.40)	65.78 (63.13 to 68.43)	71.19 (68.38 to 74.01)
	Control	17.81 (16.08 to 19.54)	33.24 (31.17 to 35.32)	34.57 (32.84 to 36.29)	42.56 (40.73 to 44.38)	47.21 (45.65 to 48.76)	47.43 (44.70 to 50.16)	56.00 (53.09 to 58.91)
	Control among treated	46.04 (42.70 to 49.37)	63.18 (60.61 to 65.76)	63.83 (61.67 to 66.00)	71.62 (69.64 to 73.59)	72.72 (71.18 to 74.25)	72.10 (69.33 to 74.87)	78.65 (76.16 to 81.15)
Sex								
Men	Hypertension	29.99 (28.31 to 31.66)	27.88 (26.53 to 29.22)	30.23 (28.85 to 31.62)	26.91 (25.64 to 28.18)	30.54 (29.48 to 31.61)	33.07 (30.86 to 35.28)	30.06 (27.75 to 32.38)
	Awareness	34.82 (32.03 to 37.61)	49.99 (47.43 to 52.55)	47.43 (44.96 to 49.90)	54.25 (51.60 to 56.91)	62.67 (60.69 to 64.64)	64.72 (61.01 to 68.43)	70.31 (66.42 to 74.21)
	Treatment	29.23 (26.62 to 31.85)	42.62 (40.08 to 45.17)	43.07 (40.67 to 45.47)	50.58 (47.93 to 53.23)	58.80 (56.74 to 60.86)	60.12 (56.32 to 63.91)	66.34 (62.39 to 70.29)
	Control	12.71 (10.69 to 14.72)	26.25 (23.89 to 28.60)	28.26 (26.22 to 30.31)	36.29 (33.94 to 38.65)	44.44 (42.39 to 46.50)	44.46 (40.70 to 48.22)	52.76 (48.59 to 56.93)
	Control among treated	43.47 (38.08 to 48.85)	61.58 (57.78 to 65.38)	65.62 (62.67 to 68.56)	71.75 (68.89 to 74.62)	75.59 (73.51 to 77.67)	73.96 (70.03 to 77.89)	79.53 (75.80 to 83.26)
Women	Hypertension	21.94 (20.51 to 23.38)	22.38 (21.29 to 23.47)	23.93 (22.77 to 25.09)	21.94 (20.87 to 23.00)	25.12 (24.08 to 26.16)	24.98 (22.80 to 27.15)	26.09 (23.85 to 28.33)
	Awareness	52.76 (49.89 to 55.62)	69.67 (67.35 to 71.98)	70.94 (68.71 to 73.17)	71.67 (69.53 to 73.80)	74.59 (72.88 to 76.29)	75.76 (72.63 to 78.88)	78.41 (75.00 to 81.81)
	Treatment	48.97 (46.09 to 51.86)	64.80 (62.35 to 67.26)	67.76 (65.50 to 70.02)	70.00 (67.89 to 72.11)	72.28 (70.55 to 74.01)	73.20 (69.95 to 76.45)	76.79 (73.11 to 80.46)
	Control	23.36 (20.81 to 25.91)	41.78 (39.22 to 44.35)	42.31 (39.87 to 44.75)	50.05 (47.70 to 52.41)	50.53 (48.64 to 52.41)	51.31 (47.38 to 55.24)	59.72 (55.60 to 63.84)
	Control among treated	47.71 (43.55 to 51.86)	64.47 (61.73 to 67.21)	62.44 (59.65 to 65.22)	71.50 (69.03 to 73.98)	69.90 (67.96 to 71.84)	70.10 (66.14 to 74.06)	77.77 (74.18 to 81.37)
Region of residence								
Urban	Hypertension	23.60 (22.30 to 24.91)	23.84 (22.74 to 24.95)	25.44 (24.39 to 26.49)	23.34 (22.35 to 24.32)	26.44 (25.57 to 27.30)	28.11 (26.12 to 30.11)	25.39 (23.58 to 27.21)
	Awareness	41.92 (39.30 to 44.54)	56.99 (54.55 to 59.44)	56.68 (54.43 to 58.93)	61.97 (59.81 to 64.14)	66.36 (64.78 to 67.94)	68.90 (66.14 to 71.66)	73.20 (69.88 to 76.52)
	Treatment	36.96 (34.53 to 39.39)	50.56 (48.00 to 53.11)	52.16 (49.95 to 54.37)	58.98 (56.80 to 61.15)	62.99 (61.34 to 64.64)	64.97 (61.95 to 67.98)	70.51 (67.06 to 73.96)
	Control	17.01 (15.09 to 18.92)	32.21 (29.69 to 34.73)	33.18 (31.26 to 35.09)	42.37 (40.31 to 44.43)	45.69 (44.03 to 47.35)	46.95 (43.84 to 50.06)	55.87 (52.48 to 59.25)
	Control among treated	46.02 (42.12 to 49.91)	63.72 (60.50 to 66.93)	63.60 (61.04 to 66.17)	71.85 (69.61 to 74.09)	72.53 (70.80 to 74.27)	72.27 (69.09 to 75.45)	79.23 (76.47 to 81.99)
Rural	Hypertension	32.27 (29.44 to 35.09)	30.37 (27.88 to 32.86)	33.43 (30.97 to 35.90)	29.30 (26.79 to 31.82)	35.38 (32.60 to 38.16)	33.81 (28.67 to 38.95)	41.97 (37.11 to 46.83)
	Awareness	47.27 (43.06 to 51.49)	64.97 (61.96 to 67.97)	61.96 (58.39 to 65.53)	62.93 (59.06 to 66.81)	75.14 (72.19 to 78.09)	72.19 (67.25 to 77.13)	76.82 (72.42 to 81.22)
	Treatment	43.16 (38.84 to 47.48)	59.39 (56.28 to 62.49)	60.23 (56.69 to 63.77)	61.07 (57.06 to 65.08)	72.83 (69.71 to 75.94)	69.45 (64.45 to 74.44)	73.35 (68.95 to 77.76)
	Control	19.89 (16.18 to 23.60)	36.64 (33.40 to 39.88)	38.81 (34.94 to 42.67)	43.23 (39.15 to 47.31)	53.43 (49.66 to 57.20)	49.56 (44.12 to 55.01)	56.41 (50.63 to 62.18)
	Control among treated	46.08 (39.74 to 52.43)	61.69 (57.83 to 65.55)	64.43 (60.29 to 68.58)	70.79 (66.61 to 74.97)	73.37 (70.12 to 76.61)	71.37 (65.92 to 76.82)	76.90 (71.38 to 82.41)
Age group								
19 to 59	Hypertension	17.72 (16.64 to 18.80)	17.93 (17.00 to 18.86)	18.82 (17.91 to 19.74)	16.14 (15.29 to 16.99)	17.96 (17.22 to 18.71)	18.83 (17.30 to 20.36)	16.69 (15.13 to 18.26)
	Awareness	33.01 (30.40 to 35.62)	45.63 (42.97 to 48.29)	41.89 (39.22 to 44.56)	44.93 (42.20 to 47.66)	50.95 (48.77 to 53.12)	52.61 (48.65 to 56.56)	60.07 (55.27 to 64.87)
	Treatment	27.25 (24.78 to 29.73)	37.91 (35.33 to 40.49)	36.52 (33.96 to 39.08)	41.20 (38.52 to 43.89)	46.64 (44.44 to 48.84)	46.77 (42.67 to 50.88)	56.45 (51.63 to 61.26)
	Control	13.81 (11.85 to 15.77)	23.56 (21.18 to 25.93)	22.61 (20.57 to 24.65)	29.10 (26.66 to 31.53)	34.34 (32.23 to 36.46)	34.07 (30.17 to 37.98)	47.60 (42.64 to 52.55)
	Control among treated	50.68 (45.60 to 55.75)	62.14 (58.13 to 66.16)	61.92 (58.01 to 65.83)	70.62 (67.07 to 74.18)	73.63 (70.90 to 76.37)	72.85 (67.73 to 77.97)	84.32 (79.51 to 89.14)
≥ 60	Hypertension	56.46 (54.17 to 58.75)	55.84 (54.05 to 57.64)	59.15 (57.58 to 60.72)	53.56 (51.85 to 55.27)	57.68 (56.40 to 58.96)	55.91 (53.22 to 58.59)	56.15 (53.64 to 58.66)
	Awareness	56.37 (53.30 to 59.45)	77.05 (75.04 to 79.06)	78.01 (76.05 to 79.97)	80.54 (78.88 to 82.21)	84.25 (83.03 to 85.48)	84.55 (82.27 to 86.83)	84.34 (81.91 to 86.78)
	Treatment	52.95 (49.84 to 56.05)	72.85 (70.77 to 74.94)	76.10 (74.13 to 78.07)	78.82 (77.11 to 80.53)	82.17 (80.87 to 83.47)	82.72 (80.29 to 85.15)	82.01 (79.57 to 84.45)
	Control	22.80 (20.07 to 25.53)	46.58 (43.81 to 49.35)	49.45 (47.04 to 51.85)	56.89 (54.69 to 59.08)	59.35 (57.55 to 61.14)	59.33 (55.77 to 62.88)	62.16 (58.81 to 65.50)
	Control among treated	43.06 (38.99 to 47.13)	63.93 (61.04 to 66.82)	64.97 (62.46 to 67.49)	72.17 (69.93 to 74.42)	72.22 (70.45 to 74.00)	71.72 (68.38 to 75.06)	75.79 (72.79 to 78.79)
Educational background								
High school or lower	Hypertension	30.70 (29.21 to 32.20)	29.34 (28.19 to 30.49)	31.99 (30.81 to 33.16)	28.98 (27.86 to 30.10)	34.66 (33.58 to 35.73)	35.37 (33.11 to 37.62)	35.92 (33.64 to 38.20)
	Awareness	46.77 (44.37 to 49.16)	61.74 (59.68 to 63.80)	62.29 (60.35 to 64.23)	66.00 (64.05 to 67.95)	73.27 (71.80 to 74.73)	74.39 (71.52 to 77.27)	78.27 (75.63 to 80.90)
	Treatment	41.83 (39.50 to 44.15)	55.53 (53.43 to 57.63)	59.08 (57.14 to 61.02)	63.60 (61.63 to 65.57)	70.31 (68.79 to 71.82)	71.67 (68.87 to 74.46)	75.48 (72.75 to 78.21)
	Control	19.26 (17.37 to 21.15)	35.05 (32.91 to 37.18)	38.13 (36.20 to 40.05)	45.61 (43.60 to 47.62)	50.60 (48.94 to 52.26)	51.94 (48.76 to 55.11)	58.47 (55.54 to 61.39)
	Control among treated	46.06 (42.65 to 49.46)	63.12 (60.44 to 65.79)	64.54 (62.29 to 66.78)	71.71 (69.57 to 73.85)	71.97 (70.34 to 73.59)	72.47 (69.25 to 75.69)	77.46 (74.82 to 80.09)
College or higher	Hypertension	13.95 (12.61 to 15.28)	14.82 (13.37 to 16.26)	15.98 (14.71 to 17.26)	14.75 (13.53 to 15.96)	17.43 (16.50 to 18.37)	19.15 (17.11 to 21.20)	16.83 (14.85 to 18.82)
	Awareness	26.95 (22.41 to 31.49)	44.97 (40.36 to 49.59)	38.76 (34.83 to 42.68)	46.38 (41.94 to 50.83)	52.43 (49.59 to 55.27)	55.51 (50.37 to 60.65)	61.26 (55.26 to 67.25)
	Treatment	23.30 (19.19 to 27.40)	38.60 (33.74 to 43.46)	32.14 (28.43 to 35.85)	42.15 (37.94 to 46.36)	48.67 (45.72 to 51.61)	48.96 (43.45 to 54.48)	58.09 (51.85 to 64.34)
	Control	10.69 (7.46 to 13.91)	24.57 (19.97 to 29.18)	18.66 (15.92 to 21.40)	29.95 (26.20 to 33.70)	36.98 (34.12 to 39.83)	34.54 (29.56 to 39.53)	48.44 (42.03 to 54.86)
	Control among treated	45.87 (35.53 to 56.21)	63.66 (56.34 to 70.99)	58.06 (51.62 to 64.49)	71.05 (65.77 to 76.33)	75.98 (72.68 to 79.27)	70.55 (63.82 to 77.28)	83.39 (77.31 to 89.47)
Income								
Lowest or second quartile	Hypertension	31.14 (29.34 to 32.94)	31.86 (30.44 to 33.28)	33.04 (31.54 to 34.54)	31.48 (30.03 to 32.92)	36.34 (35.06 to 37.62)	37.97 (35.06 to 40.88)	38.15 (35.28 to 41.02)
	Awareness	47.49 (44.75 to 50.24)	63.27 (60.83 to 65.72)	64.27 (61.91 to 66.63)	69.01 (66.73 to 71.30)	75.17 (73.47 to 76.87)	76.79 (73.60 to 79.98)	80.24 (77.26 to 83.21)
	Treatment	43.05 (40.28 to 45.83)	57.45 (54.96 to 59.95)	61.00 (58.59 to 63.41)	66.86 (64.57 to 69.16)	71.84 (70.01 to 73.67)	74.23 (70.96 to 77.50)	77.32 (74.18 to 80.45)
	Control	19.21 (16.84 to 21.58)	35.61 (33.19 to 38.03)	39.07 (36.68 to 41.45)	47.51 (45.15 to 49.86)	51.40 (49.44 to 53.36)	52.03 (48.39 to 55.67)	59.13 (55.41 to 62.86)
	Control among treated	44.62 (40.49 to 48.74)	61.99 (59.03 to 64.95)	64.04 (61.46 to 66.63)	71.05 (68.56 to 73.53)	71.55 (69.64 to 73.46)	70.09 (66.22 to 73.97)	76.48 (73.00 to 79.97)
Third or highest quartile	Hypertension	20.29 (19.03 to 21.55)	20.18 (19.03 to 21.34)	22.33 (21.22 to 23.44)	19.57 (18.55 to 20.59)	22.05 (21.17 to 22.93)	23.68 (21.79 to 25.57)	22.20 (20.48 to 23.92)
	Awareness	38.72 (35.45 to 41.99)	53.05 (50.14 to 55.96)	51.02 (48.28 to 53.76)	54.62 (51.93 to 57.31)	60.30 (58.26 to 62.33)	62.85 (59.40 to 66.30)	67.74 (63.46 to 72.03)
	Treatment	33.41 (30.43 to 36.40)	46.24 (43.16 to 49.33)	46.50 (43.79 to 49.20)	51.36 (48.69 to 54.03)	57.30 (55.21 to 59.39)	58.01 (54.31 to 61.70)	64.91 (60.55 to 69.27)
	Control	16.36 (13.96 to 18.76)	29.68 (26.73 to 32.62)	29.56 (27.28 to 31.83)	37.22 (34.80 to 39.65)	42.64 (40.54 to 44.74)	43.20 (39.53 to 46.87)	52.69 (48.00 to 57.39)
	Control among treated	48.97 (43.81 to 54.13)	64.18 (60.16 to 68.19)	63.56 (59.92 to 67.21)	72.48 (69.47 to 75.49)	74.41 (72.11 to 76.72)	74.48 (70.32 to 78.63)	81.17 (77.32 to 85.03)

*CI* confidence interval.

### Prevalence of hypertension

**Figure 1:**
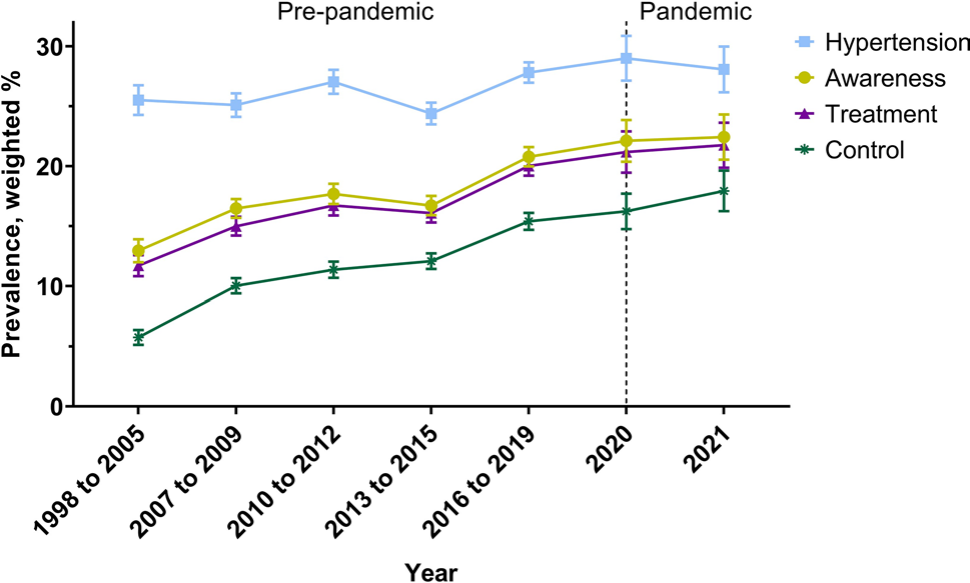
Nationwide trends in hypertension prevalence, awareness, treatment, and control among Korean adults, 1998–2021. Markers represent the mean ratio. The horizontal lines indicate 95% confidence interval.

The study found that there was a statistically significant increase in the weighted prevalence of hypertension from 25.51% (95% CI: 24.27–26.75) to 28.07% (95% CI: 26.16–29.98) between 1998 and 2021 (Table 2 and Table S1–S14). However, the prevalence of hypertension did not increase significantly from the new onset of the pandemic to 2021. Regarding sex differences, the prevalence of hypertension significantly increased in men during the pandemic compared to the pre-pandemic era of 2016–2019 (Table S15 and S16). However, no significant differences were observed in the age group, residence, educational background, or income categories. Table 3 shows the odds ratios (95% CI) and coefficients of hypertension prevalence, awareness, treatment, control, and control among patients receiving treatment during the pre- and pandemic eras. Before the pandemic, the prevalence of hypertension consistently increased between 1998 and 2019. However, the increase in the prevalence of hypertension showed a decrease during the pandemic period compared to the pre-pandemic era (β_diff_, −0.012; 95% CI: −0.023 to 0.000, p-value < 0.05). This trend was observed in urban areas and across different levels of education (Fig. 1).

**Table 3:** Weighed trend of hypertension prevalence, awareness, treatment, control, and control among treated: estimated β-coefficients and weighted odds ratios with 95% CIs.

		Weighted trends in prevalence, awareness, treatment and control of hypertension			Weighted odds of before and during the pandemic, OR	
		Pre-pandemic era (1998 to 2019), β (95% CI)	Pandemic era (2020 to 2021), β (95% CI)	Trend difference, β diff (95% CI)	2020 versus 1998 to 2019	2021 versus 2020
Overall	Hypertension	**0.014 (0.002 to 0.009)**	0.002 (−0.009 to 0.012)	**−0.012 (−0.023 to 0.000)**	**1.15 (1.05 to 1.26)**	0.96 (0.83 to 1.10)
	Awareness	**0.098 (0.033 to 0.046)**	**0.054 (0.015 to 0.046)**	**−0.044 (−0.061 to −0.028)**	**1.45 (1.29 to 1.64)**	**1.25 (1.04 to 1.51)**
	Treatment	**0.048 (0.041 to 0.055)**	**0.031 (0.015 to 0.047)**	−0.017 (−0.034 to 0.000)	**1.44 (1.27 to 1.63)**	**1.29 (1.07 to 1.54)**
	Control	**0.139 (0.049 to 0.063)**	**0.072 (0.028 to 0.061)**	**−0.067 (−0.085 to −0.049)**	**1.43 (1.27 to 1.60)**	**1.41 (1.20 to 1.66)**
	Control among treated	**0.042 (0.034 to 0.050)**	**0.030 (0.016 to 0.045)**	−0.012 (−0.029 to 0.005)	**1.23 (1.07 to 1.42)**	**1.43 (1.17 to 1.74)**
Sex						
Men	Hypertension	0.009 (−0.001 to 0.008)	−0.004 (−0.016 to 0.010)	−0.014 (−0.027 to 0.000)	**1.20 (1.08 to 1.33)**	0.87 (0.75 to 1.01)
	Awareness	**0.123 (0.042 to 0.060)**	**0.065 (0.016 to 0.060)**	**−0.058 (−0.082 to −0.035)**	**1.61 (1.36 to 1.91)**	**1.29 (1.01 to 1.66)**
	Treatment	**0.061 (0.052 to 0.069)**	**0.038 (0.016 to 0.060)**	−0.023 (−0.047 to 0.001)	**1.59 (1.35 to 1.88)**	**1.31 (1.03 to 1.66)**
	Control	**0.170 (0.058 to 0.075)**	**0.067 (0.018 to 0.065)**	**−0.103 (−0.128 to −0.078)**	**1.58 (1.35 to 1.85)**	**1.40 (1.11 to 1.75)**
	Control among treated	**0.053 (0.042 to 0.065)**	0.021 (−0.001 to 0.042)	**−0.032 (−0.056 to −0.008)**	**1.26 (1.02 to 1.57)**	**1.37 (1.01 to 1.86)**
Women	Hypertension	**0.018 (0.002 to 0.010)**	0.009 (−0.008 to 0.017)	−0.009 (−0.022 to 0.005)	1.09 (0.97 to 1.23)	1.06 (0.90 to 1.26)
	Awareness	**0.069 (0.018 to 0.034)**	0.037 (0.000 to 0.038)	**−0.032 (−0.053 to −0.011)**	**1.31 (1.10 to 1.56)**	1.16 (0.89 to 1.52)
	Treatment	**0.034 (0.026 to 0.042)**	**0.023 (0.002 to 0.043)**	−0.011 (−0.033 to 0.011)	**1.31 (1.10 to 1.55)**	1.21 (0.93 to 1.58)
	Control	**0.105 (0.035 to 0.052)**	**0.076 (0.023 to 0.069)**	**−0.029 (−0.054 to −0.005)**	**1.30 (1.10 to 1.52)**	**1.41 (1.12 to 1.77)**
	Control among treated	**0.032 (0.023 to 0.041)**	**0.040 (0.019 to 0.061)**	0.008 (−0.015 to 0.031)	1.19 (0.98 to 1.44)	**1.49 (1.13 to 1.98)**
Region of residence						
Urban	Hypertension	0.016 (−0.002 to 0.013)	−0.004 (−0.019 to 0.015)	**−0.020 (−0.039 to −0.002)**	1.11 (0.94 to 1.32)	0.87 (0.76 to 1.00)
	Awareness	**0.085 (0.020 to 0.050)**	0.046 (−0.014 to 0.066)	−0.039 (−0.081 to 0.004)	**1.45 (1.05 to 2.00)**	1.23 (1.00 to 1.53)
	Treatment	**0.045 (0.029 to 0.062)**	0.030 (−0.010 to 0.071)	−0.015 (−0.059 to 0.029)	**1.47 (1.28 to 1.69)**	**1.29 (1.04 to 1.59)**
	Control	**0.114 (0.030 to 0.063)**	0.060 (−0.007 to 0.081)	**−0.054 (−0.101 to −0.006)**	**1.62 (1.22 to 2.14)**	**1.43 (1.19 to 1.72)**
	Control among treated	**0.027 (0.008 to 0.047)**	0.021 (−0.015 to 0.057)	−0.006 (−0.047 to 0.035)	**1.23 (1.04 to 1.45)**	**1.46 (1.16 to 1.84)**
Rural	Hypertension	−0.009 (−0.016 to 0.009)	−0.008 (−0.030 to 0.022)	0.001 (−0.028 to 0.029)	0.98 (0.80 to 1.22)	**1.42 (1.04 to 1.93)**
	Awareness	**0.151 (0.037 to 0.083)**	0.079 (−0.007 to 0.090)	**−0.072 (−0.125 to −0.018)**	**1.76 (1.16 to 2.69)**	1.28 (0.89 to 1.83)
	Treatment	**0.079 (0.054 to 0.103)**	0.043 (−0.005 to 0.091)	−0.036 (−0.090 to 0.018)	**1.39 (1.08 to 1.78)**	1.21 (0.87 to 1.69)
	Control	**0.218 (0.060 to 0.110)**	**0.154 (0.047 to 0.139)**	**−0.064 (−0.116 to −0.011)**	1.44 (0.90 to 2.29)	1.32 (0.95 to 1.82)
	Control among treated	**0.062 (0.032 to 0.092)**	**0.082 (0.037 to 0.127)**	0.020 (−0.034 to 0.074)	1.23 (0.93 to 1.63)	1.34 (0.88 to 2.03)
Age group, years						
19 to 59	Hypertension	−0.007 (−0.005 to 0.001)	−0.013 (−0.015 to 0.002)	−0.007 (−0.016 to 0.003)	1.08 (0.97 to 1.20)	0.86 (0.74 to 1.01)
	Awareness	**0.060 (0.016 to 0.034)**	**0.073 (0.019 to 0.071)**	0.013 (−0.015 to 0.041)	**1.35 (1.15 to 1.59)**	**1.36 (1.05 to 1.75)**
	Treatment	**0.035 (0.026 to 0.044)**	**0.048 (0.022 to 0.074)**	0.013 (−0.015 to 0.041)	**1.32 (1.11 to 1.57)**	**1.48 (1.14 to 1.91)**
	Control	**0.113 (0.033 to 0.050)**	**0.108 (0.039 to 0.091)**	−0.005 (−0.033 to 0.022)	**1.41 (1.17 to 1.69)**	**1.76 (1.35 to 2.28)**
	Control among treated	**0.046 (0.032 to 0.059)**	**0.054 (0.026 to 0.082)**	0.008 (−0.023 to 0.039)	**1.31 (1.00 to 1.71)**	**2.00 (1.28 to 3.13)**
≥ 60	Hypertension	0.001 (−0.006 to 0.006)	−0.012 (−0.022 to 0.007)	−0.013 (−0.028 to 0.003)	0.97 (0.87 to 1.08)	1.01 (0.87 to 1.17)
	Awareness	**0.107 (0.030 to 0.042)**	0.001 (−0.013 to 0.014)	**−0.106 (−0.121 to −0.091)**	**1.46 (1.21 to 1.76)**	0.98 (0.76 to 1.27)
	Treatment	**0.041 (0.035 to 0.048)**	−0.001 (−0.015 to 0.013)	**−0.042 (−0.057 to −0.027)**	**1.47 (1.23 to 1.75)**	0.95 (0.75 to 1.21)
	Control	**0.138 (0.049 to 0.066)**	0.024 (−0.005 to 0.033)	**−0.114 (−0.135 to −0.093)**	**1.34 (1.15 to 1.57)**	1.13 (0.92 to 1.38)
	Control among treated	**0.040 (0.030 to 0.049)**	**0.018 (0.001 to 0.036)**	**−0.022 (−0.042 to −0.002)**	**1.19 (1.01 to 1.42)**	1.23 (0.98 to 1.56)
Educational background						
High school or less	Hypertension	**0.028 (0.007 to 0.015)**	0.011 (−0.007 to 0.019)	**−0.017 (−0.031 to −0.004)**	**1.19 (1.07 to 1.33)**	1.03 (0.89 to 1.19)
	Awareness	**0.114 (0.038 to 0.052)**	**0.047 (0.010 to 0.040)**	**−0.067 (−0.083 to −0.050)**	**1.56 (1.34 to 1.82)**	1.24 (1.00 to 1.54)
	Treatment	**0.054 (0.048 to 0.061)**	**0.026 (0.010 to 0.042)**	**−0.028 (−0.045 to −0.011)**	**1.59 (1.38 to 1.84)**	1.22 (0.99 to 1.49)
	Control	**0.148 (0.053 to 0.068)**	**0.064 (0.022 to 0.057)**	**−0.083 (−0.102 to −0.065)**	**1.53 (1.34 to 1.75)**	**1.30 (1.09 to 1.56)**
	Control among treated	**0.040 (0.031 to 0.048)**	**0.028 (0.012 to 0.043)**	−0.012 (−0.030 to 0.006)	**1.27 (1.08 to 1.50)**	**1.31 (1.05 to 1.63)**
College or more	Hypertension	**0.024 (0.003 to 0.012)**	−0.007 (−0.014 to 0.008)	**−0.031 (−0.043 to −0.019)**	**1.25 (1.09 to 1.43)**	0.85 (0.71 to 1.03)
	Awareness	**0.098 (0.026 to 0.054)**	**0.072 (0.011 to 0.077)**	−0.026 (−0.062 to 0.010)	**1.48 (1.19 to 1.84)**	1.27 (0.92 to 1.76)
	Treatment	**0.048 (0.034 to 0.063)**	**0.047 (0.013 to 0.082)**	−0.001 (−0.038 to 0.036)	**1.38 (1.09 to 1.74)**	**1.45 (1.03 to 2.04)**
	Control	**0.149 (0.042 to 0.069)**	**0.095 (0.023 to 0.093)**	**−0.054 (−0.091 to −0.016)**	**1.33 (1.05 to 1.68)**	**1.78 (1.27 to 2.49)**
	Control among treated	**0.056 (0.034 to 0.077)**	**0.040 (0.004 to 0.076)**	−0.016 (−0.058 to 0.026)	1.06 (0.75 to 1.49)	**2.10 (1.22 to 3.61)**
Income						
Lowest or second quartile	Hypertension	**0.029 (0.006 to 0.017)**	0.015 (−0.007 to 0.025)	−0.014 (−0.030 to 0.003)	**1.23 (1.08 to 1.39)**	1.01 (0.85 to 1.20)
	Awareness	**0.124 (0.040 to 0.056)**	**0.050 (0.008 to 0.043)**	**−0.075 (−0.094 to −0.056)**	**1.62 (1.35 to 1.94)**	1.23 (0.94 to 1.60)
	Treatment	**0.055 (0.047 to 0.063)**	**0.027 (0.009 to 0.045)**	**−0.028 (−0.048 to −0.008)**	1.65 (1.38 to 1.97)	1.18 (0.92 to 1.52)
	Control	**0.153 (0.054 to 0.071)**	**0.063 (0.018 to 0.060)**	**−0.090 (−0.112 to −0.067)**	**1.47 (1.26 to 1.71)**	**1.33 (1.08 to 1.65)**
	Control among treated	**0.042 (0.032 to 0.051)**	**0.025 (0.005 to 0.045)**	−0.017 (−0.039 to 0.005)	1.16 (0.96 to 1.41)	**1.39 (1.06 to 1.81)**
Third or highest quartile	Hypertension	0.009 (−0.001 to 0.007)	0.001 (−0.009 to 0.011)	−0.008 (−0.018 to 0.003)	**1.16 (1.04 to 1.30)**	0.92 (0.79 to 1.07)
	Awareness	**0.077 (0.023 to 0.042)**	**0.062 (0.014 to 0.061)**	−0.015 (−0.041 to 0.011)	**1.42 (1.22 to 1.66)**	1.24 (0.97 to 1.58)
	Treatment	**0.043 (0.033 to 0.053)**	**0.038 (0.014 to 0.063)**	−0.005 (−0.031 to 0.021)	**1.37 (1.17 to 1.61)**	**1.34 (1.05 to 1.71)**
	Control	**0.129 (0.042 to 0.061)**	**0.082 (0.025 to 0.077)**	**−0.047 (−0.075 to −0.020)**	**1.44 (1.23 to 1.68)**	**1.46 (1.16 to 1.86)**
	Control among treated	**0.044 (0.031 to 0.057)**	**0.035 (0.012 to 0.058)**	−0.009 (−0.035 to 0.017)	**1.31 (1.04 to 1.65)**	**1.48 (1.06 to 2.06)**

*CI* confidence interval.

Numbers in bold indicate a significant difference (*P* < 0.05).

### Hypertension awareness, treatment, control, and control among patients receiving treatment

The weighted crude rate of awareness steadily increased from 43.41% (95% CI: 41.17–45.65) to 68.08% (95% CI: 66.66–69.50) before the pandemic (1998–2019) and further improved to 74.08% (95% CI: 71.34–76.81) in 2021. The weighted crude rate of treatment also increased from 38.69% (95% CI: 36.55–40.83) to 64.92% (95% CI: 63.44–66.40) before the pandemic and further improved to 71.19% (95% CI: 68.38–74.01) in 2021. The control rate rose from 17.81% (95% CI: 16.08–19.54) to 47.21% (95% CI: 45.65–48.76) pre-pandemic and subsequently improved to 56.00% (95% CI: 53.09–58.91) in 2021. The proportion of control among the treated population increased from 46.04% (95% CI: 42.70–49.37) to 72.72% (95% CI: 71.18–74.25) before the pandemic and further improved to 78.65% (95% CI: 76.16–81.15) in 2021. During the pandemic, the proportion of controls among the treated population increased, and awareness and treatment rates remained high. The rates of hypertension awareness, treatment, control, and control among patients receiving treatment showed a consistent increase between 1998 and 2019, before the pandemic. However, these indicators increased at a slower pace during the pandemic, as compared to the pre-pandemic era (β_diff_ for awareness, −0.044; 95% CI: [−0.061] to [−0.028], β_diff_ for treatment, −0.017; 95% CI: [−0.034] to [0.000], β_diff_ for control, −0.067; 95% CI: [−0.085] to [−0.049], and β_diff_ for control among treated individuals, −0.012; 95% CI: [−0.029] to [0.005]).

When comparing the pre-pandemic era (2016–2019) to the pandemic era, no significant odds ratios were observed for prevalence, awareness, treatment, control, and control among patients receiving treatment, except for men, who showed a significant odds ratio for the prevalence of hypertension (Table S6). However, during the pandemic era, there were significant increases in hypertension awareness, treatment, control, and control among patients receiving treatment. Regarding health indices, the odds ratios for awareness, treatment, control, and control among patients receiving treatment differed significantly between the first and late pandemic eras. Nevertheless, no significant odds ratios were observed for the prevalence of hypertension during the pandemic era compared with the pre-pandemic era or between the first and late pandemic eras.

### Socioeconomic factors

Table 4 shows the odds ratios between the pre-COVID-19 and COVID-19 pandemics to identify any changes in risk factors during the pandemic era. The risk factors for hypertension were sex (male), older age (> 60 years), residence in rural areas, lower educational status (high school or lower), and higher income (third or highest quartile). No significant differences were observed in socioeconomic factors between the pre-pandemic and pandemic eras. Specifically, men had significantly higher rates of hypertension awareness, treatment, and control among patients receiving treatment during the pandemic compared to the pre-pandemic era. Additionally, individuals aged 19–59 years had significantly higher control rates and control among the treatment group during the pandemic era.

**Table 4:** Ratio of ORs for association between the prevalence, awareness, treatment, control, and control among treated of hypertension and each socioeconomic factor, 1998–2021.

Variables	Overall (1998 to 2021)		Pre-COVID-19 pandemic (1998 to 2019)		COVID-19 pandemic (2020 to 2021)		Ratio of OR (95% CI)	p-value
	Weighted OR (95% CI)	p-value	Weighted OR (95% CI)	p-value	Weighted OR (95% CI)	p-value		
Hypertension								
Sex								
Women	1.00 (reference)		1.00 (reference)		1.00 (reference)		1.00 (reference)	
Men	**1.35 (1.30 to 1.39)**	** < 0.001**	**1.35 (1.30 to 1.40)**	** < 0.001**	**1.34 (1.23 to 1.46)**	** < 0.001**	0.99 (0.91 to 1.09)	0.910
Age, years								
≥ 60	1.00 (reference)		1.00 (reference)		1.00 (reference)		1.00 (reference)	
19 to 59	**0.17 (0.16 to 0.17)**	** < 0.001**	**0.17 (0.16 to 0.17)**	** < 0.001**	**0.17 (0.15 to 0.19)**	** < 0.001**	1.02 (0.92 to 1.13)	0.646
Region								
Rural	1.00 (reference)		1.00 (reference)		1.00 (reference)		1.00 (reference)	
Urban	**0.68 (0.64 to 0.72)**	** < 0.001**	**0.69 (0.65 to 0.74)**	** < 0.001**	**0.59 (0.50 to 0.70)**	** < 0.001**	0.86 (0.71 to 1.03)	0.095
Education								
College or higher	1.00 (reference)		1.00 (reference)		1.00 (reference)		1.00 (reference)	
High school or lower	**2.43 (2.32 to 2.53)**	** < 0.001**	**2.42 (2.31 to 2.54)**	** < 0.001**	**2.54 (2.26 to 2.85)**	** < 0.001**	1.05 (0.92 to 1.18)	0.470
Income								
Third or highest quartile	1.00 (reference)		1.00 (reference)		1.00 (reference)		1.00 (reference)	
Lowest or second quartile	**1.89 (1.82 to 1.96)**	** < 0.001**	**1.87 (1.80 to 1.94)**	** < 0.001**	**2.07 (1.87 to 2.29)**	** < 0.001**	1.11 (0.99 to 1.23)	0.068
Awareness								
Sex								
Women	1.00 (reference)		1.00 (reference)		1.00 (reference)		1.00 (reference)	
Men	**0.49 (0.47 to 0.52)**	** < 0.001**	**0.48 (0.45 to 0.51)**	** < 0.001**	**0.62 (0.51 to 0.74)**	** < 0.001**	**1.29 (1.06 to 1.56)**	**0.010**
Age, years								
≥ 60	1.00 (reference)		1.00 (reference)		1.00 (reference)		1.00 (reference)	
19 to 59	**0.22 (0.21 to 0.23)**	** < 0.001**	**0.22 (0.21 to 0.24)**	** < 0.001**	**0.24 (0.20 to 0.28)**	** < 0.001**	1.08 (0.90 to 1.30)	0.404
Region								
Rural	1.00 (reference)		1.00 (reference)		1.00 (reference)		1.00 (reference)	
Urban	**0.81 (0.75 to 0.88)**	** < 0.001**	**0.81 (0.74 to 0.88)**	** < 0.001**	0.82 (0.67 to 1.01)	0.058	1.02 (0.82 to 1.27)	0.879
Education								
College or higher	1.00 (reference)		1.00 (reference)		1.00 (reference)		1.00 (reference)	
High school or lower	**2.16 (2.01 to 2.32)**	** < 0.001**	**2.20 (2.04 to 2.38)**	** < 0.001**	**2.31 (1.90 to 2.80)**	** < 0.001**	1.05 (0.85 to 1.29)	0.657
Income								
Third or highest quartile	1.00 (reference)		1.00 (reference)		1.00 (reference)		1.00 (reference)	
Lowest or second quartile	**1.72 (1.62 to 1.83)**	** < 0.001**	**1.72 (1.61 to 1.83)**	** < 0.001**	**1.95 (1.64 to 2.31)**	** < 0.001**	1.13 (0.94 to 1.36)	0.180
Treatment								
Sex								
Women	1.00 (reference)		1.00 (reference)		1.00 (reference)		1.00 (reference)	
Men	**0.47 (0.44 to 0.50)**	** < 0.001**	**0.45 (0.43 to 0.48)**	** < 0.001**	**0.57 (0.48 to 0.68)**	** < 0.001**	**1.26 (1.04 to 1.52)**	**0.015**
Age, years								
≥ 60	1.00 (reference)		1.00 (reference)		1.00 (reference)		1.00 (reference)	
19 to 59	**0.21 (0.19 to 0.22)**	** < 0.001**	**0.20 (0.19 to 0.22)**	** < 0.001**	**0.23 (0.19 to 0.27)**	** < 0.001**	1.11 (0.93 to 1.33)	0.246
Region								
Rural	1.00 (reference)		1.00 (reference)		1.00 (reference)		1.00 (reference)	
Urban	**0.78 (0.72 to 0.85)**	** < 0.001**	**0.77 (0.71 to 0.84)**	** < 0.001**	0.83 (0.68 to 1.00)	0.051	1.07 (0.87 to 1.32)	0.513
Education								
College or higher	1.00 (reference)		1.00 (reference)		1.00 (reference)		1.00 (reference)	
High school or lower	**2.25 (2.09 to 2.42)**	** < 0.001**	**2.29 (2.11 to 2.48)**	** < 0.001**	**2.43 (2.01 to 2.93)**	** < 0.001**	1.06 (0.86 to 1.30)	0.575
Income								
Third or highest quartile	1.00 (reference)		1.00 (reference)		1.00 (reference)		1.00 (reference)	
Lowest or second quartile	**1.74 (1.64 to 1.85)**	** < 0.001**	**1.74 (1.63 to 1.85)**	** < 0.001**	**1.97 (1.67 to 2.32)**	** < 0.001**	1.13 (0.95 to 1.35)	0.167
Control								
Sex								
Women	1.00 (reference)		1.00 (reference)		1.00 (reference)		1.00 (reference)	
Men	**0.64 (0.61 to 0.68)**	** < 0.001**	**0.62 (0.59 to 0.66)**	** < 0.001**	**0.75 (0.64 to 0.88)**	**0.001**	**1.20 (1.01 to 1.43)**	**0.037**
Age, years								
≥ 60	1.00 (reference)		1.00 (reference)		1.00 (reference)		1.00 (reference)	
19 to 59	**0.35 (0.33 to 0.37)**	** < 0.001**	**0.34 (0.32 to 0.36)**	** < 0.001**	**0.44 (0.37 to 0.52)**	** < 0.001**	**1.30 (1.09 to 1.55)**	**0.003**
Region								
Rural	1.00 (reference)		1.00 (reference)		1.00 (reference)		1.00 (reference)	
Urban	**0.87 (0.80 to 0.95)**	**0.001**	**0.86 (0.78 to 0.94)**	**0.001**	0.92 (0.76 to 1.11)	0.361	1.07 (0.86 to 1.32)	0.558
Education								
College or higher	1.00 (reference)		1.00 (reference)		1.00 (reference)		1.00 (reference)	
High school or lower	**1.72 (1.59 to 1.86)**	** < 0.001**	**1.77 (1.63 to 1.93)**	** < 0.001**	**1.75 (1.45 to 2.10)**	** < 0.001**	0.98 (0.80 to 1.21)	0.883
Income								
Third or highest quartile	1.00 (reference)		1.00 (reference)		1.00 (reference)		1.00 (reference)	
Lowest or second quartile	**1.37 (1.30 to 1.46)**	** < 0.001**	**1.40 (1.31 to 1.49)**	** < 0.001**	**1.36 (1.17 to 1.60)**	** < 0.001**	0.98 (0.82 to 1.16)	0.783
Control among treated								
Sex								
Women	1.00 (reference)		1.00 (reference)		1.00 (reference)		1.00 (reference)	
Men	**1.15 (1.07 to 1.24)**	** < 0.001**	**1.14 (1.06 to 1.23)**	**0.001**	1.15 (0.93 to 1.43)	0.192	1.01 (0.81 to 1.27)	0.922
Age, years								
≥ 60	1.00 (reference)		1.00 (reference)		1.00 (reference)		1.00 (reference)	
19 to 59	1.00 (0.92 to 1.09)	0.984	0.97 (0.88 to 1.05)	0.424	**1.33 (1.03 to 1.70)**	**0.028**	**1.37 (1.05 to 1.79)**	**0.019**
Region								
Rural	1.00 (reference)		1.00 (reference)		1.00 (reference)		1.00 (reference)	
Urban	1.06 (0.96 to 1.16)	0.246	1.05 (0.95 to 1.16)	0.354	1.07 (0.84 to 1.36)	0.604	1.02 (0.78 to 1.32)	0.910
Education								
College or higher	1.00 (reference)		1.00 (reference)		1.00 (reference)		1.00 (reference)	
High school or lower	**0.89 (0.79 to 0.99)**	**0.037**	0.91 (0.81 to 1.03)	0.132	0.88 (0.65 to 1.18)	0.383	0.96 (0.70 to 1.33)	0.811
Income								
Third or highest quartile	1.00 (reference)		1.00 (reference)		1.00 (reference)		1.00 (reference)	
Lowest or second quartile	**0.88 (0.81 to 0.95)**	**0.001**	**0.91 (0.83 to 0.98)**	**0.020**	**0.78 (0.62 to 0.98)**	**0.029**	0.86 (0.68 to 1.09)	0.223

*CI* confidence interval.

Numbers in bold indicate a significant difference (*P* < 0.05).

## Discussion

### Key findings

To the best of our knowledge, this is the first study to investigate the long-term trends and the potential impact of the COVID-19 pandemic on hypertension prevalence, awareness, treatment, and control, and its associated socioeconomic factors in South Korea (n = 108,687). We found that the hypertension prevalence increased throughout the pre-pandemic era; however, the increase in hypertension prevalence slowed during the pandemic era. The trends in hypertension awareness, treatment, control, and control among patients receiving treatment were similar. Young adults or men (19–59 years) were recognized as being more likely to benefit clinically after the pandemic era. Suggestions regarding the political implications of a clinical plan that considers hypertension prevalence, awareness, treatment, and control could be necessary based on our analysis of comparison to the COVID-19 pandemic era.

### Comparison with previous studies

Initially, investigations regarding the prevalence, awareness, treatment, control, and control among patients receiving treatment and its associated socioeconomic factors were not conducted in the specific context of the COVID-19 pandemic. Our research spans 24 years and aims to provide long-term insights. Prior studies have thoroughly investigated the relationship between hypertension and mortality rates during the COVID-19 pandemic or only the prevalence of hypertension. However, our focus had been on analyzing the patterns of health indicators associated with hypertension prevalence, awareness, treatment, control, and control among patients receiving treatment^19–22^. Our findings were consistent with previous research on the prevalence, awareness, treatment, control, and control among patients receiving treatment^8,23^.

Previous studies have identified various sociodemographic factors for hypertension, but none have examined these factors in the context of the COVID-19 pandemic^24,25^. A previous study reported that individuals > 60 years, men, and those with lower education and income levels were at a greater risk of hypertension^3,26^. However, it is imperative to note that these risk factors remained consistent during the pandemic era. Nevertheless, there may have been some differences in health indices associated with hypertension during this period.

### Possible mechanisms

Men and young adults may have had better hypertension control and treatment rates during this period for several reasons. First, the pandemic has led to an increased focus on public health and preventive measures, including managing chronic conditions such as hypertension^27^. The pandemic has heightened awareness of the significance of health. Individuals with chronic ailments, like hypertension, may be more inclined to manage their overall well-being diligently. As a result, there may be increased awareness among men and young adults regarding the importance of hypertension control and treatment, leading to better adherence to medication and lifestyle modifications. Second, telemedicine and remote monitoring have become more common during the pandemic^28^, making it easier for men and young adults to receive hypertension treatment and monitoring from the safety of their homes^29^. During the pandemic, healthcare organizations increased their use of in-person care, facilitating hypertension patients' access to healthcare and potentially improving blood pressure management. This may have led to increased access to healthcare and improved hypertension control and management^30^. Furthermore, the pandemic and lifestyle changes, such as reduced stress levels due to remote work or reduced commuting time, can positively impact hypertension control and treatment outcomes^31^. During the pandemic, individuals have been spending more time in their homes, potentially creating an environment that promotes a focus on health. The decrease in outdoor activities and increase in home-based routines may have contributed to this heightened awareness. For instance, implementing healthy meal choices or engaging in routine exercise may positively affect blood pressure management. Additionally, During the pandemic, there has been a significant improvement in the accessibility of health information through online platforms^32^. This has allowed patients to easily access vital information and gain a better understanding of their condition, which may have enabled them to adopt necessary lifestyle changes to manage their blood pressure effectively.

### Policy implications

Based on our study, the Korean clinical system effectively maintained health indices related to hypertension during the pandemic^33^. Further research should be conducted to better understand this clinical system. Additionally, this clinical system should be considered for treating morbidities other than hypertension. Behaviors of young adults and men aged 19–59 years should be analyzed to develop interventions that can be applied to all patients with hypertension as they showed higher performance regarding health indices related to hypertension. This can lead to significant improvements in the treatment and management of hypertension, although its incidence cannot be prevented. Based on our understanding of the existing clinical system, we must not ignore any potential weaknesses in the clinical infrastructure in preparation for future pandemic eras.

During the COVID-19 pandemic, we observed a deceleration in the rate of hypertension prevalence increase. This finding implies that preventative healthcare strategies and hypertension management policies can be successfully sustained even amidst a pandemic. Therefore, other nations can mitigate the negative impact of the COVID-19 crisis by implementing and promoting policies and initiatives aimed at enhancing hypertension prevention and management.

### Strengths and limitations

Our study has several strengths, including a large sample size, representative data from the Korean population, a comprehensive investigation of 24-year trends and risk factors in hypertension prevalence, awareness, treatment, and control, and a comparison between the pre- and COVID-19 pandemic eras. However, our study has some limitations. First, regardless of the length of treatment, the definition of hypertension treatment has changed over time, encompassing patients receiving antihypertensive medication. This inconsistency in the description of treatment across different periods in the KNHANES might have led to an overestimation of the rate of hypertension treatment from 1998 to 2005. Despite the potential limitations of our study, the overall impact on the trend is anticipated to be minimal, given the consistency of the definitions used in the latter periods and the absence of significant gaps in the existing literature. Furthermore, although our investigation centers on a pandemic with a relatively brief duration, it can serve as a viable surrogate for future research. Our research examined socioeconomic demographic changes over 24 years, including significant urbanization^34^. Thus, while our findings may not provide a definitive understanding of the trend, they have great academic value due to the duration of our study. To accurately reflect the actual trend, we treated non-responses to the questionnaire or “unknown” responses as negative responses, which had a negligible impact on the results and did not create any noticeable limitations.

## Conclusions

This study investigated long-term trends in the prevalence of hypertension and its related awareness, treatment, and control among Korean adults. We found that the prevalence of hypertension increased before the pandemic but slowed during the pandemic. The prevalence of hypertension was the highest in 2021, with rates of awareness, treatment, and control following a similar trend. This study suggests that young adults and men should receive clinical benefits after the pandemic. The absence of a reduction in the health indicators associated with hypertension during the pandemic implies that medical services for individuals with hypertension remain unaffected. Furthermore, the increase in health indices indicated that the Korean clinical system was effectively activated during the pandemic.

### Supplementary Information

Supplementary Tables.

## Data Availability

Data are available on reasonable request. Study protocol, statistical code: available from DKY (email: yonkkang@gmail.com). Data set: available from the Korea Disease Control and Prevention Agency (KDCA) through a data use agreement.

## References

[1] Worldwide trends in hypertension prevalence and progress in treatment and control from 1990 to 2019: A pooled analysis of 1201 population-representative studies with 104 million participants. *Lancet* **398**, 957–980 (2021). 10.1016/S0140-6736(21)01330-1 PMC8446938 34450083

[2] Kim HC (2022). Korea hypertension fact sheet 2021: Analysis of nationwide population-based data with special focus on hypertension in women. Clin. Hypertens..

[3] Zhang M (2023). Prevalence, awareness, treatment, and control of hypertension in China, 2004–18: Findings from six rounds of a national survey. BMJ.

[4] Cha Y, Jung W, Seo M, Rahmati M (2023). The emerging pandemic recent: SARS-CoV-2. Life Cycle.

[5] Shin H (2023). Estimated prevalence and trends in smoking among adolescents in South Korea, 2005–2021: A nationwide serial study. World journal of pediatrics : WJP.

[6] Kim SY, Yeniova AÖ (2022). Global, regional, and national incidence and mortality of COVID-19 in 237 countries and territories, January 2022: A systematic analysis for World Health Organization COVID-19 Dashboard. Life Cycle.

[7] Lee SW (2021). Association between mental illness and COVID-19 in South Korea: A post-hoc analysis. Lancet Psychiatry.

[8] Kang SH (2019). Prevalence, awareness, treatment, and control of hypertension in Korea. Sci. Rep..

[9] Jeon YW, Kim HC (2020). Factors associated with awareness, treatment, and control rate of hypertension among Korean young adults aged 30–49 years. Korean Circ. J..

[10] Kweon S (2014). Data resource profile: the Korea National Health and Nutrition Examination Survey (KNHANES). Int. J. Epidemiol..

[11] Koh HY (2019). Serum heavy metal levels are associated with asthma, allergic rhinitis, atopic dermatitis, allergic multimorbidity, and airflow obstruction. J. Allergy Clin. Immunol. Pract..

[12] Park S (2023). National trends in alcohol and substance use among adolescents from 2005 to 2021: A Korean serial cross-sectional study of one million adolescents. World J. Pediatr..

[13] Lee SW (2021). Proton pump inhibitors and the risk of severe COVID-19: a post-hoc analysis from the Korean nationwide cohort. Gut.

[14] Kim HL (2023). Standardized protocol of blood pressure measurement and quality control program for the Korea National Health and Nutrition Examination Survey. Clin. Hypertens..

[15] Yoo IK, Marshall DC, Cho JY, Yoo HW, Lee SW (2021). N-Nitrosodimethylamine-contaminated ranitidine and risk of cancer in South Korea: A nationwide cohort study. Life Cycle.

[16] Lee SW (2022). Regression analysis for continuous independent variables in medical research: Statistical standard and guideline of Life Cycle Committee. Life Cycle.

[17] Kwon R (2023). National trends in physical activity among adolescents in South Korea before and during the COVID-19 pandemic, 2009–2021. J. Med. Virol..

[18] Koo MJ (2022). National trends in the prevalence of allergic diseases among Korean adolescents before and during COVID-19, 2009–2021: A serial analysis of the national representative study. Allergy.

[19] Barrera FJ (2020). Prevalence of diabetes and hypertension and their associated risks for poor outcomes in Covid-19 patients. J. Endocr. Soc..

[20] Khairy Y, Naghibi D, Moosavi A, Sardareh M, Azami-Aghdash S (2022). Prevalence of hypertension and associated risks in hospitalized patients with COVID-19: A meta-analysis of meta-analyses with 1468 studies and 1,281,510 patients. Syst. Rev..

[21] Mistry SK (2022). Changes in prevalence and determinants of self-reported hypertension among Bangladeshi older adults during the COVID-19 pandemic. Int. J. Environ. Res. Public Health.

[22] Seclén SN, Nunez-Robles E, Yovera-Aldana M, Arias-Chumpitaz A (2020). Incidence of COVID-19 infection and prevalence of diabetes, obesity and hypertension according to altitude in Peruvian population. Diabetes Res. Clin. Pract..

[23] Seo E, Jung S, Lee H, Kim HC (2022). Sex-specific trends in the prevalence of hypertension and the number of people with hypertension: Analysis of the Korea National Health and Nutrition Examination Survey (KNHANES) 1998–2018. Korean Circ. J..

[24] Wyszyńska J, Łuszczki E, Sobek G, Mazur A, Dereń K (2023). Association and risk factors for hypertension and dyslipidemia in young adults from Poland. Int. J. Environ. Res. Public Health.

[25] Wang W, Sa R, Dang S, Qiu L, Liu F (2023). Prevalence, awareness, treatment, and control of hypertension and their risk factors in Shaanxi Province in 2004–18. Sci. Rep..

[26] Amini M (2022). Socioeconomic inequalities in prevalence, awareness, treatment and control of hypertension: Evidence from the PERSIAN cohort study. BMC Public Health.

[27] Coupet S, Nicolas G, Louder CN, Meyer M (2021). When public health messages become stressful: Managing chronic disease during COVID-19. Soc. Sci. Hum. Open.

[28] Yee V, Bajaj SS, Stanford FC (2022). Paradox of telemedicine: Building or neglecting trust and equity. Lancet Digit. Health.

[29] Fisk M, Livingstone A, Pit SW (2020). Telehealth in the context of COVID-19: Changing perspectives in Australia, the United Kingdom, and the United States. J. Med. Internet Res..

[30] Omboni S (2021). Telehealth at scale can improve chronic disease management in the community during a pandemic: An experience at the time of COVID-19. PLoS One.

[31] Wang C (2021). The impact of COVID-19 pandemic on physical and mental health of Asians: A study of seven middle-income countries in Asia. PLoS One.

[32] Dastani M, Atarodi A (2022). Health information technology during the COVID-19 epidemic: A review via text mining. Online J. Public Health Inform..

[33] Lee SW (2020). Nationwide results of COVID-19 contact tracing in South Korea: Individual participant data from an epidemiological survey. JMIR Med. Inform..

[34] Hong JW, Hong J, Kwon EE, Yoon DK (2019). Temporal dynamics of urban heat island correlated with the socio-economic development over the past half-century in Seoul, Korea. Environ. Pollut. (Barking, Essex: 1987).

