# Supplementary material for: Trends in hypertension prevalence, awareness, treatment, and control in South Korea, 1998–2021: a nationally representative serial study
Source: Sci Rep. 2023 Dec 8;13:21724. doi: 10.1038/s41598-023-49055-8 (PMC10709599; doi:10.1038/s41598-023-49055-8)
Supplement: Supplementary file 1 — Supplementary Tables. [file 41598_2023_49055_MOESM1_ESM.docx]

| **Supplementary Material** |
| --- |

Original Paper

**Trends in hypertension prevalence, awareness, treatment, and control in South Korea, 1998-2021: a nationally representative serial study**

Running title: **Hypertension and South Korea**

Myeongcheol Lee,^1,2^ Hojae Lee,^1,2^ Jaeyu Park,^1,2^ Hyeon Jin Kim,^1,2^ Rosie Kwon,^1,2^ Seung Won Lee,^3^ Sunyoung Kim,^4^ Ai Koyanagi,^5,6^ Lee Smith,^7^ Min Seo Kim,^8^ Guillaume Fond,^9^ Laurent Boyer,^9^ Masoud Rahmati,^10*^ Sang Youl Rhee,^2,11*^ Dong Keon Yon^1,2,12*^

**Supplementary Table 1.** Weighed trend of hypertension prevalence, awareness, treatment, control, and control among treated: weighted odds ratios with 95% CIs

|  |  | Weighted odds of before and during the pandemic, OR | | | | | | |
| --- | --- | --- | --- | --- | --- | --- | --- | --- |
|  |  | 2007 to 2009 versus 1998 to 2005 | 2010 to 2012 versus 2007 to 2009 | 2013 to 2015 versus 2010 to 2012 | 2016 to 2019 versus 2013 to 2015 | 2019 versus 1998 | 2021 versus 1998 | 2020 to 2021 versus 1998 to 2019 |
| Overall | Hypertension | 0.98 (0.90 to 1.06) | **1.11 (1.03 to 1.19)** | **0.87 (0.81 to 0.93)** | **1.19 (1.12 to 1.27)** | **1.17 (1.05 to 1.31)** | **1.17 (1.05 to 1.32)** | **1.12 (1.05 to 1.20)** |
|  | Awareness | **1.87 (1.65 to 2.11)** | 0.97 (0.86 to 1.08) | **1.19 (1.07 to 1.33)** | **1.30 (1.17 to 1.44)** | **8.18 (6.95 to 9.63)** | **10.08 (8.44 to 12.05)** | **1.62 (1.47 to 1.79)** |
|  | Treatment | **1.76 (1.56 to 1.99)** | 1.06 (0.95 to 1.19) | **1.24 (1.11 to 1.38)** | **1.26 (1.14 to 1.40)** | **8.24 (6.99 to 9.72)** | **10.25 (8.60 to 12.22)** | **1.63 (1.48 to 1.79)** |
|  | Control | **2.30 (1.98 to 2.67)** | 1.06 (0.94 to 1.20) | **1.40 (1.26 to 1.56)** | **1.21 (1.10 to 1.33)** | **19.27 (15.03 to 24.69)** | **26.89 (20.87 to 34.64)** | **1.70 (1.55 to 1.86)** |
|  | Control among treated | **2.01 (1.69 to 2.39)** | 1.03 (0.89 to 1.19) | **1.43 (1.25 to 1.64)** | 1.06 (0.93 to 1.20) | **8.16 (6.15 to 10.82)** | **11.84 (8.86 to 15.81)** | **1.48 (1.32 to 1.65)** |
| Sex | | | | | | | | |
| Men | Hypertension | 0.90 (0.81 to 1.00) | **1.12 (1.02 to 1.23)** | **0.85 (0.78 to 0.93)** | **1.19 (1.10 to 1.30)** | 1.04 (0.91 to 1.19) | 1.07 (0.93 to 1.23) | **1.12 (1.03 to 1.21)** |
|  | Awareness | **1.87 (1.59 to 2.20)** | 0.90 (0.78 to 1.04) | **1.31 (1.14 to 1.52)** | **1.42 (1.24 to 1.62)** | **10.48 (8.27 to 13.29)** | **12.97 (10.07 to 16.71)** | **1.82 (1.60 to 2.08)** |
|  | Treatment | **1.80 (1.53 to 2.12)** | 1.02 (0.88 to 1.18) | **1.35 (1.17 to 1.56)** | **1.39 (1.22 to 1.60)** | **10.92 (8.51 to 14.01)** | **13.52 (10.47 to 17.46)** | **1.82 (1.60 to 2.06)** |
|  | Control | **2.45 (1.96 to 3.04)** | 1.11 (0.95 to 1.30) | **1.45 (1.25 to 1.67)** | **1.40 (1.23 to 1.60)** | **28.08 (18.86 to 41.83)** | **36.17 (24.08 to 54.34)** | **1.86 (1.65 to 2.11)** |
|  | Control among treated | **2.08 (1.59 to 2.74)** | 1.19 (0.97 to 1.47) | **1.33 (1.10 to 1.61)** | **1.22 (1.02 to 1.46)** | **10.30 (6.47 to 16.40)** | **12.31 (7.68 to 19.73)** | **1.48 (1.25 to 1.75)** |
| Women | Hypertension | 1.03 (0.92 to 1.14) | 1.09 (1.00 to 1.19) | **0.89 (0.82 to 0.98)** | **1.19 (1.10 to 1.30)** | **1.31 (1.14 to 1.51)** | **1.27 (1.10 to 1.47)** | **1.12 (1.03 to 1.22)** |
|  | Awareness | **2.06 (1.75 to 2.41)** | 1.06 (0.91 to 1.24) | 1.04 (0.89 to 1.21) | **1.16 (1.01 to 1.33)** | **7.14 (5.73 to 8.91)** | **8.72 (6.79 to 11.19)** | **1.41 (1.23 to 1.63)** |
|  | Treatment | **1.92 (1.64 to 2.25)** | 1.14 (0.98 to 1.33) | 1.11 (0.96 to 1.28) | 1.12 (0.98 to 1.28) | **7.17 (5.80 to 8.86)** | **9.16 (7.13 to 11.76)** | **1.45 (1.26 to 1.66)** |
|  | Control | **2.35 (1.97 to 2.81)** | 1.02 (0.88 to 1.18) | **1.37 (1.19 to 1.57)** | 1.02 (0.90 to 1.15) | **14.57 (10.79 to 19.68)** | **21.89 (16.00 to 29.96)** | **1.55 (1.37 to 1.75)** |
|  | Control among treated | **1.99 (1.62 to 2.44)** | 0.92 (0.77 to 1.08) | **1.51 (1.27 to 1.79)** | 0.93 (0.79 to 1.08) | **6.95 (4.90 to 9.86)** | **11.34 (7.86 to 16.36)** | **1.46 (1.26 to 1.70)** |
| Region of residence | | | | | | | | |
| Urban | Hypertension | 1.01 (0.92 to 1.11) | **1.09 (1.00 to 1.18)** | **0.89 (0.83 to 0.97)** | **1.18 (1.10 to 1.27)** | **1.19 (1.05 to 1.34)** | 1.12 (0.99 to 1.27) | **1.10 (1.02 to 1.19)** |
|  | Awareness | **1.84 (1.59 to 2.13)** | 0.99 (0.86 to 1.13) | **1.25 (1.09 to 1.42)** | **1.21 (1.08 to 1.36)** | **8.59 (7.05 to 10.47)** | **10.48 (8.43 to 13.02)** | **1.63 (1.46 to 1.83)** |
|  | Treatment | **1.74 (1.51 to 2.02)** | 1.07 (0.93 to 1.22) | **1.32 (1.16 to 1.50)** | **1.18 (1.06 to 1.33)** | **8.08 (6.64 to 9.83)** | **10.29 (8.29 to 12.77)** | **1.66 (1.48 to 1.86)** |
|  | Control | **2.32 (1.94 to 2.77)** | 1.05 (0.90 to 1.21) | **1.48 (1.31 to 1.67)** | **1.14 (1.03 to 1.27)** | **18.19 (13.47 to 24.58)** | **25.88 (19.05 to 35.15)** | **1.73 (1.56 to 1.91)** |
|  | Control among treated | **2.06 (1.67 to 2.54)** | 1.00 (0.83 to 1.19) | **1.46 (1.25 to 1.71)** | 1.04 (0.90 to 1.19) | **7.92 (5.53 to 11.33)** | **11.33 (7.93 to 16.18)** | **1.48 (1.30 to 1.68)** |
| Rural | Hypertension | 0.92 (0.77 to 1.09) | 1.15 (0.98 to 1.35) | **0.83 (0.70 to 0.97)** | **1.32 (1.11 to 1.57)** | 1.26 (0.97 to 1.63) | **1.65 (1.30 to 2.09)** | **1.29 (1.09 to 1.52)** |
|  | Awareness | **2.07 (1.67 to 2.56)** | 0.88 (0.72 to 1.07) | 1.04 (0.83 to 1.31) | **1.78 (1.42 to 2.24)** | **7.84 (5.69 to 10.78)** | **9.50 (7.01 to 12.86)** | **1.61 (1.33 to 1.94)** |
|  | Treatment | **1.93 (1.55 to 2.40)** | 1.04 (0.85 to 1.26) | 1.04 (0.83 to 1.30) | **1.71 (1.36 to 2.15)** | **9.48 (6.76 to 13.30)** | **10.64 (7.98 to 14.18)** | **1.55 (1.30 to 1.84)** |
|  | Control | **2.33 (1.78 to 3.06)** | 1.10 (0.89 to 1.36) | 1.20 (0.95 to 1.52) | **1.51 (1.20 to 1.89)** | **23.17 (15.31 to 35.06)** | **30.02 (19.49 to 46.26)** | **1.62 (1.35 to 1.94)** |
|  | Control among treated | **1.88 (1.39 to 2.55)** | 1.13 (0.88 to 1.44) | **1.34 (1.02 to 1.76)** | 1.14 (0.88 to 1.48) | **9.39 (6.31 to 13.97)** | **13.20 (8.29 to 21.00)** | **1.46 (1.16 to 1.83)** |
| Age group | | | | | | | | |
| 19 to 59 | Hypertension | 1.01 (0.92 to 1.12) | 1.06 (0.97 to 1.16) | **0.83 (0.76 to 0.91)** | **1.14 (1.05 to 1.23)** | **0.84 (0.75 to 0.95)** | **0.83 (0.72 to 0.95)** | 1.00 (0.93 to 1.08) |
|  | Awareness | **1.70 (1.45 to 2.00)** | 0.86 (0.74 to 1.00) | 1.13 (0.97 to 1.32) | **1.27 (1.11 to 1.47)** | **5.50 (4.36 to 6.95)** | **7.07 (5.52 to 9.06)** | **1.56 (1.37 to 1.78)** |
|  | Treatment | **1.63 (1.38 to 1.92)** | 0.94 (0.81 to 1.10) | **1.22 (1.04 to 1.43)** | **1.25 (1.08 to 1.44)** | **5.91 (4.60 to 7.61)** | **8.20 (6.32 to 10.65)** | **1.59 (1.39 to 1.82)** |
|  | Control | **1.92 (1.56 to 2.38)** | 0.95 (0.80 to 1.13) | **1.41 (1.19 to 1.66)** | **1.28 (1.10 to 1.48)** | **13.21 (9.17 to 19.02)** | **21.68 (14.99 to 31.37)** | **1.86 (1.62 to 2.15)** |
|  | Control among treated | **1.60 (1.23 to 2.08)** | 0.99 (0.78 to 1.26) | **1.48 (1.17 to 1.88)** | 1.16 (0.93 to 1.45) | **6.72 (4.18 to 10.80)** | **12.94 (7.60 to 22.04)** | **1.83 (1.45 to 2.30)** |
| ≥60 | Hypertension | 0.98 (0.87 to 1.10) | **1.15 (1.04 to 1.26)** | **0.80 (0.73 to 0.88)** | **1.18 (1.08 to 1.29)** | **1.37 (1.16 to 1.61)** | **1.22 (1.04 to 1.44)** | 0.97 (0.90 to 1.06) |
|  | Awareness | **2.60 (2.19 to 3.08)** | 1.06 (0.90 to 1.24) | 1.17 (1.00 to 1.36) | **1.29 (1.12 to 1.49)** | **11.21 (9.07 to 13.86)** | **12.06 (9.49 to 15.32)** | **1.45 (1.26 to 1.66)** |
|  | Treatment | **2.39 (2.03 to 2.81)** | **1.19 (1.02 to 1.38)** | **1.17 (1.01 to 1.36)** | **1.24 (1.08 to 1.42)** | **10.05 (8.23 to 12.27)** | **10.75 (8.60 to 13.43)** | **1.43 (1.26 to 1.62)** |
|  | Control | **2.95 (2.44 to 3.57)** | 1.12 (0.97 to 1.30) | **1.35 (1.18 to 1.54)** | 1.11 (0.99 to 1.24) | **23.45 (16.84 to 32.66)** | **27.55 (19.84 to 38.26)** | **1.43 (1.28 to 1.60)** |
|  | Control among treated | **2.34 (1.90 to 2.89)** | 1.05 (0.89 to 1.24) | **1.40 (1.20 to 1.64)** | 1.00 (0.87 to 1.16) | **10.82 (7.55 to 15.50)** | **13.89 (9.69 to 19.90)** | **1.33 (1.17 to 1.51)** |
| Educational background | | | | | | | | |
| High school or lower | Hypertension | 0.94 (0.86 to 1.03) | **1.13 (1.05 to 1.22)** | **0.87 (0.80 to 0.94)** | **1.30 (1.21 to 1.40)** | **1.45 (1.28 to 1.65)** | **1.46 (1.29 to 1.65)** | **1.21 (1.12 to 1.30)** |
|  | Awareness | **1.84 (1.61 to 2.09)** | 1.02 (0.91 to 1.15) | **1.18 (1.04 to 1.33)** | **1.41 (1.26 to 1.58)** | **9.02 (7.60 to 10.70)** | **11.28 (9.35 to 13.62)** | **1.74 (1.55 to 1.95)** |
|  | Treatment | **1.74 (1.53 to 1.97)** | **1.16 (1.03 to 1.30)** | **1.21 (1.08 to 1.36)** | **1.36 (1.21 to 1.52)** | **9.20 (7.75 to 10.92)** | **11.82 (9.84 to 14.20)** | **1.76 (1.58 to 1.96)** |
|  | Control | **2.26 (1.94 to 2.64)** | **1.14 (1.01 to 1.29)** | **1.36 (1.21 to 1.53)** | **1.22 (1.10 to 1.36)** | **20.75 (16.24 to 26.51)** | **29.23 (22.76 to 37.54)** | **1.75 (1.59 to 1.93)** |
|  | Control among treated | **2.00 (1.68 to 2.40)** | 1.06 (0.91 to 1.24) | **1.39 (1.21 to 1.61)** | 1.01 (0.89 to 1.16) | **8.32 (6.28 to 11.01)** | **11.91 (8.95 to 15.86)** | **1.46 (1.29 to 1.65)** |
| College or higher | Hypertension | 1.07 (0.92 to 1.26) | 1.09 (0.94 to 1.27) | 0.91 (0.79 to 1.04) | **1.22 (1.09 to 1.37)** | 1.11 (0.92 to 1.33) | 1.10 (0.90 to 1.34) | **1.15 (1.04 to 1.28)** |
|  | Awareness | **2.22 (1.65 to 2.98)** | **0.77 (0.60 to 0.99)** | **1.37 (1.07 to 1.74)** | **1.27 (1.03 to 1.58)** | **11.00 (6.95 to 17.40)** | **12.49 (7.79 to 20.02)** | **1.66 (1.39 to 1.98)** |
|  | Treatment | **2.07 (1.52 to 2.82)** | **0.75 (0.58 to .98)** | **1.54 (1.21 to 1.96)** | **1.30 (1.06 to 1.60)** | **8.83 (5.54 to 14.07)** | **10.80 (6.67 to 17.51)** | **1.66 (1.38 to 1.99)** |
|  | Control | **2.72 (1.79 to 4.14)** | **0.70 (0.52 to 0.96)** | **1.86 (1.45 to 2.40)** | **1.37 (1.11 to 1.70)** | **16.20 (7.79 to 33.65)** | **22.05 (10.60 to 45.87)** | **1.78 (1.47 to 2.15)** |
|  | Control among treated | **2.07 (1.23 to 3.49)** | 0.79 (0.52 to 1.19) | **1.77 (1.23 to 2.56)** | 1.29 (0.94 to 1.76) | **5.92 (2.49 to 14.08)** | **8.84 (3.61 to 21.64)** | **1.52 (1.13 to 2.04)** |
| Income | | | | | | | | |
| Lowest or second quartile | Hypertension | 1.03 (0.93 to 1.15) | 1.06 (0.96 to 1.16) | 0.93 (0.85 to 1.02) | **1.24 (1.14 to 1.36)** | **1.42 (1.23 to 1.65)** | **1.46 (1.25 to 1.70)** | **1.23 (1.12 to 1.35)** |
|  | Awareness | **1.91 (1.64 to 2.22)** | 1.04 (0.90 to 1.21) | **1.24 (1.07 to 1.44)** | **1.36 (1.18 to 1.56)** | **9.09 (7.31 to 11.30)** | **13.12 (10.30 to 16.71)** | **1.79 (1.56 to 2.06)** |
|  | Treatment | **1.79 (1.53 to 2.08)** | **1.16 (1.00 to 1.34)** | **1.29 (1.12 to 1.49)** | **1.26 (1.10 to 1.45)** | **9.06 (7.25 to 11.33)** | **13.17 (10.40 to 16.69)** | **1.80 (1.58 to 2.05)** |
|  | Control | **2.33 (1.93 to 2.80)** | **1.16 (1.00 to 1.34)** | **1.41 (1.23 to 1.62)** | **1.17 (1.03 to 1.32)** | **23.65 (17.09 to 32.72)** | **35.78 (25.67 to 49.86)** | **1.70 (1.51 to 1.91)** |
|  | Control among treated | **2.02 (1.64 to 2.50)** | 1.09 (0.92 to 1.29) | **1.38 (1.17 to 1.63)** | 1.03 (0.88 to 1.19) | **9.82 (6.87 to 14.05)** | **13.82 (9.56 to 19.99)** | **1.37 (1.19 to 1.59)** |
| Third or highest quartile | Hypertension | 0.99 (0.89 to 1.10) | **1.14 (1.03 to 1.25)** | **0.85 (0.77 to 0.93)** | **1.16 (1.07 to 1.26)** | 1.00 (0.87 to 1.15) | 1.05 (0.92 to 1.19) | **1.11 (1.03 to 1.20)** |
|  | Awareness | **1.79 (1.49 to 2.14)** | 0.92 (0.79 to 1.08) | 1.16 (0.99 to 1.35) | **1.26 (1.10 to 1.45)** | **7.44 (5.82 to 9.52)** | **8.12 (6.26 to 10.52)** | **1.58 (1.39 to 1.80)** |
|  | Treatment | **1.71 (1.43 to 2.06)** | 1.01 (0.86 to 1.19) | **1.22 (1.04 to 1.42)** | **1.27 (1.11 to 1.46)** | **7.34 (5.77 to 9.34)** | **8.38 (6.49 to 10.81)** | **1.59 (1.39 to 1.81)** |
|  | Control | **2.16 (1.72 to 2.70)** | 0.99 (0.83 to 1.19) | **1.41 (1.22 to 1.64)** | **1.25 (1.10 to 1.43)** | **15.92 (11.19 to 22.66)** | **20.56 (14.28 to 29.60)** | **1.74 (1.53 to 1.98)** |
|  | Control among treated | **1.87 (1.42 to 2.45)** | 0.97 (0.77 to 1.23) | **1.51 (1.21 to 1.88)** | 1.10 (0.91 to 1.34) | **7.46 (4.90 to 11.37)** | **10.87 (6.97 to 16.93)** | **1.59 (1.33 to 1.92)** |

CI, confidence interval.

Numbers in bold indicate a significant difference (*P* < 0.05).

**Supplementary Table 2.** Trends in prevalence, awareness, treatment, control, and control among treated of hypertension in urban and rural areas, weighted % (95% CI)

| Sex | Prevalence | Residence | 1998 to 2005 | 2007 to 2009 | 2010 to 2012 | 2013 to 2015 | 2016 to 2019 | 2020 | 2021 |
| --- | --- | --- | --- | --- | --- | --- | --- | --- | --- |
| Men | Hypertension | Urban | 29.45 (27.53 to 31.37) | 27.31 (25.80 to 28.82) | 29.42 (27.93 to 30.91) | 26.34 (24.95 to 27.73) | 29.67 (28.54 to 30.80) | 32.38 (29.95 to 34.82) | 27.70 (25.32 to 30.08) |
|  |  | Rural | 31.86 (28.46 to 35.27) | 30.23 (27.09 to 33.37) | 33.47 (30.08 to 36.86) | 29.44 (26.19 to 32.68) | 35.32 (32.36 to 38.27) | 36.72 (31.52 to 41.93) | 41.70 (36.64 to 46.76) |
|  | Awareness | Urban | 33.13 (29.95 to 36.32) | 48.21 (45.27 to 51.16) | 46.82 (43.99 to 49.65) | 54.08 (51.06 to 57.10) | 60.67 (58.48 to 62.86) | 64.58 (60.35 to 68.82) | 68.51 (63.83 to 73.20) |
|  |  | Rural | 40.26 (34.70 to 45.81) | 56.67 (52.04 to 61.29) | 49.55 (44.55 to 54.54) | 54.94 (49.34 to 60.53) | 71.83 (67.65 to 76.01) | 65.37 (58.48 to 72.25) | 76.20 (69.96 to 82.45) |
|  | Treatment | Urban | 27.58 (24.57 to 30.58) | 27.58 (24.57 to 30.58) | 27.58 (24.57 to 30.58) | 27.58 (24.57 to 30.58) | 27.58 (24.57 to 30.58) | 27.58 (24.57 to 30.58) | 27.58 (24.57 to 30.58) |
|  |  | Rural | 34.55 (29.46 to 39.64) | 34.55 (29.46 to 39.64) | 34.55 (29.46 to 39.64) | 34.55 (29.46 to 39.64) | 34.55 (29.46 to 39.64) | 34.55 (29.46 to 39.64) | 34.55 (29.46 to 39.64) |
|  | Control | Urban | 12.23 (9.87 to 14.59) | 25.43 (22.67 to 28.18) | 27.17 (24.91 to 29.43) | 36.48 (33.85 to 39.11) | 42.56 (40.31 to 44.81) | 44.03 (39.76 to 48.31) | 51.61 (46.73 to 56.50) |
|  |  | Rural | 14.24 (10.44 to 18.03) | 29.32 (25.01 to 33.62) | 32.10 (27.20 to 36.99) | 35.55 (30.20 to 40.90) | 53.07 (48.60 to 57.55) | 46.47 (39.33 to 53.62) | 56.52 (48.65 to 64.40) |
|  | Control among treated | Urban | 44.35 (37.77 to 50.93) | 44.35 (37.77 to 50.93) | 44.35 (37.77 to 50.93) | 44.35 (37.77 to 50.93) | 44.35 (37.77 to 50.93) | 44.35 (37.77 to 50.93) | 44.35 (37.77 to 50.93) |
|  |  | Rural | 41.20 (32.15 to 50.26) | 41.20 (32.15 to 50.26) | 41.20 (32.15 to 50.26) | 41.20 (32.15 to 50.26) | 41.20 (32.15 to 50.26) | 41.20 (32.15 to 50.26) | 41.20 (32.15 to 50.26) |
|  | | | | | | | | | |
| Women | Hypertension | Urban | 18.98 (17.56 to 20.40) | 20.45 (19.22 to 21.68) | 21.59 (20.38 to 22.80) | 20.45 (19.28 to 21.61) | 23.25 (22.17 to 24.33) | 23.91 (21.62 to 26.20) | 23.13 (20.98 to 25.27) |
|  |  | Rural | 32.59 (29.25 to 35.94) | 30.51 (27.69 to 33.33) | 33.40 (30.16 to 36.63) | 29.16 (26.06 to 32.27) | 35.44 (32.01 to 38.88) | 30.85 (24.60 to 37.09) | 42.27 (36.12 to 48.42) |
|  | Awareness | Urban | 52.70 (49.20 to 56.19) | 68.44 (65.52 to 71.37) | 69.69 (66.91 to 72.48) | 71.75 (69.29 to 74.20) | 73.53 (71.56 to 75.49) | 74.65 (71.04 to 78.26) | 78.72 (74.54 to 82.90) |
|  |  | Rural | 52.88 (47.85 to 57.90) | 73.13 (69.93 to 76.33) | 74.21 (70.80 to 77.61) | 71.39 (67.14 to 75.65) | 78.43 (75.19 to 81.68) | 80.44 (74.80 to 86.09) | 77.48 (72.10 to 82.87) |
|  | Treatment | Urban | 48.47 (45.03 to 51.90) | 48.47 (45.03 to 51.90) | 48.47 (45.03 to 51.90) | 48.47 (45.03 to 51.90) | 48.47 (45.03 to 51.90) | 48.47 (45.03 to 51.90) | 48.47 (45.03 to 51.90) |
|  |  | Rural | 50.04 (44.75 to 55.32) | 50.04 (44.75 to 55.32) | 50.04 (44.75 to 55.32) | 50.04 (44.75 to 55.32) | 50.04 (44.75 to 55.32) | 50.04 (44.75 to 55.32) | 50.04 (44.75 to 55.32) |
|  | Control | Urban | 22.87 (19.98 to 25.76) | 41.06 (37.92 to 44.20) | 41.11 (38.32 to 43.91) | 49.67 (46.93 to 52.42) | 49.63 (47.57 to 51.69) | 50.84 (46.29 to 55.40) | 60.87 (55.90 to 65.84) |
|  |  | Rural | 24.40 (19.36 to 29.45) | 43.83 (39.59 to 48.08) | 45.43 (40.60 to 50.26) | 51.35 (46.80 to 55.91) | 53.78 (49.29 to 58.27) | 53.30 (45.82 to 60.77) | 56.28 (49.45 to 63.11) |
|  | Control among treated | Urban | 47.18 (42.38 to 51.98) | 47.18 (42.38 to 51.98) | 47.18 (42.38 to 51.98) | 47.18 (42.38 to 51.98) | 47.18 (42.38 to 51.98) | 47.18 (42.38 to 51.98) | 47.18 (42.38 to 51.98) |
|  |  | Rural | 48.77 (40.83 to 56.72) | 48.77 (40.83 to 56.72) | 48.77 (40.83 to 56.72) | 48.77 (40.83 to 56.72) | 48.77 (40.83 to 56.72) | 48.77 (40.83 to 56.72) | 48.77 (40.83 to 56.72) |

CI, confidence interval.

**Supplementary Table 3.** Trends in prevalence, awareness, treatment, control, and control among treated of hypertension by education, weighted % (95% CI)

| Sex | Prevalence | Education | 1998 to 2005 | 2007 to 2009 | 2010 to 2012 | 2013 to 2015 | 2016 to 2019 | 2020 | 2021 |
| --- | --- | --- | --- | --- | --- | --- | --- | --- | --- |
| Men | Prevalence | Elementary school or lower | 44.27 (40.09 to 48.45) | 47.05 (43.72 to 50.38) | 43.25 (39.77 to 46.74) | 34.18 (31.45 to 36.92) | 44.97 (42.26 to 47.68) | 53.66 (48.11 to 59.21) | 52.66 (47.23 to 58.08) |
|  |  | Middle school | 43.33 (38.81 to 47.85) | 38.61 (34.60 to 42.61) | 42.40 (38.59 to 46.21) | 40.13 (36.09 to 44.17) | 49.37 (45.71 to 53.03) | 50.81 (43.40 to 58.22) | 48.17 (41.18 to 55.17) |
|  |  | High school | 28.92 (26.35 to 31.50) | 23.16 (21.30 to 25.01) | 27.12 (25.02 to 29.23) | 23.69 (21.78 to 25.60) | 27.43 (25.72 to 29.15) | 29.53 (25.91 to 33.16) | 28.24 (24.36 to 32.12) |
|  |  | University or higher | 21.66 (19.41 to 23.91) | 22.63 (20.48 to 24.79) | 24.51 (22.43 to 26.59) | 22.85 (20.84 to 24.85) | 24.91 (23.40 to 26.42) | 26.88 (23.77 to 29.99) | 22.25 (19.30 to 25.21) |
|  | Awareness | Elementary school or lower | 45.04 (39.74 to 50.34) | 60.32 (55.92 to 64.72) | 57.77 (53.67 to 61.88) | 55.71 (51.19 to 60.23) | 76.12 (72.84 to 79.40) | 75.07 (66.71 to 83.44) | 77.91 (70.93 to 84.90) |
|  |  | Middle school | 38.25 (32.27 to 44.22) | 57.76 (51.88 to 63.65) | 60.56 (54.60 to 66.52) | 65.02 (58.88 to 71.17) | 77.19 (72.64 to 81.75) | 82.32 (73.43 to 91.22) | 78.70 (69.06 to 88.33) |
|  |  | High school | 33.79 (29.03 to 38.54) | 44.89 (40.72 to 49.05) | 43.35 (39.30 to 47.40) | 57.93 (53.53 to 62.33) | 60.60 (57.12 to 64.09) | 61.50 (55.41 to 67.58) | 72.28 (65.69 to 78.87) |
|  |  | University or higher | 26.25 (21.69 to 30.80) | 43.65 (38.96 to 48.33) | 37.97 (33.58 to 42.37) | 44.65 (39.58 to 49.73) | 51.32 (47.97 to 54.67) | 56.08 (48.95 to 63.21) | 60.27 (52.76 to 67.78) |
|  | Treatment | Elementary school or lower | 38.19 (32.80 to 43.59) | 54.22 (49.75 to 58.68) | 55.29 (51.13 to 59.44) | 53.40 (48.90 to 57.89) | 72.46 (69.01 to 75.92) | 72.16 (63.85 to 80.47) | 75.34 (68.44 to 82.23) |
|  |  | Middle school | 30.03 (24.44 to 35.63) | 50.42 (44.29 to 56.55) | 56.32 (50.25 to 62.39) | 59.72 (53.29 to 66.15) | 74.13 (69.40 to 78.87) | 77.43 (68.04 to 86.81) | 74.24 (64.26 to 84.22) |
|  |  | High school | 29.19 (24.78 to 33.61) | 35.67 (31.63 to 39.70) | 39.14 (35.22 to 43.07) | 54.81 (50.29 to 59.32) | 56.37 (52.85 to 59.89) | 59.01 (53.29 to 64.72) | 67.03 (60.08 to 73.97) |
|  |  | University or higher | 21.97 (17.75 to 26.19) | 37.57 (32.77 to 42.38) | 31.87 (27.72 to 36.02) | 39.78 (35.06 to 44.49) | 47.35 (43.92 to 50.79) | 48.33 (41.43 to 55.24) | 56.84 (49.21 to 64.47) |
|  | Control | Elementary school or lower | 16.58 (12.50 to 20.66) | 32.00 (27.65 to 36.35) | 39.98 (35.55 to 44.41) | 36.16 (32.26 to 40.05) | 56.96 (53.19 to 60.73) | 55.21 (47.19 to 63.23) | 57.27 (50.53 to 64.01) |
|  |  | Middle school | 13.13 (8.91 to 17.36) | 30.46 (24.99 to 35.93) | 38.37 (32.61 to 44.14) | 45.87 (39.58 to 52.16) | 54.35 (49.43 to 59.28) | 59.93 (48.84 to 71.02) | 56.38 (44.82 to 67.93) |
|  |  | High school | 12.18 (9.37 to 15.00) | 22.10 (18.78 to 25.41) | 24.66 (21.31 to 28.02) | 40.93 (36.82 to 45.03) | 42.36 (38.86 to 45.86) | 42.72 (36.30 to 49.14) | 53.77 (46.18 to 61.36) |
|  |  | University or higher | 10.13 (6.78 to 13.48) | 24.45 (19.83 to 29.07) | 18.58 (15.57 to 21.59) | 27.59 (23.38 to 31.79) | 35.34 (32.10 to 38.57) | 34.69 (28.44 to 40.95) | 47.42 (39.82 to 55.01) |
|  | Control among treated | Elementary school or lower | 16.58 (12.50 to 20.66) | 32.00 (27.65 to 36.35) | 39.98 (35.55 to 44.41) | 36.16 (32.26 to 40.05) | 56.96 (53.19 to 60.73) | 55.21 (47.19 to 63.23) | 57.27 (50.53 to 64.01) |
|  |  | Middle school | 13.13 (8.91 to 17.36) | 30.46 (24.99 to 35.93) | 38.37 (32.61 to 44.14) | 45.87 (39.58 to 52.16) | 54.35 (49.43 to 59.28) | 59.93 (48.84 to 71.02) | 56.38 (44.82 to 67.93) |
|  |  | High school | 12.18 (9.37 to 15.00) | 22.10 (18.78 to 25.41) | 24.66 (21.31 to 28.02) | 40.93 (36.82 to 45.03) | 42.36 (38.86 to 45.86) | 42.72 (36.30 to 49.14) | 53.77 (46.18 to 61.36) |
|  |  | University or higher | 10.13 (6.78 to 13.48) | 24.45 (19.83 to 29.07) | 18.58 (15.57 to 21.59) | 27.59 (23.38 to 31.79) | 35.34 (32.10 to 38.57) | 34.69 (28.44 to 40.95) | 47.42 (39.82 to 55.01) |
|  | | | | | | | | | |
| Women | Prevalence | Elementary school or lower | 48.10 (45.63 to 50.58) | 50.94 (48.82 to 53.07) | 52.61 (50.41 to 54.82) | 44.61 (42.39 to 46.82) | 55.25 (53.26 to 57.25) | 53.73 (49.58 to 57.88) | 55.29 (51.11 to 59.47) |
|  |  | Middle school | 30.21 (26.97 to 33.44) | 28.29 (25.16 to 31.41) | 34.16 (30.53 to 37.80) | 34.44 (31.01 to 37.87) | 37.59 (34.49 to 40.69) | 43.42 (37.29 to 49.54) | 51.47 (44.82 to 58.12) |
|  |  | High school | 10.40 (8.97 to 11.83) | 11.95 (10.63 to 13.26) | 12.50 (11.27 to 13.74) | 13.18 (11.70 to 14.67) | 18.42 (17.04 to 19.81) | 17.89 (15.26 to 20.53) | 19.44 (16.78 to 22.09) |
|  |  | University or higher | 4.35 (3.26 to 5.43) | 4.46 (3.45 to 5.48) | 5.61 (4.64 to 6.57) | 5.62 (4.75 to 6.50) | 8.68 (7.77 to 9.59) | 10.53 (8.44 to 12.62) | 10.58 (8.38 to 12.78) |
|  | Awareness | Elementary school or lower | 57.27 (53.83 to 60.71) | 76.83 (74.44 to 79.23) | 77.29 (74.68 to 79.90) | 76.25 (73.68 to 78.81) | 83.19 (81.29 to 85.10) | 84.89 (81.26 to 88.51) | 87.58 (83.60 to 91.55) |
|  |  | Middle school | 59.27 (52.95 to 65.59) | 65.49 (59.05 to 71.92) | 68.97 (63.84 to 74.09) | 73.13 (67.20 to 79.07) | 74.66 (70.30 to 79.01) | 77.83 (69.52 to 86.14) | 76.70 (69.27 to 84.14) |
|  |  | High school | 35.59 (28.41 to 42.77) | 54.12 (48.87 to 59.38) | 60.90 (55.86 to 65.94) | 63.45 (58.43 to 68.48) | 65.40 (61.69 to 69.12) | 70.84 (63.04 to 78.65) | 72.67 (65.47 to 79.88) |
|  |  | University or higher | 31.30 (19.16 to 43.44) | 53.90 (42.09 to 65.70) | 42.92 (33.78 to 52.06) | 54.29 (45.87 to 62.71) | 56.18 (50.66 to 61.69) | 53.89 (44.90 to 62.88) | 63.64 (52.84 to 74.44) |
|  | Treatment | Elementary school or lower | 52.87 (49.22 to 56.51) | 71.85 (69.27 to 74.42) | 75.47 (72.83 to 78.12) | 75.25 (72.69 to 77.81) | 81.26 (79.28 to 83.24) | 83.27 (79.14 to 87.40) | 86.04 (81.86 to 90.21) |
|  |  | Middle school | 53.05 (46.73 to 59.36) | 61.20 (54.45 to 67.95) | 65.36 (60.02 to 70.70) | 69.99 (63.98 to 76.01) | 71.58 (67.12 to 76.05) | 76.32 (67.89 to 84.75) | 74.69 (66.75 to 82.63) |
|  |  | High school | 35.43 (28.43 to 42.43) | 50.15 (44.69 to 55.61) | 55.62 (50.47 to 60.76) | 60.63 (55.47 to 65.78) | 63.09 (59.31 to 66.87) | 66.24 (58.20 to 74.27) | 71.72 (64.51 to 78.93) |
|  |  | University or higher | 31.53 (20.33 to 42.72) | 45.49 (33.20 to 57.78) | 33.56 (25.08 to 42.05) | 53.01 (44.54 to 61.49) | 53.09 (47.68 to 58.50) | 50.76 (41.67 to 59.84) | 61.14 (50.05 to 72.24) |
|  | Control | Elementary school or lower | 23.35 (20.31 to 26.40) | 44.86 (41.90 to 47.82) | 47.03 (43.90 to 50.16) | 52.56 (49.60 to 55.51) | 54.80 (52.22 to 57.38) | 56.99 (51.74 to 62.24) | 62.18 (56.70 to 67.66) |
|  |  | Middle school | 30.04 (24.15 to 35.93) | 41.35 (34.55 to 48.14) | 42.89 (36.87 to 48.91) | 49.37 (42.92 to 55.82) | 50.25 (45.46 to 55.04) | 54.71 (44.53 to 64.88) | 59.57 (50.96 to 68.18) |
|  |  | High school | 19.36 (14.22 to 24.49) | 36.60 (31.33 to 41.87) | 34.14 (29.04 to 39.25) | 46.53 (41.29 to 51.78) | 45.54 (41.46 to 49.61) | 49.42 (40.69 to 58.16) | 61.01 (52.65 to 69.36) |
|  |  | University or higher | 14.12 (6.01 to 22.23) | 25.39 (15.63 to 35.16) | 19.07 (12.65 to 25.49) | 40.74 (32.56 to 48.92) | 42.49 (37.08 to 47.90) | 34.11 (24.87 to 43.35) | 50.94 (39.31 to 62.57) |
|  | Control among treated | Elementary school or lower | 23.35 (20.31 to 26.40) | 44.86 (41.90 to 47.82) | 47.03 (43.90 to 50.16) | 52.56 (49.60 to 55.51) | 54.80 (52.22 to 57.38) | 56.99 (51.74 to 62.24) | 62.18 (56.70 to 67.66) |
|  |  | Middle school | 30.04 (24.15 to 35.93) | 41.35 (34.55 to 48.14) | 42.89 (36.87 to 48.91) | 49.37 (42.92 to 55.82) | 50.25 (45.46 to 55.04) | 54.71 (44.53 to 64.88) | 59.57 (50.96 to 68.18) |
|  |  | High school | 19.36 (14.22 to 24.49) | 36.60 (31.33 to 41.87) | 34.14 (29.04 to 39.25) | 46.53 (41.29 to 51.78) | 45.54 (41.46 to 49.61) | 49.42 (40.69 to 58.16) | 61.01 (52.65 to 69.36) |
|  |  | University or higher | 14.12 (6.01 to 22.23) | 25.39 (15.63 to 35.16) | 19.07 (12.65 to 25.49) | 40.74 (32.56 to 48.92) | 42.49 (37.08 to 47.90) | 34.11 (24.87 to 43.35) | 50.94 (39.31 to 62.57) |

CI, confidence interval.

**Supplementary Table 4.** Trends in prevalence, awareness, treatment, control, and control among treated of hypertension by age group, weighted % (95% CI)

| Sex | Prevalence | Age | 1998 to 2005 | 2007 to 2009 | 2010 to 2012 | 2013 to 2015 | 2016 to 2019 | 2020 | 2021 |
| --- | --- | --- | --- | --- | --- | --- | --- | --- | --- |
| Men | Prevalence | 19 to 29 | 9.18 (6.88 to 11.49) | 7.70 (5.86 to 9.55) | 11.24 (8.82 to 13.66) | 5.23 (3.74 to 6.73) | 7.31 (5.64 to 8.98) | 7.43 (4.40 to 10.45) | 3.86 (1.56 to 6.15) |
|  |  | 30 to 39 | 18.71 (16.29 to 21.14) | 17.42 (15.07 to 19.78) | 17.02 (14.78 to 19.26) | 15.27 (13.08 to 17.47) | 16.81 (14.84 to 18.78) | 17.62 (13.41 to 21.83) | 17.29 (12.25 to 22.33) |
|  |  | 40 to 49 | 29.72 (26.76 to 32.69) | 28.85 (26.23 to 31.46) | 30.75 (27.90 to 33.60) | 27.15 (24.47 to 29.83) | 28.33 (26.21 to 30.44) | 31.52 (26.50 to 36.55) | 25.63 (21.32 to 29.93) |
|  |  | 50 to 59 | 43.99 (40.44 to 47.53) | 42.84 (39.63 to 46.04) | 41.82 (38.73 to 44.92) | 36.86 (34.23 to 39.50) | 39.36 (36.99 to 41.74) | 45.41 (40.94 to 49.88) | 35.84 (31.03 to 40.65) |
|  |  | 60 to 69 | 53.95 (49.72 to 58.17) | 49.82 (46.49 to 53.16) | 54.48 (51.23 to 57.74) | 46.58 (43.30 to 49.87) | 50.74 (48.19 to 53.28) | 50.45 (45.16 to 55.75) | 50.58 (45.21 to 55.94) |
|  |  | 70 to 79 | 51.69 (45.28 to 58.09) | 54.63 (50.44 to 58.81) | 55.60 (51.85 to 59.35) | 56.52 (52.76 to 60.28) | 61.25 (58.53 to 63.97) | 63.51 (58.08 to 68.94) | 61.41 (55.31 to 67.52) |
|  |  | ≥80 | 46.42 (31.75 to 61.10) | 62.43 (54.05 to 70.81) | 53.43 (45.45 to 61.41) | 48.17 (40.44 to 55.91) | 66.24 (61.24 to 71.24) | 47.76 (39.56 to 55.96) | 61.34 (53.10 to 69.58) |
|  | Awareness | 19 to 29 | 4.76 (0.00 to 9.88) | 16.37 (6.29 to 26.45) | 6.21 (1.11 to 11.31) | 8.56 (0.12 to 17.00) | 17.31 (8.51 to 26.12) | 10.93 (0.00 to 23.82) | 17.81 (0.00 to 43.34) |
|  |  | 30 to 39 | 10.25 (5.26 to 15.25) | 19.20 (13.80 to 24.60) | 14.70 (9.74 to 19.65) | 18.14 (11.85 to 24.42) | 18.33 (13.04 to 23.61) | 16.61 (6.09 to 27.13) | 40.71 (24.82 to 56.60) |
|  |  | 40 to 49 | 27.48 (22.38 to 32.58) | 37.33 (32.15 to 42.50) | 31.03 (26.02 to 36.04) | 36.85 (31.08 to 42.63) | 44.34 (39.56 to 49.12) | 55.41 (45.94 to 64.89) | 53.40 (42.63 to 64.17) |
|  |  | 50 to 59 | 44.96 (39.55 to 50.38) | 58.70 (53.74 to 63.65) | 55.73 (51.03 to 60.43) | 55.17 (50.34 to 59.99) | 67.24 (63.48 to 71.01) | 65.13 (58.34 to 71.93) | 75.71 (68.32 to 83.10) |
|  |  | 60 to 69 | 48.10 (42.64 to 53.56) | 75.22 (70.80 to 79.64) | 71.09 (66.98 to 75.20) | 78.72 (75.36 to 82.08) | 82.16 (79.46 to 84.85) | 85.00 (78.68 to 91.31) | 74.59 (68.10 to 81.09) |
|  |  | 70 to 79 | 54.61 (46.66 to 62.56) | 71.09 (66.51 to 75.67) | 79.19 (75.21 to 83.17) | 82.34 (78.78 to 85.90) | 87.58 (85.17 to 90.00) | 86.61 (81.62 to 91.61) | 91.95 (88.31 to 95.59) |
|  |  | ≥80 | 36.21 (15.88 to 56.55) | 70.40 (60.35 to 80.46) | 73.55 (63.03 to 84.08) | 75.27 (66.65 to 83.88) | 85.70 (81.71 to 89.68) | 79.66 (67.13 to 92.19) | 87.76 (80.84 to 94.68) |
|  | Treatment | 19 to 29 | 3.25 (0.00 to 7.64) | 7.49 (1.02 to 13.97) | 1.32 (0.00 to 3.19) | 1.72 (0.00 to 5.08) | 8.75 (1.63 to 15.87) | 3.09 (0.00 to 9.10) | 17.81 (0.00 to 43.17) |
|  |  | 30 to 39 | 7.35 (2.94 to 11.75) | 10.46 (6.43 to 14.49) | 8.46 (4.69 to 12.24) | 13.14 (7.89 to 18.39) | 14.72 (9.90 to 19.54) | 11.05 (2.43 to 19.67) | 36.18 (20.69 to 51.67) |
|  |  | 40 to 49 | 19.73 (15.25 to 24.20) | 26.21 (21.21 to 31.21) | 25.67 (21.03 to 30.31) | 33.06 (27.34 to 38.78) | 38.92 (34.26 to 43.59) | 46.25 (36.41 to 56.10) | 43.94 (33.11 to 54.77) |
|  |  | 50 to 59 | 35.01 (30.08 to 39.95) | 52.15 (47.36 to 56.94) | 50.42 (45.63 to 55.20) | 50.98 (46.18 to 55.78) | 62.60 (58.69 to 66.51) | 60.92 (54.19 to 67.65) | 73.86 (66.44 to 81.29) |
|  |  | 60 to 69 | 45.04 (39.47 to 50.61) | 70.60 (65.88 to 75.33) | 69.22 (65.07 to 73.37) | 76.48 (73.05 to 79.91) | 80.22 (77.46 to 82.98) | 82.49 (75.81 to 89.17) | 69.97 (63.19 to 76.74) |
|  |  | 70 to 79 | 52.07 (44.22 to 59.92) | 67.37 (62.71 to 72.03) | 76.59 (72.45 to 80.72) | 79.46 (75.63 to 83.29) | 84.95 (82.36 to 87.55) | 86.00 (81.18 to 90.82) | 90.17 (86.38 to 93.96) |
|  |  | ≥80 | 33.07 (15.70 to 50.44) | 66.73 (57.00 to 76.47) | 72.38 (61.64 to 83.12) | 73.29 (64.47 to 82.12) | 83.60 (79.26 to 87.94) | 73.32 (60.56 to 86.09) | 87.06 (80.13 to 93.99) |
|  | Control | 19 to 29 | 1.60 (0.00 to 4.66) | 5.14 (0.00 to 10.84) | N/A | 1.72 (0.00 to 5.08) | 7.85 (0.81 to 14.89) | 3.09 (0.00 to 9.14) | 3.73 (0.00 to 11.24) |
|  |  | 30 to 39 | 4.89 (1.12 to 8.67) | 5.97 (2.79 to 9.16) | 5.81 (2.44 to 9.19) | 8.54 (4.48 to 12.59) | 9.73 (5.69 to 13.78) | 10.01 (1.32 to 18.69) | 28.38 (13.11 to 43.66) |
|  |  | 40 to 49 | 7.63 (4.81 to 10.44) | 14.62 (10.73 to 18.51) | 14.15 (10.56 to 17.74) | 21.85 (16.95 to 26.76) | 27.02 (22.97 to 31.08) | 34.29 (25.24 to 43.34) | 37.51 (26.92 to 48.11) |
|  |  | 50 to 59 | 17.01 (12.34 to 21.68) | 32.33 (27.72 to 36.94) | 32.42 (28.17 to 36.67) | 33.86 (29.27 to 38.45) | 45.06 (41.16 to 48.97) | 43.05 (35.89 to 50.20) | 62.79 (53.50 to 72.07) |
|  |  | 60 to 69 | 19.80 (15.36 to 24.24) | 45.01 (40.07 to 49.96) | 46.68 (42.39 to 50.96) | 59.00 (54.68 to 63.31) | 65.45 (62.11 to 68.79) | 62.34 (54.98 to 69.70) | 55.30 (47.77 to 62.83) |
|  |  | 70 to 79 | 17.65 (11.03 to 24.27) | 41.80 (36.75 to 46.86) | 53.52 (48.51 to 58.53) | 59.22 (54.18 to 64.26) | 64.03 (60.26 to 67.81) | 66.12 (58.97 to 73.26) | 66.56 (59.64 to 73.48) |
|  |  | ≥80 | 13.55 (0.20 to 26.89) | 42.68 (31.67 to 53.68) | 56.24 (44.48 to 68.00) | 56.66 (46.33 to 66.99) | 65.42 (59.43 to 71.41) | 46.11 (32.98 to 59.24) | 66.01 (55.63 to 76.39) |
|  | Control among treated | 19 to 29 | 49.02 (0.00 to 100.00) | 68.58 (30.36 to 100.00) | N/A | N/A | 89.72 (71.89 to 100.00) | N/A | 20.96 (0.00 to 66.89) |
|  |  | 30 to 39 | 66.61 (37.37 to 95.85) | 57.11 (35.72 to 78.51) | 68.67 (47.77 to 89.56) | 64.95 (44.91 to 84.99) | 66.13 (51.27 to 80.98) | 90.59 (72.10 to 100.00) | 78.45 (53.91 to 100.00) |
|  |  | 40 to 49 | 38.66 (26.37 to 50.95) | 55.77 (46.05 to 65.49) | 55.12 (45.02 to 65.22) | 66.10 (56.57 to 75.64) | 69.42 (62.96 to 75.89) | 74.14 (63.60 to 84.68) | 85.38 (75.55 to 95.21) |
|  |  | 50 to 59 | 48.58 (38.44 to 58.71) | 61.99 (55.52 to 68.46) | 64.30 (58.37 to 70.24) | 66.42 (60.00 to 72.84) | 71.99 (67.66 to 76.32) | 70.66 (61.96 to 79.35) | 85.00 (77.67 to 92.33) |
|  |  | 60 to 69 | 43.96 (35.75 to 52.16) | 63.75 (58.20 to 69.31) | 67.44 (62.50 to 72.37) | 77.14 (72.64 to 81.63) | 81.59 (78.51 to 84.66) | 75.58 (68.64 to 82.51) | 79.04 (72.14 to 85.94) |
|  |  | 70 to 79 | 33.90 (23.11 to 44.70) | 62.05 (55.65 to 68.44) | 69.88 (64.65 to 75.11) | 74.53 (69.50 to 79.56) | 75.38 (71.54 to 79.21) | 76.88 (69.61 to 84.15) | 73.82 (66.70 to 80.93) |
|  |  | ≥80 | 40.97 (6.42 to 75.52) | 63.95 (49.52 to 78.38) | 77.70 (67.53 to 87.87) | 77.30 (67.11 to 87.49) | 78.25 (72.13 to 84.37) | 62.89 (48.89 to 76.88) | 75.82 (66.01 to 85.64) |
|  | | | | | | | | | |
| Women | Prevalence | 19 to 29 | 1.00 (0.43 to 1.57) | 1.66 (0.86 to 2.45) | 1.06 (0.42 to 1.71) | 1.08 (0.50 to 1.66) | 2.01 (1.20 to 2.82) | 1.08 (0.00 to 2.17) | 1.59 (0.00 to 3.21) |
|  |  | 30 to 39 | 4.47 (3.37 to 5.57) | 4.43 (3.38 to 5.49) | 3.38 (2.41 to 4.35) | 2.20 (1.40 to 3.01) | 4.16 (3.24 to 5.08) | 4.40 (2.39 to 6.40) | 1.95 (0.53 to 3.37) |
|  |  | 40 to 49 | 13.21 (11.27 to 15.16) | 14.75 (12.84 to 16.66) | 14.50 (12.50 to 16.50) | 10.37 (8.71 to 12.04) | 11.66 (10.21 to 13.11) | 9.63 (6.93 to 12.33) | 13.43 (9.86 to 17.00) |
|  |  | 50 to 59 | 35.61 (32.23 to 38.99) | 33.47 (30.76 to 36.18) | 32.17 (29.63 to 34.70) | 28.07 (25.83 to 30.31) | 28.37 (26.40 to 30.34) | 26.38 (22.67 to 30.09) | 26.52 (22.65 to 30.39) |
|  |  | 60 to 69 | 54.93 (50.94 to 58.92) | 51.60 (48.58 to 54.62) | 56.82 (53.80 to 59.84) | 48.46 (45.59 to 51.32) | 47.02 (44.59 to 49.46) | 45.90 (41.17 to 50.62) | 45.52 (40.80 to 50.24) |
|  |  | 70 to 79 | 64.61 (60.40 to 68.83) | 64.00 (60.64 to 67.37) | 66.97 (63.92 to 70.01) | 62.15 (58.58 to 65.71) | 68.41 (65.67 to 71.16) | 67.81 (63.25 to 72.37) | 66.67 (61.50 to 71.85) |
|  |  | ≥80 | 62.53 (54.20 to 70.85) | 70.06 (64.33 to 75.79) | 69.84 (63.69 to 75.99) | 65.90 (60.70 to 71.10) | 78.08 (74.74 to 81.42) | 72.90 (66.57 to 79.24) | 75.94 (70.08 to 81.80) |
|  | Awareness | 19 to 29 | 8.18 (0.00 to 23.61) | N/A | 18.22 (0.00 to 41.02) | 6.27 (0.00 to 18.26) | 18.59 (4.35 to 32.84) | N/A | N/A |
|  |  | 30 to 39 | 12.43 (4.29 to 20.57) | 23.08 (13.31 to 32.86) | 31.54 (17.59 to 45.50) | 15.50 (3.24 to 27.76) | 23.13 (12.86 to 33.41) | 22.72 (4.36 to 41.08) | 36.34 (0.00 to 75.13) |
|  |  | 40 to 49 | 33.11 (25.97 to 40.24) | 50.58 (44.39 to 56.76) | 46.07 (38.87 to 53.27) | 50.09 (41.98 to 58.21) | 47.35 (41.05 to 53.65) | 51.41 (36.82 to 66.01) | 45.05 (30.30 to 59.81) |
|  |  | 50 to 59 | 54.89 (49.36 to 60.43) | 70.51 (65.97 to 75.05) | 66.66 (62.56 to 70.76) | 62.69 (58.05 to 67.33) | 67.98 (64.28 to 71.67) | 65.81 (57.35 to 74.27) | 73.03 (64.88 to 81.18) |
|  |  | 60 to 69 | 64.45 (59.77 to 69.13) | 81.87 (78.61 to 85.13) | 77.94 (74.45 to 81.44) | 80.23 (76.96 to 83.51) | 79.30 (76.50 to 82.10) | 81.02 (76.17 to 85.86) | 83.90 (79.14 to 88.66) |
|  |  | 70 to 79 | 58.94 (53.25 to 64.62) | 79.20 (76.10 to 82.30) | 83.09 (79.85 to 86.34) | 84.33 (81.23 to 87.43) | 86.48 (84.21 to 88.74) | 86.49 (81.91 to 91.06) | 88.37 (83.86 to 92.88) |
|  |  | ≥80 | 49.18 (38.31 to 60.04) | 73.06 (65.28 to 80.84) | 81.22 (75.28 to 87.16) | 74.57 (68.81 to 80.33) | 88.60 (85.33 to 91.87) | 86.07 (79.11 to 93.02) | 87.37 (81.84 to 92.90) |
|  | Treatment | 19 to 29 | 11.37 (0.00 to 27.87) | N/A | 14.53 (0.00 to 36.37) | N/A | 18.59 (4.47 to 32.72) | N/A | N/A |
|  |  | 30 to 39 | 8.76 (1.79 to 15.74) | 15.82 (7.45 to 24.19) | 22.70 (10.65 to 34.76) | 15.50 (3.24 to 27.77) | 19.88 (10.39 to 29.36) | 21.31 (2.99 to 39.62) | 36.34 (0.00 to 74.83) |
|  |  | 40 to 49 | 30.50 (23.93 to 37.07) | 46.25 (39.99 to 52.51) | 40.59 (33.51 to 47.67) | 47.67 (39.38 to 55.96) | 44.15 (37.85 to 50.45) | 49.92 (35.31 to 64.53) | 44.41 (29.81 to 59.02) |
|  |  | 50 to 59 | 50.08 (44.33 to 55.83) | 63.85 (59.17 to 68.52) | 61.94 (57.73 to 66.15) | 60.19 (55.53 to 64.85) | 65.10 (61.31 to 68.88) | 59.20 (50.37 to 68.04) | 70.75 (61.16 to 80.35) |
|  |  | 60 to 69 | 59.92 (54.96 to 64.89) | 77.68 (73.90 to 81.47) | 75.57 (71.90 to 79.24) | 78.78 (75.44 to 82.12) | 77.30 (74.43 to 80.17) | 78.69 (73.07 to 84.32) | 82.25 (77.32 to 87.18) |
|  |  | 70 to 79 | 57.14 (51.54 to 62.73) | 76.17 (72.82 to 79.52) | 81.62 (78.28 to 84.96) | 83.42 (80.27 to 86.57) | 84.87 (82.54 to 87.20) | 85.69 (81.02 to 90.36) | 86.51 (81.70 to 91.32) |
|  |  | ≥80 | 42.20 (31.47 to 52.94) | 65.65 (57.50 to 73.79) | 80.27 (74.34 to 86.19) | 73.23 (67.27 to 79.19) | 85.87 (82.26 to 89.47) | 84.75 (77.41 to 92.10) | 86.20 (80.43 to 91.98) |
|  | Control | 19 to 29 | 11.37 (0.00 to 27.88) | N/A | N/A | N/A | 16.29 (2.43 to 30.15) | N/A | N/A |
|  |  | 30 to 39 | 6.44 (0.08 to 12.80) | 10.84 (3.15 to 18.54) | 13.95 (3.75 to 24.15) | 9.77 (0.09 to 19.45) | 17.23 (8.14 to 26.32) | 11.17 (0.00 to 24.00) | 36.34 (0.00 to 75.13) |
|  |  | 40 to 49 | 15.32 (10.08 to 20.55) | 30.55 (24.38 to 36.72) | 25.27 (18.89 to 31.64) | 42.55 (34.55 to 50.56) | 36.82 (30.52 to 43.11) | 38.14 (24.01 to 52.27) | 39.37 (25.27 to 53.48) |
|  |  | 50 to 59 | 27.48 (22.36 to 32.61) | 40.77 (35.79 to 45.76) | 39.04 (34.69 to 43.38) | 43.83 (38.93 to 48.74) | 49.07 (44.92 to 53.22) | 43.32 (33.98 to 52.65) | 59.99 (50.25 to 69.73) |
|  |  | 60 to 69 | 28.22 (23.65 to 32.79) | 52.42 (47.84 to 57.00) | 47.01 (43.01 to 51.02) | 56.79 (52.68 to 60.90) | 54.16 (50.71 to 57.60) | 62.37 (54.71 to 70.04) | 66.41 (59.95 to 72.86) |
|  |  | 70 to 79 | 24.00 (19.24 to 28.76) | 45.83 (41.40 to 50.26) | 52.50 (48.44 to 56.56) | 56.73 (52.49 to 60.98) | 56.91 (53.64 to 60.18) | 50.80 (44.50 to 57.09) | 66.24 (59.78 to 72.69) |
|  |  | ≥80 | 15.11 (7.52 to 22.71) | 44.13 (36.14 to 52.11) | 46.09 (38.39 to 53.79) | 49.25 (42.73 to 55.78) | 53.34 (48.18 to 58.51) | 59.54 (49.86 to 69.21) | 54.17 (45.86 to 62.47) |
|  | Control among treated | 19 to 29 | 99.97 (99.91 to 100.00) | N/A | N/A | N/A | 87.62 (63.81 to 100.00) | N/A | N/A |
|  |  | 30 to 39 | 73.47 (39.59 to 100.00) | 68.52 (43.47 to 93.57) | 61.45 (33.94 to 88.96) | 63.03 (22.21 to 100.00) | 86.67 (69.86 to 100.00) | 52.41 (3.15 to 100.00) | N/A |
|  |  | 40 to 49 | 50.22 (37.03 to 63.40) | 66.05 (56.64 to 75.46) | 62.25 (50.81 to 73.69) | 89.27 (82.79 to 95.76) | 83.38 (76.52 to 90.25) | 76.40 (59.76 to 93.04) | 88.65 (74.76 to 100.00) |
|  |  | 50 to 59 | 54.88 (47.02 to 62.74) | 63.86 (57.77 to 69.95) | 63.02 (57.34 to 68.70) | 72.82 (67.39 to 78.26) | 75.38 (70.86 to 79.90) | 73.17 (62.47 to 83.86) | 84.79 (76.35 to 93.22) |
|  |  | 60 to 69 | 47.10 (40.68 to 53.51) | 67.48 (62.87 to 72.08) | 62.21 (57.69 to 66.74) | 72.09 (67.90 to 76.28) | 70.07 (66.61 to 73.53) | 79.26 (73.00 to 85.52) | 80.74 (74.86 to 86.61) |
|  |  | 70 to 79 | 42.01 (34.91 to 49.10) | 60.16 (55.07 to 65.26) | 64.32 (60.18 to 68.47) | 68.01 (63.54 to 72.47) | 67.06 (63.68 to 70.44) | 59.28 (52.51 to 66.05) | 76.57 (70.26 to 82.87) |
|  |  | ≥80 | 35.81 (19.59 to 52.02) | 67.22 (58.68 to 75.75) | 57.42 (49.56 to 65.28) | 67.26 (60.41 to 74.11) | 62.12 (56.77 to 67.48) | 70.25 (61.74 to 78.75) | 62.83 (53.84 to 71.83) |

CI, confidence interval.

**Supplementary Table 5.** Trends in prevalence, awareness, treatment, control, and control among treated rates by BMI group, weighted % (95% CI)

| Sex | Prevalence | Body mass index group, kg/m^2^, n (%) | 1998 to 2005 | 2007 to 2009 | 2010 to 2012 | 2013 to 2015 | 2016 to 2019 | 2020 | 2021 |
| --- | --- | --- | --- | --- | --- | --- | --- | --- | --- |
| Men | Prevalence | Normal or underweight (>23.0) | 21.73 (19.69 to 23.77) | 18.68 (16.93 to 20.43) | 20.41 (18.65 to 22.16) | 17.37 (15.84 to 18.90) | 20.99 (19.51 to 22.47) | 19.85 (16.23 to 23.46) | 21.48 (18.00 to 24.96) |
|  |  | Overweight (23.0 to 25.0) | 29.14 (26.21 to 32.07) | 27.44 (25.23 to 29.65) | 29.90 (27.53 to 32.26) | 23.65 (21.52 to 25.78) | 28.90 (26.94 to 30.85) | 29.15 (25.74 to 32.57) | 26.12 (22.12 to 30.13) |
|  |  | Obese (≥25.0) | 40.41 (37.65 to 43.18) | 37.66 (35.36 to 39.97) | 41.19 (38.89 to 43.49) | 38.03 (35.81 to 40.25) | 39.05 (37.41 to 40.70) | 42.27 (38.75 to 45.79) | 37.47 (33.92 to 41.02) |
|  | Awareness | Normal or underweight (>23.0) | 33.30 (28.35 to 38.25) | 48.31 (43.50 to 53.12) | 46.22 (42.31 to 50.13) | 56.04 (51.36 to 60.71) | 63.85 (60.13 to 67.58) | 65.90 (56.90 to 74.90) | 68.04 (60.35 to 75.73) |
|  |  | Overweight (23.0 to 25.0) | 33.62 (28.48 to 38.77) | 50.90 (45.92 to 55.87) | 48.98 (44.25 to 53.70) | 58.31 (53.39 to 63.24) | 63.52 (59.82 to 67.21) | 59.94 (52.47 to 67.41) | 74.50 (67.32 to 81.69) |
|  |  | Obese (≥25.0) | 36.17 (32.17 to 40.17) | 50.44 (47.01 to 53.88) | 47.34 (43.85 to 50.84) | 51.99 (48.45 to 55.54) | 61.61 (58.89 to 64.33) | 65.83 (60.66 to 71.00) | 68.81 (62.94 to 74.67) |
|  | Treatment | Normal or underweight (>23.0) | 29.24 (24.56 to 33.91) | 42.17 (37.43 to 46.92) | 42.51 (38.67 to 46.34) | 51.65 (46.87 to 56.44) | 58.50 (54.68 to 62.33) | 63.91 (54.31 to 73.52) | 64.20 (56.50 to 71.90) |
|  |  | Overweight (23.0 to 25.0) | 27.04 (22.22 to 31.87) | 42.44 (37.60 to 47.29) | 44.80 (40.34 to 49.25) | 55.75 (50.92 to 60.58) | 60.94 (57.14 to 64.75) | 58.51 (51.29 to 65.74) | 71.61 (64.33 to 78.88) |
|  |  | Obese (≥25.0) | 30.13 (26.43 to 33.83) | 42.91 (39.23 to 46.58) | 42.52 (39.03 to 46.00) | 48.16 (44.67 to 51.65) | 57.79 (55.07 to 60.51) | 59.23 (53.80 to 64.66) | 64.30 (58.36 to 70.24) |
|  | Control | Normal or underweight (>23.0) | 13.51 (9.75 to 17.26) | 25.76 (21.25 to 30.28) | 29.06 (25.49 to 32.63) | 39.97 (35.25 to 44.70) | 43.85 (39.98 to 47.72) | 47.61 (38.97 to 56.24) | 52.03 (44.02 to 60.05) |
|  |  | Overweight (23.0 to 25.0) | 10.82 (7.32 to 14.33) | 28.28 (23.85 to 32.71) | 27.97 (24.08 to 31.87) | 39.75 (35.28 to 44.22) | 48.21 (44.46 to 51.96) | 43.66 (36.05 to 51.26) | 58.64 (51.36 to 65.92) |
|  |  | Obese (≥25.0) | 13.06 (10.27 to 15.84) | 25.33 (22.25 to 28.40) | 27.96 (24.92 to 31.00) | 33.37 (30.37 to 36.37) | 42.78 (40.09 to 45.48) | 43.44 (38.14 to 48.74) | 50.66 (44.44 to 56.88) |
|  | Control among treated | Normal or underweight (>23.0) | 46.20 (36.55 to 55.85) | 61.09 (54.18 to 68.01) | 68.36 (62.84 to 73.87) | 77.39 (71.83 to 82.94) | 74.95 (70.58 to 79.33) | 74.48 (66.20 to 82.77) | 81.05 (73.48 to 88.62) |
|  |  | Overweight (23.0 to 25.0) | 40.03 (29.79 to 50.26) | 66.62 (60.21 to 73.04) | 62.45 (56.50 to 68.39) | 71.30 (65.59 to 77.02) | 79.10 (75.48 to 82.72) | 74.61 (66.54 to 82.68) | 81.89 (75.61 to 88.17) |
|  |  | Obese (≥25.0) | 43.33 (36.12 to 50.54) | 59.03 (53.53 to 64.53) | 65.75 (61.07 to 70.43) | 69.29 (65.28 to 73.31) | 74.03 (71.11 to 76.96) | 73.34 (67.89 to 78.79) | 78.78 (73.34 to 84.22) |
|  |  |  |  |  |  |  |  |  |  |
| Women | Prevalence | Normal or underweight (>23.0) | 12.11 (10.77 to 13.46) | 11.72 (10.66 to 12.77) | 14.12 (13.00 to 15.25) | 12.65 (11.60 to 13.71) | 14.30 (13.32 to 15.28) | 14.09 (11.76 to 16.42) | 15.35 (13.16 to 17.55) |
|  |  | Overweight (23.0 to 25.0) | 22.93 (20.29 to 25.57) | 25.10 (23.03 to 27.17) | 28.03 (25.85 to 30.21) | 24.38 (22.23 to 26.53) | 29.65 (27.61 to 31.68) | 28.78 (24.40 to 33.15) | 28.24 (24.11 to 32.38) |
|  |  | Obese (≥25.0) | 37.63 (35.29 to 39.96) | 39.98 (37.81 to 42.15) | 38.97 (36.67 to 41.28) | 38.03 (35.94 to 40.11) | 41.52 (39.63 to 43.40) | 37.85 (34.27 to 41.44) | 41.35 (37.34 to 45.37) |
|  | Awareness | Normal or underweight (>23.0) | 46.20 (40.76 to 51.64) | 64.08 (59.96 to 68.19) | 67.18 (63.51 to 70.86) | 63.53 (59.33 to 67.72) | 67.45 (64.44 to 70.46) | 71.77 (65.35 to 78.19) | 71.68 (65.21 to 78.16) |
|  |  | Overweight (23.0 to 25.0) | 49.54 (44.29 to 54.79) | 68.88 (64.42 to 73.34) | 69.73 (65.44 to 74.01) | 73.74 (69.66 to 77.83) | 77.15 (73.97 to 80.33) | 77.03 (70.96 to 83.09) | 74.87 (67.85 to 81.89) |
|  |  | Obese (≥25.0) | 57.70 (53.83 to 61.57) | 73.19 (70.09 to 76.28) | 73.97 (71.03 to 76.91) | 75.86 (73.01 to 78.71) | 77.69 (75.36 to 80.03) | 75.80 (70.99 to 80.60) | 83.83 (79.83 to 87.83) |
|  | Treatment | Normal or underweight (>23.0) | 44.10 (38.97 to 49.23) | 58.73 (54.42 to 63.04) | 63.25 (59.55 to 66.96) | 61.80 (57.60 to 66.01) | 64.26 (61.21 to 67.31) | 67.61 (60.94 to 74.28) | 69.60 (62.88 to 76.31) |
|  |  | Overweight (23.0 to 25.0) | 44.15 (39.09 to 49.22) | 64.24 (59.48 to 68.99) | 65.46 (61.08 to 69.85) | 71.98 (67.95 to 76.01) | 75.00 (71.75 to 78.26) | 75.78 (69.65 to 81.92) | 73.47 (66.34 to 80.60) |
|  |  | Obese (≥25.0) | 53.73 (49.74 to 57.72) | 68.45 (65.05 to 71.84) | 71.83 (68.81 to 74.86) | 74.28 (71.38 to 77.17) | 75.83 (73.40 to 78.25) | 73.30 (68.47 to 78.14) | 82.53 (78.16 to 86.91) |
|  | Control | Normal or underweight (>23.0) | 21.17 (16.81 to 25.53) | 37.86 (33.29 to 42.43) | 39.82 (35.76 to 43.88) | 42.93 (38.76 to 47.09) | 43.79 (40.54 to 47.04) | 48.72 (40.97 to 56.48) | 52.67 (45.69 to 59.65) |
|  |  | Overweight (23.0 to 25.0) | 23.04 (18.32 to 27.76) | 43.58 (38.41 to 48.75) | 41.02 (36.74 to 45.29) | 50.88 (46.42 to 55.34) | 50.94 (47.39 to 54.50) | 49.97 (42.64 to 57.30) | 61.29 (53.88 to 68.70) |
|  |  | Obese (≥25.0) | 24.88 (21.30 to 28.45) | 43.18 (39.72 to 46.63) | 44.46 (41.15 to 47.77) | 54.19 (50.81 to 57.58) | 54.52 (51.90 to 57.13) | 52.38 (47.51 to 57.24) | 63.80 (58.61 to 68.98) |
|  | Control among treated | Normal or underweight (>23.0) | 48.01 (40.12 to 55.89) | 64.47 (58.98 to 69.95) | 62.95 (57.93 to 67.97) | 69.46 (64.87 to 74.04) | 68.14 (64.41 to 71.87) | 72.06 (64.10 to 80.02) | 75.68 (68.94 to 82.41) |
|  |  | Overweight (23.0 to 25.0) | 52.19 (43.39 to 60.98) | 67.84 (62.43 to 73.26) | 62.65 (57.39 to 67.92) | 70.68 (66.21 to 75.15) | 67.92 (64.07 to 71.77) | 65.93 (57.66 to 74.21) | 83.42 (77.44 to 89.40) |
|  |  | Obese (≥25.0) | 46.30 (40.96 to 51.63) | 63.08 (59.25 to 66.91) | 61.89 (58.02 to 65.77) | 72.96 (69.45 to 76.47) | 71.90 (69.34 to 74.45) | 71.45 (66.30 to 76.61) | 77.30 (72.40 to 82.19) |

BMI, Body mass index; CI, confidence interval.

**Supplementary Table 6.** Trends in hypertension prevalence, awareness, treatment, control and control among treated rates by central obesity status, weighted % (95% CI)

| Sex | Prevalence | Central obesity | 1998 to 2005 | 2007 to 2009 | 2010 to 2012 | 2013 to 2015 | 2016 to 2019 | 2020 | 2021 |
| --- | --- | --- | --- | --- | --- | --- | --- | --- | --- |
| Men | Hypertension | No | 24.64 (22.91 to 26.38) | 22.61 (21.18 to 24.04) | 25.44 (23.93 to 26.95) | 21.33 (20.04 to 22.61) | 23.89 (22.72 to 25.06) | 22.10 (19.41 to 24.78) | 21.43 (18.90 to 23.95) |
|  |  | Yes | 46.31 (43.06 to 49.55) | 43.02 (40.33 to 45.72) | 44.37 (41.66 to 47.08) | 42.00 (39.37 to 44.63) | 43.52 (41.68 to 45.36) | 46.53 (43.23 to 49.83) | 42.33 (38.70 to 45.96) |
|  | Awareness | No | 31.63 (28.29 to 34.96) | 46.96 (43.71 to 50.20) | 43.70 (40.69 to 46.71) | 52.93 (49.73 to 56.14) | 60.34 (57.75 to 62.93) | 60.11 (53.59 to 66.63) | 67.37 (61.98 to 72.77) |
|  |  | Yes | 39.66 (35.26 to 44.07) | 54.74 (51.04 to 58.44) | 53.60 (49.54 to 57.65) | 56.06 (52.04 to 60.08) | 65.08 (62.40 to 67.77) | 67.29 (62.41 to 72.17) | 72.00 (66.57 to 77.43) |
|  | Treatment | No | 26.38 (23.23 to 29.54) | 40.10 (36.91 to 43.29) | 39.93 (37.10 to 42.77) | 49.27 (46.17 to 52.38) | 56.13 (53.40 to 58.87) | 58.06 (51.70 to 64.41) | 64.08 (58.74 to 69.43) |
|  |  | Yes | 33.47 (29.46 to 37.49) | 33.47 (29.46 to 37.49) | 33.47 (29.46 to 37.49) | 33.47 (29.46 to 37.49) | 33.47 (29.46 to 37.49) | 33.47 (29.46 to 37.49) | 33.47 (29.46 to 37.49) |
|  | Control | No | 11.79 (9.49 to 14.09) | 24.59 (21.62 to 27.55) | 26.56 (24.09 to 29.04) | 36.02 (33.10 to 38.95) | 42.96 (40.21 to 45.71) | 41.85 (36.23 to 47.47) | 52.47 (46.78 to 58.15) |
|  |  | Yes | 13.98 (10.71 to 17.25) | 28.82 (25.43 to 32.20) | 31.13 (27.60 to 34.65) | 36.73 (33.22 to 40.23) | 46.01 (43.24 to 48.78) | 45.79 (40.87 to 50.72) | 53.05 (47.26 to 58.84) |
|  | Control among treated | No | 44.68 (37.79 to 51.58) | 44.68 (37.79 to 51.58) | 44.68 (37.79 to 51.58) | 44.68 (37.79 to 51.58) | 44.68 (37.79 to 51.58) | 44.68 (37.79 to 51.58) | 44.68 (37.79 to 51.58) |
|  |  | Yes | 41.77 (34.08 to 49.45) | 41.77 (34.08 to 49.45) | 41.77 (34.08 to 49.45) | 41.77 (34.08 to 49.45) | 41.77 (34.08 to 49.45) | 41.77 (34.08 to 49.45) | 41.77 (34.08 to 49.45) |
|  | | | | | | | | | |
| Women | Hypertension | No | 15.25 (13.94 to 16.56) | 15.10 (14.04 to 16.16) | 17.47 (16.38 to 18.57) | 15.89 (14.84 to 16.94) | 17.19 (16.21 to 18.17) | 16.25 (14.06 to 18.44) | 17.15 (15.25 to 19.06) |
|  |  | Yes | 42.74 (40.05 to 45.44) | 44.15 (41.83 to 46.47) | 44.27 (41.78 to 46.77) | 42.60 (40.27 to 44.93) | 47.55 (45.66 to 49.44) | 44.54 (40.81 to 48.27) | 46.96 (43.09 to 50.84) |
|  | Awareness | No | 46.82 (42.67 to 50.97) | 63.83 (60.44 to 67.23) | 66.87 (63.93 to 69.81) | 66.14 (63.10 to 69.17) | 69.39 (66.94 to 71.84) | 71.76 (66.94 to 76.58) | 72.41 (67.40 to 77.42) |
|  |  | Yes | 59.24 (55.35 to 63.12) | 75.75 (72.69 to 78.80) | 75.83 (72.65 to 79.01) | 78.86 (76.11 to 81.60) | 80.00 (77.83 to 82.17) | 79.33 (75.35 to 83.31) | 83.52 (79.77 to 87.27) |
|  | Treatment | No | 43.40 (39.60 to 47.21) | 57.55 (54.03 to 61.07) | 62.63 (59.68 to 65.58) | 64.33 (61.34 to 67.32) | 66.50 (64.05 to 68.94) | 68.47 (63.18 to 73.75) | 69.88 (64.30 to 75.45) |
|  |  | Yes | 55.00 (51.14 to 58.86) | 72.26 (69.05 to 75.48) | 73.99 (70.80 to 77.18) | 77.36 (74.56 to 80.17) | 78.27 (76.05 to 80.50) | 77.34 (73.30 to 81.39) | 82.65 (78.95 to 86.35) |
|  | Control | No | 21.83 (18.81 to 24.84) | 37.23 (33.74 to 40.71) | 39.10 (35.95 to 42.25) | 44.41 (41.18 to 47.63) | 46.23 (43.53 to 48.94) | 48.57 (42.27 to 54.87) | 55.09 (48.85 to 61.34) |
|  |  | Yes | 25.17 (21.39 to 28.96) | 46.55 (43.12 to 49.98) | 46.18 (42.60 to 49.75) | 57.31 (54.10 to 60.52) | 54.95 (52.40 to 57.49) | 53.93 (49.21 to 58.64) | 63.79 (58.68 to 68.89) |
|  | Control among treated | No | 50.29 (44.52 to 56.06) | 64.68 (60.71 to 68.66) | 62.43 (58.61 to 66.26) | 69.03 (65.40 to 72.66) | 69.53 (66.60 to 72.45) | 70.94 (64.52 to 77.37) | 78.84 (73.43 to 84.26) |
|  |  | Yes | 45.77 (40.09 to 51.45) | 64.42 (60.68 to 68.15) | 62.41 (58.44 to 66.38) | 74.08 (70.81 to 77.35) | 70.20 (67.69 to 72.71) | 69.72 (64.90 to 74.54) | 77.18 (72.50 to 81.85) |

CI, confidence interval.

Central obesity was defined as having waist circumference ≥90 cm for men and ≥85 cm for women.

**Supplementary Table 7.** Trends in hypertension prevalence, awareness, treatment, control and control among treated rates by income level, weighted % (95% CI)

| Sex | Prevalence | Income | 1998 to 2005 | 2007 to 2009 | 2010 to 2012 | 2013 to 2015 | 2016 to 2019 | 2020 | 2021 |
| --- | --- | --- | --- | --- | --- | --- | --- | --- | --- |
| Men | Prevalence | Lowest quartile | 41.49 (38.13 to 44.84) | 38.74 (35.59 to 41.90) | 40.45 (36.85 to 44.04) | 38.55 (35.05 to 42.05) | 44.40 (41.30 to 47.49) | 45.76 (40.11 to 51.42) | 42.19 (35.24 to 49.13) |
|  |  | Second quartile | 28.11 (25.02 to 31.21) | 29.48 (27.02 to 31.95) | 30.35 (27.66 to 33.03) | 27.21 (24.92 to 29.49) | 32.34 (30.19 to 34.48) | 37.97 (33.39 to 42.54) | 33.02 (28.37 to 37.67) |
|  |  | Third quartile | 26.25 (23.63 to 28.88) | 24.89 (22.68 to 27.10) | 27.71 (25.38 to 30.04) | 25.19 (23.10 to 27.27) | 25.70 (24.03 to 27.38) | 30.79 (26.75 to 34.84) | 30.31 (26.27 to 34.36) |
|  |  | Highest quartile | 26.19 (23.66 to 28.72) | 24.53 (22.43 to 26.62) | 27.47 (25.10 to 29.84) | 23.18 (21.03 to 25.34) | 27.91 (26.17 to 29.64) | 27.14 (24.03 to 30.25) | 24.12 (20.79 to 27.45) |
|  | Awareness | Lowest quartile | 40.96 (36.15 to 45.77) | 58.29 (53.22 to 63.35) | 61.11 (55.84 to 66.38) | 71.21 (66.42 to 76.00) | 77.08 (73.34 to 80.82) | 76.30 (68.42 to 84.19) | 80.06 (73.00 to 87.11) |
|  |  | Second quartile | 34.75 (29.82 to 39.68) | 49.76 (44.89 to 54.63) | 46.61 (42.22 to 51.01) | 55.15 (50.14 to 60.16) | 63.80 (60.12 to 67.47) | 65.38 (57.40 to 73.35) | 75.76 (68.96 to 82.55) |
|  |  | Third quartile | 31.63 (26.37 to 36.90) | 42.82 (38.04 to 47.59) | 38.73 (34.52 to 42.93) | 44.12 (39.65 to 48.60) | 54.27 (50.34 to 58.20) | 59.15 (51.55 to 66.75) | 62.48 (55.60 to 69.36) |
|  |  | Highest quartile | 32.51 (27.52 to 37.49) | 49.75 (44.89 to 54.61) | 47.48 (42.87 to 52.09) | 51.53 (46.73 to 56.32) | 59.54 (56.13 to 62.96) | 63.03 (56.54 to 69.51) | 68.16 (59.80 to 76.52) |
|  | Treatment | Lowest quartile | 33.59 (29.09 to 38.09) | 53.94 (48.98 to 58.91) | 58.24 (53.06 to 63.43) | 69.55 (64.76 to 74.34) | 72.87 (68.99 to 76.75) | 74.06 (66.41 to 81.71) | 76.20 (69.08 to 83.32) |
|  |  | Second quartile | 30.65 (25.74 to 35.56) | 41.82 (37.41 to 46.23) | 41.53 (37.10 to 45.95) | 51.88 (46.74 to 57.02) | 59.62 (55.89 to 63.34) | 62.15 (53.85 to 70.44) | 70.30 (63.20 to 77.39) |
|  |  | Third quartile | 26.96 (21.77 to 32.16) | 33.41 (28.96 to 37.87) | 34.13 (30.13 to 38.13) | 39.54 (35.32 to 43.76) | 50.96 (46.99 to 54.92) | 50.63 (42.63 to 58.63) | 58.18 (50.85 to 65.50) |
|  |  | Highest quartile | 26.06 (21.58 to 30.55) | 42.37 (37.38 to 47.37) | 42.99 (38.60 to 47.37) | 47.55 (42.82 to 52.28) | 55.62 (52.12 to 59.11) | 59.33 (52.96 to 65.71) | 65.66 (57.51 to 73.82) |
|  | Control | Lowest quartile | 12.96 (9.74 to 16.18) | 31.81 (27.36 to 36.26) | 39.41 (34.55 to 44.27) | 51.36 (46.55 to 56.17) | 56.27 (52.20 to 60.34) | 52.46 (44.46 to 60.46) | 57.01 (49.27 to 64.76) |
|  |  | Second quartile | 13.83 (9.85 to 17.81) | 25.24 (21.22 to 29.25) | 27.97 (23.99 to 31.94) | 37.94 (33.29 to 42.59) | 43.43 (39.77 to 47.08) | 47.96 (39.78 to 56.15) | 56.43 (48.51 to 64.36) |
|  |  | Third quartile | 13.05 (8.75 to 17.35) | 21.29 (17.70 to 24.88) | 21.79 (18.31 to 25.27) | 27.45 (23.51 to 31.38) | 39.58 (35.72 to 43.45) | 36.34 (29.50 to 43.19) | 45.78 (38.81 to 52.74) |
|  |  | Highest quartile | 11.20 (7.79 to 14.62) | 27.30 (22.67 to 31.94) | 27.58 (23.78 to 31.38) | 33.55 (29.24 to 37.87) | 41.76 (38.20 to 45.32) | 44.61 (37.39 to 51.84) | 54.27 (45.28 to 63.26) |
|  | Control among treated | Lowest quartile | 38.58 (30.35 to 46.81) | 58.97 (52.78 to 65.16) | 67.66 (62.38 to 72.93) | 73.85 (68.88 to 78.82) | 77.22 (73.44 to 81.00) | 70.84 (63.42 to 78.25) | 74.82 (67.46 to 82.18) |
|  |  | Second quartile | 45.13 (34.89 to 55.38) | 60.34 (53.35 to 67.33) | 67.35 (61.62 to 73.08) | 73.13 (67.63 to 78.63) | 72.84 (68.82 to 76.86) | 77.17 (69.26 to 85.09) | 80.28 (72.67 to 87.88) |
|  |  | Third quartile | 48.41 (37.00 to 59.83) | 63.72 (56.52 to 70.93) | 63.84 (57.03 to 70.66) | 69.41 (62.97 to 75.85) | 77.67 (73.50 to 81.85) | 71.78 (63.56 to 80.00) | 78.68 (71.07 to 86.30) |
|  |  | Highest quartile | 42.99 (32.32 to 53.65) | 64.43 (56.94 to 71.93) | 64.15 (57.72 to 70.59) | 70.57 (64.44 to 76.69) | 75.09 (71.07 to 79.11) | 75.19 (66.38 to 84.01) | 82.65 (75.31 to 90.00) |
|  | | | | | | | | | |
| Women | Prevalence | Lowest quartile | 37.74 (34.77 to 40.71) | 42.84 (40.27 to 45.40) | 46.58 (43.73 to 49.43) | 43.34 (40.51 to 46.17) | 49.10 (46.71 to 51.50) | 44.90 (39.34 to 50.45) | 53.93 (48.62 to 59.23) |
|  |  | Second quartile | 21.13 (18.78 to 23.47) | 22.52 (20.63 to 24.42) | 22.82 (20.87 to 24.76) | 23.26 (21.39 to 25.13) | 26.29 (24.52 to 28.05) | 28.10 (24.26 to 31.95) | 30.03 (26.00 to 34.06) |
|  |  | Third quartile | 17.06 (15.08 to 19.03) | 16.07 (14.47 to 17.66) | 17.82 (16.11 to 19.52) | 17.32 (15.61 to 19.02) | 19.37 (17.83 to 20.91) | 21.03 (17.84 to 24.23) | 19.42 (16.49 to 22.36) |
|  |  | Highest quartile | 13.53 (11.69 to 15.37) | 14.75 (13.02 to 16.47) | 15.50 (13.94 to 17.07) | 12.04 (10.66 to 13.41) | 14.42 (13.20 to 15.64) | 15.67 (12.64 to 18.69) | 14.54 (12.08 to 16.99) |
|  | Awareness | Lowest quartile | 56.01 (51.87 to 60.15) | 74.33 (70.97 to 77.70) | 77.21 (74.02 to 80.41) | 78.97 (75.81 to 82.12) | 84.17 (81.98 to 86.35) | 86.40 (82.58 to 90.22) | 87.69 (83.43 to 91.95) |
|  |  | Second quartile | 55.29 (49.48 to 61.11) | 70.06 (65.65 to 74.48) | 71.45 (67.54 to 75.37) | 69.09 (64.77 to 73.41) | 74.15 (71.00 to 77.30) | 80.32 (74.82 to 85.82) | 75.74 (69.80 to 81.68) |
|  |  | Third quartile | 50.47 (44.30 to 56.64) | 62.71 (57.40 to 68.02) | 68.18 (63.41 to 72.94) | 70.07 (65.74 to 74.41) | 65.91 (61.93 to 69.89) | 67.12 (60.51 to 73.73) | 68.55 (60.17 to 76.94) |
|  |  | Highest quartile | 45.72 (38.47 to 52.97) | 66.24 (60.69 to 71.79) | 61.89 (56.44 to 67.34) | 61.91 (55.93 to 67.89) | 65.66 (61.39 to 69.93) | 63.81 (56.52 to 71.10) | 76.82 (67.86 to 85.78) |
|  | Treatment | Lowest quartile | 52.51 (48.37 to 56.65) | 68.79 (65.12 to 72.46) | 75.71 (72.56 to 78.87) | 77.07 (73.90 to 80.23) | 81.48 (79.14 to 83.81) | 84.43 (80.25 to 88.62) | 86.17 (81.83 to 90.50) |
|  |  | Second quartile | 52.54 (46.63 to 58.45) | 65.05 (60.35 to 69.75) | 67.69 (63.62 to 71.75) | 67.42 (63.07 to 71.76) | 71.71 (68.51 to 74.92) | 77.60 (71.94 to 83.26) | 74.30 (68.04 to 80.56) |
|  |  | Third quartile | 45.48 (39.41 to 51.54) | 59.37 (53.95 to 64.78) | 63.01 (58.20 to 67.82) | 68.32 (63.96 to 72.67) | 64.79 (60.76 to 68.82) | 63.81 (57.07 to 70.55) | 66.38 (57.94 to 74.81) |
|  |  | Highest quartile | 40.91 (34.38 to 47.43) | 61.21 (55.39 to 67.03) | 58.27 (52.67 to 63.87) | 60.88 (54.92 to 66.84) | 62.89 (58.66 to 67.13) | 61.44 (54.02 to 68.87) | 75.58 (66.78 to 84.38) |
|  | Control | Lowest quartile | 24.59 (20.82 to 28.35) | 42.78 (39.21 to 46.36) | 46.29 (42.35 to 50.24) | 53.40 (49.63 to 57.16) | 55.17 (52.11 to 58.23) | 57.48 (51.47 to 63.49) | 63.89 (58.08 to 69.69) |
|  |  | Second quartile | 23.86 (18.51 to 29.21) | 42.56 (38.01 to 47.12) | 42.77 (38.58 to 46.96) | 46.81 (42.50 to 51.12) | 50.97 (47.56 to 54.38) | 50.25 (41.87 to 58.62) | 57.56 (49.40 to 65.72) |
|  |  | Third quartile | 24.15 (18.88 to 29.41) | 36.13 (31.26 to 41.01) | 42.63 (37.61 to 47.64) | 52.39 (47.76 to 57.02) | 44.30 (40.26 to 48.35) | 47.73 (39.74 to 55.71) | 55.71 (47.09 to 64.32) |
|  |  | Highest quartile | 20.52 (14.84 to 26.20) | 41.47 (35.19 to 47.75) | 33.28 (28.16 to 38.39) | 44.57 (38.77 to 50.36) | 47.90 (43.39 to 52.42) | 47.14 (39.04 to 55.25) | 60.18 (51.20 to 69.15) |
|  | Control among treated | Lowest quartile | 46.82 (40.81 to 52.84) | 62.20 (58.34 to 66.05) | 61.14 (57.02 to 65.27) | 69.29 (65.49 to 73.08) | 67.71 (64.59 to 70.82) | 68.08 (62.22 to 73.94) | 74.14 (68.74 to 79.55) |
|  |  | Second quartile | 45.42 (37.59 to 53.24) | 65.43 (60.35 to 70.52) | 63.19 (58.18 to 68.20) | 69.43 (64.89 to 73.97) | 71.08 (67.47 to 74.68) | 64.75 (56.04 to 73.47) | 77.46 (70.01 to 84.92) |
|  |  | Third quartile | 53.10 (44.26 to 61.94) | 60.87 (54.33 to 67.40) | 67.65 (61.75 to 73.55) | 76.69 (71.99 to 81.39) | 68.38 (63.63 to 73.13) | 74.80 (65.92 to 83.68) | 83.93 (77.56 to 90.30) |
|  |  | Highest quartile | 50.17 (39.70 to 60.63) | 67.75 (61.07 to 74.44) | 57.11 (49.67 to 64.55) | 73.21 (67.17 to 79.24) | 76.17 (71.58 to 80.75) | 76.73 (66.86 to 86.60) | 79.62 (71.82 to 87.42) |

CI, confidence interval.

**Supplementary Table 8.** Trends in hypertension prevalence, awareness, treatment, control, and control among treated rates by drink frequency, weighted % (95% CI)

| Sex | Prevalence | Frequency | 1998 to 2005 | 2007 to 2009 | 2010 to 2012 | 2013 to 2015 | 2016 to 2019 | 2020 | 2021 |
| --- | --- | --- | --- | --- | --- | --- | --- | --- | --- |
| Men | Prevalence | Non-drinker | 28.62 (26.03 to 31.22) | 32.55 (29.63 to 35.47) | 31.04 (28.03 to 34.05) | 29.23 (26.74 to 31.72) | 35.99 (33.49 to 38.50) | 40.28 (34.99 to 45.57) | 32.94 (28.75 to 37.12) |
|  |  | 1 to 3 times a week | 28.00 (26.01 to 29.99) | 24.54 (23.09 to 25.99) | 26.96 (25.40 to 28.51) | 24.17 (22.75 to 25.60) | 26.44 (25.25 to 27.63) | 29.70 (27.15 to 32.25) | 27.09 (24.59 to 29.60) |
|  |  | ≥4 times a week | 41.90 (37.45 to 46.35) | 42.10 (38.59 to 45.62) | 47.99 (44.20 to 51.78) | 40.10 (36.28 to 43.93) | 49.57 (46.33 to 52.82) | 45.88 (38.58 to 53.18) | 45.88 (38.68 to 53.08) |
|  | Awareness | Non-drinker | 36.65 (32.16 to 41.14) | 64.72 (59.90 to 69.54) | 53.92 (48.67 to 59.18) | 57.21 (52.31 to 62.11) | 71.93 (67.93 to 75.93) | 72.07 (64.33 to 79.82) | 82.91 (76.74 to 89.09) |
|  |  | 1 to 3 times a week | 33.26 (29.41 to 37.11) | 47.39 (44.26 to 50.51) | 44.43 (41.36 to 47.49) | 52.29 (48.88 to 55.71) | 60.38 (57.95 to 62.81) | 63.04 (58.68 to 67.40) | 69.17 (63.75 to 74.58) |
|  |  | ≥4 times a week | 36.07 (30.11 to 42.03) | 45.65 (40.06 to 51.23) | 51.44 (46.06 to 56.83) | 57.65 (51.68 to 63.63) | 61.12 (56.88 to 65.36) | 61.26 (49.66 to 72.87) | 57.79 (48.37 to 67.20) |
|  | Treatment | Non-drinker | 32.27 (27.79 to 36.75) | 60.08 (55.10 to 65.06) | 50.16 (44.86 to 55.45) | 54.01 (49.05 to 58.97) | 69.01 (64.97 to 73.04) | 68.68 (61.19 to 76.17) | 81.24 (75.14 to 87.35) |
|  |  | 1 to 3 times a week | 27.00 (23.46 to 30.53) | 39.05 (36.05 to 42.06) | 40.11 (37.17 to 43.05) | 48.03 (44.69 to 51.37) | 56.13 (53.63 to 58.63) | 57.79 (53.11 to 62.48) | 64.24 (58.89 to 69.60) |
|  |  | ≥4 times a week | 30.30 (24.42 to 36.17) | 39.14 (33.61 to 44.67) | 46.43 (41.15 to 51.71) | 55.64 (49.72 to 61.56) | 57.61 (53.32 to 61.89) | 57.91 (46.77 to 69.06) | 54.67 (45.55 to 63.80) |
|  | Control | Non-drinker | 13.36 (9.94 to 16.79) | 39.35 (34.12 to 44.59) | 33.76 (28.88 to 38.65) | 39.56 (35.02 to 44.10) | 53.26 (49.04 to 57.47) | 51.83 (43.67 to 59.99) | 64.12 (56.23 to 72.00) |
|  |  | 1 to 3 times a week | 12.12 (9.30 to 14.95) | 24.81 (22.14 to 27.48) | 25.86 (23.46 to 28.25) | 34.74 (31.83 to 37.66) | 42.46 (39.99 to 44.94) | 43.88 (39.25 to 48.51) | 52.20 (46.65 to 57.75) |
|  |  | ≥4 times a week | 13.22 (8.73 to 17.70) | 19.34 (14.98 to 23.70) | 31.21 (26.60 to 35.82) | 37.65 (32.01 to 43.28) | 42.29 (38.18 to 46.41) | 35.46 (25.09 to 45.83) | 39.46 (30.69 to 48.22) |
|  | Control among treated | Non-drinker | 41.41 (33.22 to 49.60) | 65.50 (59.10 to 71.90) | 67.31 (60.84 to 73.79) | 73.25 (67.82 to 78.68) | 77.18 (73.13 to 81.22) | 75.47 (67.78 to 83.15) | 78.92 (72.18 to 85.65) |
|  |  | 1 to 3 times a week | 44.91 (37.03 to 52.79) | 63.54 (58.68 to 68.39) | 64.47 (60.65 to 68.28) | 72.34 (68.66 to 76.01) | 75.65 (73.06 to 78.25) | 75.93 (70.57 to 81.29) | 81.26 (76.62 to 85.90) |
|  |  | ≥4 times a week | 43.63 (32.42 to 54.84) | 49.41 (40.65 to 58.18) | 67.22 (60.26 to 74.18) | 67.67 (60.32 to 75.01) | 73.42 (68.60 to 78.24) | 61.23 (49.83 to 72.62) | 72.17 (59.53 to 84.81) |
|  | | | | | | | | | |
| Women | Prevalence | Non-drinker | 25.28 (23.58 to 26.98) | 33.77 (31.93 to 35.61) | 34.62 (32.74 to 36.50) | 31.18 (29.47 to 32.89) | 38.18 (36.47 to 39.89) | 37.11 (33.59 to 40.63) | 39.91 (36.71 to 43.11) |
|  |  | 1 to 3 times a week | 13.27 (11.57 to 14.98) | 16.00 (14.86 to 17.13) | 17.78 (16.53 to 19.03) | 16.10 (14.95 to 17.24) | 18.15 (17.10 to 19.19) | 18.23 (16.04 to 20.43) | 17.56 (15.23 to 19.89) |
|  |  | ≥4 times a week | 29.88 (20.93 to 38.82) | 22.51 (16.90 to 28.12) | 25.80 (19.47 to 32.14) | 24.08 (17.60 to 30.56) | 31.92 (25.95 to 37.88) | 23.69 (12.85 to 34.54) | 27.70 (16.93 to 38.48) |
|  | Awareness | Non-drinker | 55.60 (52.51 to 58.69) | 76.17 (73.62 to 78.71) | 76.81 (74.07 to 79.55) | 74.23 (71.49 to 76.98) | 80.12 (78.13 to 82.10) | 82.95 (79.56 to 86.35) | 82.30 (78.00 to 86.60) |
|  |  | 1 to 3 times a week | 41.62 (35.09 to 48.16) | 62.54 (59.02 to 66.05) | 65.18 (61.99 to 68.38) | 69.21 (66.00 to 72.42) | 69.99 (67.31 to 72.67) | 67.92 (63.00 to 72.85) | 73.78 (68.45 to 79.12) |
|  |  | ≥4 times a week | 38.58 (20.76 to 56.40) | 59.02 (44.91 to 73.13) | 56.33 (41.70 to 70.96) | 59.96 (44.83 to 75.09) | 54.59 (43.52 to 65.66) | 67.99 (45.72 to 90.26) | 62.86 (37.99 to 87.73) |
|  | Treatment | Non-drinker | 52.03 (48.76 to 55.30) | 72.47 (69.72 to 75.21) | 73.84 (71.05 to 76.63) | 72.32 (69.59 to 75.06) | 77.92 (75.80 to 80.03) | 80.65 (77.02 to 84.29) | 80.32 (75.59 to 85.05) |
|  |  | 1 to 3 times a week | 36.20 (29.92 to 42.48) | 56.27 (52.61 to 59.92) | 61.69 (58.44 to 64.95) | 67.75 (64.53 to 70.97) | 67.41 (64.72 to 70.10) | 64.95 (59.64 to 70.26) | 72.58 (67.15 to 78.00) |
|  |  | ≥4 times a week | 39.21 (22.10 to 56.32) | 54.75 (40.68 to 68.81) | 54.76 (39.97 to 69.55) | 59.96 (44.13 to 75.79) | 54.59 (43.76 to 65.42) | 67.99 (46.02 to 89.96) | 62.86 (38.36 to 87.35) |
|  | Control | Non-drinker | 25.41 (22.45 to 28.38) | 45.62 (42.50 to 48.75) | 46.72 (43.50 to 49.95) | 49.62 (46.54 to 52.70) | 52.53 (49.99 to 55.08) | 53.12 (47.82 to 58.42) | 59.18 (54.00 to 64.36) |
|  |  | 1 to 3 times a week | 15.10 (10.35 to 19.84) | 37.46 (33.71 to 41.22) | 37.90 (34.60 to 41.20) | 50.75 (47.36 to 54.14) | 49.20 (46.46 to 51.94) | 49.22 (44.20 to 54.23) | 61.33 (55.04 to 67.62) |
|  |  | ≥4 times a week | 14.88 (2.71 to 27.05) | 37.53 (23.47 to 51.59) | 32.94 (19.05 to 46.84) | 46.82 (32.46 to 61.18) | 38.28 (28.33 to 48.24) | 51.85 (25.68 to 78.03) | 42.90 (17.70 to 68.09) |
|  | Control among treated | Non-drinker | 48.84 (44.33 to 53.35) | 62.96 (59.52 to 66.39) | 63.28 (59.88 to 66.68) | 68.61 (65.33 to 71.89) | 67.42 (64.74 to 70.11) | 65.87 (60.64 to 71.09) | 73.68 (69.00 to 78.36) |
|  |  | 1 to 3 times a week | 41.70 (31.22 to 52.19) | 66.58 (62.09 to 71.07) | 61.43 (57.22 to 65.63) | 74.91 (71.47 to 78.36) | 72.99 (70.26 to 75.71) | 75.78 (70.09 to 81.47) | 84.51 (79.58 to 89.44) |
|  |  | ≥4 times a week | 37.95 (17.16 to 58.74) | 68.55 (51.10 to 86.00) | 60.16 (41.83 to 78.48) | 78.09 (65.09 to 91.09) | 70.13 (59.50 to 80.76) | 76.27 (49.70 to 100.00) | 68.24 (33.22 to 100.00) |

CI, confidence interval.

**Supplementary Table 9.** Trends in hypertension prevalence, awareness, treatment, control, and control among treated rates by smoke frequency, weighted % (95% CI)

| Sex | Prevalence | Frequency | 1998 to 2005 | 2007 to 2009 | 2010 to 2012 | 2013 to 2015 | 2016 to 2019 | 2020 | 2021 |
| --- | --- | --- | --- | --- | --- | --- | --- | --- | --- |
| Men | Prevalence | Non-smoker | 28.76 (25.80 to 31.72) | 25.53 (22.97 to 28.08) | 23.19 (20.68 to 25.70) | 21.43 (19.53 to 23.33) | 24.86 (22.90 to 26.81) | 25.30 (21.45 to 29.14) | 21.13 (17.20 to 25.06) |
|  |  | Ex-smoker | 38.02 (34.81 to 41.23) | 35.91 (33.64 to 38.18) | 39.73 (37.62 to 41.84) | 34.88 (32.73 to 37.03) | 37.30 (35.68 to 38.92) | 38.69 (35.43 to 41.95) | 37.75 (34.07 to 41.44) |
|  |  | smoker | 26.41 (24.41 to 28.41) | 23.21 (21.46 to 24.96) | 27.06 (25.16 to 28.96) | 24.52 (22.49 to 26.55) | 27.80 (26.15 to 29.45) | 32.57 (29.03 to 36.12) | 27.45 (23.64 to 31.25) |
|  | Awareness | Non-smoker | 31.62 (26.95 to 36.28) | 45.64 (40.20 to 51.07) | 33.94 (29.09 to 38.79) | 45.28 (40.50 to 50.06) | 56.82 (52.79 to 60.85) | 61.35 (53.11 to 69.58) | 63.54 (54.78 to 72.31) |
|  |  | Ex-smoker | 43.89 (39.03 to 48.74) | 58.68 (55.25 to 62.11) | 59.71 (56.05 to 63.37) | 65.94 (62.31 to 69.57) | 69.90 (67.29 to 72.51) | 72.09 (66.85 to 77.32) | 75.32 (70.57 to 80.08) |
|  |  | smoker | 29.74 (25.88 to 33.61) | 42.54 (38.45 to 46.62) | 40.47 (36.86 to 44.09) | 46.42 (41.97 to 50.88) | 56.58 (53.00 to 60.15) | 56.12 (49.34 to 62.91) | 65.47 (57.52 to 73.42) |
|  | Treatment | Non-smoker | 31.09 (26.42 to 35.77) | 39.84 (34.59 to 45.10) | 30.72 (26.06 to 35.38) | 41.42 (36.68 to 46.15) | 52.03 (47.92 to 56.14) | 56.95 (47.98 to 65.91) | 60.99 (52.39 to 69.60) |
|  |  | Ex-smoker | 21.58 (18.34 to 24.82) | 33.40 (29.47 to 37.34) | 35.24 (31.80 to 38.68) | 42.80 (38.39 to 47.21) | 52.11 (48.58 to 55.64) | 50.79 (44.31 to 57.27) | 60.11 (52.61 to 67.60) |
|  |  | smoker | 38.13 (33.21 to 43.04) | 52.27 (48.79 to 55.75) | 55.70 (51.99 to 59.41) | 62.34 (58.66 to 66.02) | 66.90 (64.15 to 69.66) | 67.90 (62.27 to 73.52) | 71.57 (66.56 to 76.58) |
|  | Control | Non-smoker | 12.03 (8.05 to 16.01) | 23.59 (19.00 to 28.19) | 18.97 (15.11 to 22.83) | 29.46 (25.35 to 33.58) | 37.28 (33.35 to 41.21) | 39.66 (32.42 to 46.89) | 50.04 (41.49 to 58.60) |
|  |  | Ex-smoker | 16.11 (12.49 to 19.74) | 32.55 (29.10 to 36.00) | 36.77 (33.44 to 40.11) | 44.59 (41.05 to 48.12) | 51.08 (48.29 to 53.87) | 50.88 (45.04 to 56.72) | 54.77 (49.58 to 59.96) |
|  |  | smoker | 10.51 (8.11 to 12.92) | 20.61 (17.54 to 23.67) | 23.41 (20.33 to 26.49) | 31.06 (27.22 to 34.90) | 40.03 (36.64 to 43.42) | 38.17 (31.74 to 44.59) | 50.83 (43.11 to 58.56) |
|  | Control among treated | Non-smoker | 38.70 (28.26 to 49.14) | 59.22 (50.98 to 67.46) | 61.75 (53.03 to 70.46) | 71.14 (64.30 to 77.98) | 71.65 (66.83 to 76.48) | 69.64 (61.05 to 78.23) | 82.05 (74.12 to 89.98) |
|  |  | Ex-smoker | 48.73 (40.07 to 57.39) | 61.69 (55.45 to 67.94) | 66.44 (61.05 to 71.82) | 72.57 (67.11 to 78.02) | 76.82 (73.24 to 80.40) | 75.15 (68.17 to 82.13) | 84.57 (77.45 to 91.70) |
|  |  | smoker | 42.26 (34.39 to 50.14) | 62.27 (57.45 to 67.09) | 66.02 (62.08 to 69.97) | 71.52 (67.84 to 75.20) | 76.35 (73.60 to 79.10) | 74.94 (69.30 to 80.57) | 76.52 (71.69 to 81.35) |
|  | | | | | | | | | |
| Women | Prevalence | Non-smoker | 21.31 (19.89 to 22.73) | 22.91 (21.79 to 24.03) | 25.04 (23.77 to 26.30) | 22.69 (21.58 to 23.79) | 26.04 (24.94 to 27.13) | 26.40 (24.09 to 28.70) | 27.40 (25.11 to 29.70) |
|  |  | Ex-smoker | 23.16 (16.57 to 29.75) | 19.12 (15.62 to 22.62) | 17.00 (13.53 to 20.47) | 15.43 (12.10 to 18.76) | 19.62 (16.81 to 22.43) | 15.31 (9.52 to 21.09) | 16.63 (11.26 to 22.00) |
|  |  | smoker | 33.80 (28.10 to 39.50) | 18.94 (15.43 to 22.44) | 16.34 (13.05 to 19.64) | 16.30 (12.60 to 20.00) | 17.50 (14.47 to 20.54) | 15.13 (8.90 to 21.35) | 18.62 (11.00 to 26.24) |
|  | Awareness | Non-smoker | 52.92 (49.90 to 55.94) | 69.95 (67.46 to 72.43) | 71.52 (69.23 to 73.80) | 72.18 (70.02 to 74.35) | 75.73 (74.01 to 77.45) | 76.37 (73.34 to 79.40) | 79.28 (75.76 to 82.79) |
|  |  | Ex-smoker | 53.41 (37.20 to 69.62) | 71.45 (63.13 to 79.78) | 69.43 (59.00 to 79.87) | 72.04 (62.12 to 81.96) | 61.53 (53.83 to 69.23) | 73.40 (52.90 to 93.90) | 82.80 (71.63 to 93.97) |
|  |  | smoker | 50.38 (39.70 to 61.07) | 63.84 (54.42 to 73.26) | 61.27 (50.39 to 72.15) | 59.63 (48.21 to 71.05) | 65.35 (56.04 to 74.67) | 61.95 (39.93 to 83.96) | 54.47 (37.26 to 71.68) |
|  | Treatment | Non-smoker | 48.61 (45.64 to 51.59) | 65.22 (62.64 to 67.80) | 68.32 (65.98 to 70.67) | 70.76 (68.61 to 72.91) | 73.53 (71.78 to 75.28) | 73.86 (70.59 to 77.12) | 77.63 (73.85 to 81.41) |
|  |  | Ex-smoker | 50.84 (41.13 to 60.56) | 58.65 (48.99 to 68.32) | 57.83 (47.04 to 68.62) | 53.83 (42.91 to 64.75) | 61.53 (51.90 to 71.15) | 61.95 (38.64 to 85.25) | 51.56 (34.89 to 68.23) |
|  |  | smoker | 55.36 (40.19 to 70.52) | 64.80 (55.68 to 73.92) | 66.82 (55.98 to 77.66) | 69.04 (59.05 to 79.04) | 58.44 (50.47 to 66.41) | 68.03 (47.41 to 88.65) | 82.80 (71.72 to 93.87) |
|  | Control | Non-smoker | 23.01 (20.40 to 25.62) | 41.93 (39.26 to 44.61) | 43.07 (40.52 to 45.63) | 50.14 (47.74 to 52.54) | 51.37 (49.44 to 53.30) | 51.35 (47.37 to 55.32) | 60.01 (55.69 to 64.34) |
|  |  | Ex-smoker | 25.37 (11.88 to 38.87) | 41.53 (32.19 to 50.87) | 36.70 (27.18 to 46.22) | 53.16 (41.89 to 64.43) | 38.95 (31.40 to 46.49) | 51.72 (30.39 to 73.05) | 61.23 (44.73 to 77.73) |
|  |  | smoker | 26.94 (17.55 to 36.33) | 39.78 (29.82 to 49.75) | 32.95 (23.96 to 41.94) | 45.15 (34.10 to 56.19) | 46.07 (36.41 to 55.72) | 49.80 (27.66 to 71.95) | 51.56 (34.72 to 68.39) |
|  | Control among treated | Non-smoker | 47.34 (43.08 to 51.60) | 64.29 (61.42 to 67.17) | 63.04 (60.14 to 65.95) | 70.86 (68.35 to 73.37) | 69.86 (67.86 to 71.85) | 69.52 (65.37 to 73.68) | 77.31 (73.59 to 81.04) |
|  |  | Ex-smoker | 52.98 (39.12 to 66.85) | 67.82 (56.51 to 79.14) | 56.98 (43.38 to 70.58) | 83.87 (73.94 to 93.80) | 74.88 (64.78 to 84.97) | 80.40 (60.29 to 100.00) | 52.98 (39.12 to 66.85) |
|  |  | smoker | 45.84 (27.33 to 64.35) | 64.09 (53.35 to 74.84) | 54.92 (42.81 to 67.03) | 77.00 (66.68 to 87.31) | 66.65 (57.99 to 75.30) | 76.02 (59.29 to 92.75) | 73.95 (58.30 to 89.61) |

CI, confidence interval.**Supplementary Table 10.** The weighted crude prevalence, awareness, treatment, control, control among treated before and during the COVID-19 pandemic, 2020-2021.

| Weighted % (95% CI) | | 2020 to 2021 |
| --- | --- | --- |
| Overall | Hypertension | 28.52 (27.24 to 29.80) |
|  | Awareness | 71.83 (70.00 to 73.67) |
|  | Treatment | 68.54 (66.61 to 70.48) |
|  | Control | 51.80 (49.77 to 53.83) |
|  | Control among treated | 75.57 (73.67 to 77.47) |
| **Sex** | | |
| Men | Hypertension | 31.51 (29.92 to 33.10) |
|  | Awareness | 67.49 (64.78 to 70.21) |
|  | Treatment | 63.20 (60.46 to 65.95) |
|  | Control | 48.58 (45.74 to 51.42) |
|  | Control among treated | 76.86 (74.08 to 79.64) |
| Women | Hypertension | 25.55 (24.05 to 27.05) |
|  | Awareness | 77.16 (74.85 to 79.47) |
|  | Treatment | 75.09 (72.63 to 77.56) |
|  | Control | 55.75 (52.87 to 58.63) |
|  | Control among treated | 74.24 (71.57 to 76.92) |
| **Region of residence** | | |
| Urban | Hypertension | 26.71 (25.36 to 28.05) |
|  | Awareness | 71.01 (68.84 to 73.18) |
|  | Treatment | 67.69 (65.37 to 70.00) |
|  | Control | 51.33 (48.97 to 53.70) |
|  | Control among treated | 75.84 (73.66 to 78.01) |
| Rural | Hypertension | 38.11 (34.47 to 41.75) |
|  | Awareness | 74.88 (71.65 to 78.11) |
|  | Treatment | 71.71 (68.52 to 74.90) |
|  | Control | 53.54 (49.50 to 57.57) |
|  | Control among treated | 74.65 (70.71 to 78.60) |
| **Age group** | | |
| 19 to 59 | Hypertension | 17.73 (16.65 to 18.81) |
|  | Awareness | 56.22 (53.17 to 59.26) |
|  | Treatment | 51.45 (48.30 to 54.60) |
|  | Control | 40.61 (37.45 to 43.77) |
|  | Control among treated | 78.94 (75.31 to 82.56) |
| ≥60 | Hypertension | 56.04 (54.20 to 57.87) |
|  | Awareness | 84.44 (82.75 to 86.13) |
|  | Treatment | 82.34 (80.61 to 84.08) |
|  | Control | 60.83 (58.41 to 63.25) |
|  | Control among treated | 73.88 (71.64 to 76.11) |
| **Educational background** | | |
| High school or lower | Hypertension | 35.65 (34.06 to 37.24) |
|  | Awareness | 76.39 (74.45 to 78.33) |
|  | Treatment | 73.63 (71.66 to 75.59) |
|  | Control | 55.30 (53.11 to 57.49) |
|  | Control among treated | 75.10 (73.02 to 77.18) |
| College or higher | Hypertension | 17.92 (16.50 to 19.35) |
|  | Awareness | 58.37 (54.48 to 62.26) |
|  | Treatment | 53.51 (49.38 to 57.64) |
|  | Control | 41.46 (37.35 to 45.57) |
|  | Control among treated | 77.49 (72.72 to 82.26) |
| **Income** | | |
| Lowest or second quartile | Hypertension | 38.06 (36.06 to 40.07) |
|  | Awareness | 78.58 (76.41 to 80.75) |
|  | Treatment | 75.83 (73.60 to 78.06) |
|  | Control | 55.71 (53.07 to 58.35) |
|  | Control among treated | 73.47 (70.82 to 76.12) |
| Third or highest quartile | Hypertension | 22.91 (21.64 to 24.19) |
|  | Awareness | 65.31 (62.60 to 68.03) |
|  | Treatment | 61.48 (58.62 to 64.34) |
|  | Control | 47.97 (44.99 to 50.96) |
|  | Control among treated | 78.03 (75.12 to 80.95) |

**Supplementary Table 11.** The weighted crude prevalence, awareness, treatment, control, control among treated in over 70 years old participants before and during the COVID-19 pandemic, 2020-2021.

|  | Pre-pandemic | | | | | During the pandemic | |
| --- | --- | --- | --- | --- | --- | --- | --- |
| The rate, % (95% CI) | 1998 to 2005 | 2007 to 2009 | 2010 to 2012 | 2013 to 2015 | 2016 to 2019 | 2020 | 2021 |
| Hypertension | 59.36 (55.88 to 62.83) | 61.90 (59.43 to 64.36) | 62.88 (60.83 to 64.94) | 59.95 (57.65 to 62.25) | 67.82 (66.21 to 69.43) | 65.22 (62.09 to 68.35) | 66.38 (63.02 to 69.74) |
| Awareness | 35.06 (31.56 to 38.57) | 62.42 (59.66 to 65.19) | 65.29 (62.55 to 68.04) | 69.40 (67.00 to 71.81) | 75.20 (73.38 to 77.02) | 74.70 (71.20 to 78.20) | 74.91 (71.24 to 78.58) |
| Treatment | 52.47 (47.97 to 56.96) | 71.34 (68.76 to 73.93) | 79.46 (77.09 to 81.83) | 79.77 (77.50 to 82.03) | 84.98 (83.43 to 86.52) | 84.56 (81.59 to 87.53) | 87.53 (84.68 to 90.38) |
| Control | 20.54 (16.88 to 24.20) | 44.23 (40.97 to 47.48) | 51.94 (48.88 to 54.99) | 56.09 (53.30 to 58.88) | 58.93 (56.63 to 61.22) | 56.65 (52.34 to 60.96) | 63.49 (59.29 to 67.69) |
| Control among treated | 39.15 (33.35 to 44.94) | 61.99 (58.19 to 65.79) | 65.36 (62.26 to 68.47) | 70.32 (67.35 to 73.28) | 69.34 (67.01 to 71.68) | 67.00 (62.74 to 71.26) | 72.53 (68.46 to 76.61) |

CI, confidence interval.

**Supplementary Table 12.** Weighed trend of hypertension prevalence, awareness, treatment, control, and control among treated in over 70 years old participants: Estimated β-coefficients and weighted odds ratios with 95% CIs

|  | Weighted trends in prevalence, awareness, treatment and control of hypertension | | | | Weighted odds of before and during the pandemic, OR | | |
| --- | --- | --- | --- | --- | --- | --- | --- |
|  | Pre-pandemic era (1998 to 2019), β (95% CI) | Pandemic era (2020 to 2021), β (95% CI) | Trend difference, β diff (95% CI) | 2020 versus 1998 to 2019 | | 2021 versus 2020 |  |
| Hypertension | **0.063 (0.038 to 0.088)** | -0.039 (-0.273 to 0.194) | -0.102 (-0.337 to 0.133) | 1.08 (0.93 to 1.24) | | 1.05 (0.86 to 1.29) |  |
| Awareness | **0.170 (0.143 to 0.197)** | 0.081 (-0.154 to 0.316) | -0.089 (-0.326 to 0.148) | **1.47 (1.21 to 1.78)** | | 1.01 (0.77 to 1.33) |  |
| Treatment | **0.148 (0.122 to 0.175)** | 0.185 (-0.013 to 0.383) | 0.037 (-0.163 to 0.237) | **1.48 (1.17 to 1.88)** | | 1.28 (0.91 to 1.81) |  |
| Control | **0.163 (0.128 to 0.198)** | **0.410 (0.102 to 0.718)** | 0.247 (-0.063 to 0.557) | 1.19 (0.99 to 1.43) | | **1.33 (1.03 to 1.71)** |  |
| Control among treated | **0.086 (0.046 to 0.126)** | **0.330 (0.016 to 0.645)** | 0.244 (-0.073 to 0.561) | 1.02 (0.83 to 1.25) | | 1.30 (0.98 to 1.73) |  |

CI, confidence interval.

Numbers in bold indicate a significant difference (*P* < 0.05).

**Supplementary Table 13.** National trend of the weighted and age-standardized prevalence rates before and during the COVID-19 pandemic, 1998-2021.

| Hypertension prevalence, weighted % (95% CI) | Pre-pandemic | | | | | During the pandemic | |
| --- | --- | --- | --- | --- | --- | --- | --- |
|  | 1998 to 2005 | 2007 to 2009 | 2010 to 2012 | 2013 to 2015 | 2016 to 2019 | 2020 | 2021 |
| Overall | 19.01 (17.96 to 20.06) | 19.87 (18.96 to 20.78) | 22.31 (21.38 to 23.24) | 20.42 (19.57 to 21.27) | 23.96 (23.19 to 24.73) | 25.96 (24.26 to 27.66) | 24.88 (23.12 to 26.63) |
| Men | 24.50 (22.88 to 26.12) | 22.95 (21.48 to 24.42) | 27.00 (25.59 to 28.42) | 24.09 (22.81 to 25.37) | 27.63 (26.57 to 28.69) | 31.07 (28.85 to 33.29) | 27.49 (25.19 to 29.79) |
| Women | 15.29 (14.16 to 16.42) | 15.59 (14.59 to 16.59) | 17.96 (16.95 to 18.98) | 17.02 (16.10 to 17.95) | 20.52 (19.59 to 21.44) | 21.08 (19.13 to 23.03) | 22.48 (20.45 to 24.52) |

CI, confidence interval.

**Supplementary Table 14.** Weighed and age-standardized trend of hypertension prevalence: Estimated β-coefficients and weighted odds ratios with 95% CIs

|  | Weighted trends in hypertension prevalence | | | Weighted odds of before and during the pandemic, OR | |
| --- | --- | --- | --- | --- | --- |
|  | Pre-pandemic era (1998 to 2019), β (95% CI) | Pandemic era (2020 to 2021), β (95% CI) | Trend difference, β_diff_ (95% CI) | 2020 versus 1998 to 2019 | 2021 versus 2020 |
| Overall | **0.138 (0.101 to 0.175)** | 0.092 (-0.014 to 0.199) | -0.046 (-0.158 to 0.067) | **1.23 (1.09 to 1.38)** | 1.09 (0.92 to 1.29) |
| Men | **0.107 (0.057 to 0.157)** | 0.043 (-0.079 to 0.165) | -0.064 (-0.196 to 0.068) | **1.30 (1.17 to 1.45)** | **0.84 (0.72 to 0.99)** |
| Women | **0.138 (0.101 to 0.175)** | 0.092 (-0.014 to 0.199) | -0.046 (-0.158 to 0.067) | **1.23 (1.09 to 1.38)** | 1.09 (0.92 to 1.29) |

CI, confidence interval.

Numbers in bold indicate a significant difference (*P* < 0.05).

**Supplementary Table 15.** Weighed trend of hypertension prevalence, awareness, treatment, control, and control among treated: Estimated β-coefficients and weighted odds ratios with 95% CIs

|  |  | Weighted trends in prevalence, awareness, treatment and control of hypertension | | | Weighted odds of before and during the pandemic, OR | |
| --- | --- | --- | --- | --- | --- | --- |
|  |  | Pre-pandemic era (2016 to 2019), β (95% CI) | Pandemic era (2019 to 2021), β (95% CI) | Trend difference, β diff (95% CI) | 2020 to 2016-2019 | 2021 to 2020 |
| Overall | Hypertension | 0.004 (-0.006 to 0.009) | 0.000 (-0.014 to 0.014) | 0.00 (-0.02 to 0.01) | 1.06 (0.96 to 1.17) | 0.96 (0.83 to 1.10) |
|  | Awareness | 0.017 (-0.005 to 0.019) | **0.036 (0.001 to 0.039)** | 0.01 (-0.01 to 0.04) | 1.07 (0.94 to 1.22) | **1.25 (1.04 to 1.51)** |
|  | Treatment | 0.005 (-0.008 to 0.017) | **0.024 (0.004 to 0.044)** | 0.02 (0.00 to 0.04) | 1.04 (0.91 to 1.19) | **1.29 (1.07 to 1.54)** |
|  | Control | 0.010 (-0.009 to 0.017) | **0.068 (0.021 to 0.062)** | **0.04 (0.01 to 0.06)** | 1.01 (0.89 to 1.15) | **1.41 (1.20 to 1.66)** |
|  | Control among treated | 0.002 (-0.011 to 0.015) | **0.034 (0.016 to 0.053)** | **0.03 (0.01 to 0.06)** | 0.97 (0.83 to 1.14) | **1.43 (1.17 to 1.74)** |
| **Sex, weighted % (95% CI)** | | | | | | |
| Men | Hypertension | -0.013 (-0.015 to 0.004) | 0.005 (-0.013 to 0.020) | 0.01 (-0.01 to 0.03) | **1.12 (1.00 to 1.26)** | 0.87 (0.75 to 1.01) |
|  | Awareness | 0.023 (-0.008 to 0.027) | 0.039 (-0.004 to 0.050) | 0.01 (-0.02 to 0.05) | 1.09 (0.91 to 1.31) | **1.29 (1.01 to 1.66)** |
|  | Treatment | 0.007 (-0.011 to 0.026) | 0.027 (-0.001 to 0.055) | 0.02 (-0.01 to 0.05) | 1.06 (0.88 to 1.27) | **1.31 (1.03 to 1.66)** |
|  | Control | 0.033 (-0.003 to 0.032) | **0.051 (0.004 to 0.060)** | 0.02 (-0.02 to 0.05) | 1.00 (0.84 to 1.19) | **1.40 (1.11 to 1.75)** |
|  | Control among treated | 0.015 (-0.004 to 0.034) | 0.018 (-0.008 to 0.045) | 0.00 (-0.03 to 0.04) | 0.92 (0.73 to 1.16) | **1.37 (1.01 to 1.86)** |
| Women | Hypertension | 0.021 (-0.001 to 0.017) | -0.006 (-0.019 to 0.013) | -0.01 (-0.03 to 0.01) | 0.99 (0.87 to 1.13) | 1.06 (0.90 to 1.26) |
|  | Awareness | 0.001 (-0.015 to 0.015) | 0.035 (-0.005 to 0.042) | 0.02 (-0.01 to 0.05) | 1.07 (0.88 to 1.29) | 1.16 (0.89 to 1.52) |
|  | Treatment | -0.002 (-0.018 to 0.013) | 0.024 (-0.002 to 0.049) | 0.03 (0.00 to 0.06) | 1.05 (0.87 to 1.26) | 1.21 (0.93 to 1.58) |
|  | Control | -0.021 (-0.026 to 0.007) | **0.088 (0.026 to 0.080)** | **0.06 (0.03 to 0.09)** | 1.03 (0.87 to 1.23) | **1.41 (1.12 to 1.77)** |
|  | Control among treated | -0.011 (-0.028 to 0.006) | **0.049 (0.023 to 0.075)** | **0.06 (0.03 to 0.09)** | 1.01 (0.82 to 1.25) | **1.49 (1.13 to 1.98)** |
| **Region of residence, weighted % (95% CI)** | | | | | | |
| Urban | Hypertension | -0.003 (-0.015 to 0.013) | 0.003 (-0.018 to 0.021) | 0.00 (-0.02 to 0.03) | 1.09 (0.98 to 1.21) | 0.87 (0.76 to 1.00) |
|  | Awareness | -0.024 (-0.037 to 0.016) | 0.020 (-0.036 to 0.059) | 0.02 (-0.03 to 0.08) | 1.12 (0.97 to 1.30) | 1.23 (1.00 to 1.53) |
|  | Treatment | -0.021 (-0.049 to 0.006) | 0.023 (-0.026 to 0.072) | 0.04 (-0.01 to 0.10) | 1.09 (0.94 to 1.27) | **1.29 (1.04 to 1.59)** |
|  | Control | -0.044 (-0.051 to 0.011) | 0.061 (-0.014 to 0.090) | 0.06 (0.00 to 0.12) | 1.05 (0.91 to 1.21) | **1.43 (1.19 to 1.72)** |
|  | Control among treated | -0.006 (-0.036 to 0.024) | 0.030 (-0.016 to 0.076) | 0.04 (-0.02 to 0.09) | 0.99 (0.82 to 1.18) | **1.46 (1.16 to 1.84)** |
| Rural | Hypertension | 0.001 (-0.030 to 0.031) | -0.006 (-0.042 to 0.035) | 0.00 (-0.05 to 0.05) | 0.93 (0.72 to 1.21) | **1.42 (1.04 to 1.93)** |
|  | Awareness | 0.022 (-0.040 to 0.058) | 0.065 (-0.025 to 0.094) | 0.03 (-0.05 to 0.10) | 0.86 (0.64 to 1.15) | 1.28 (0.89 to 1.83) |
|  | Treatment | 0.018 (-0.029 to 0.066) | 0.029 (-0.030 to 0.088) | 0.01 (-0.06 to 0.09) | 0.85 (0.64 to 1.13) | 1.21 (0.87 to 1.69) |
|  | Control | 0.039 (-0.028 to 0.064) | **0.112 (0.009 to 0.126)** | 0.05 (-0.02 to 0.12) | 0.86 (0.66 to 1.12) | 1.32 (0.95 to 1.82) |
|  | Control among treated | 0.006 (-0.039 to 0.051) | **0.060 (0.010 to 0.109)** | 0.05 (-0.01 to 0.12) | 0.91 (0.66 to 1.24) | 1.34 (0.88 to 2.03) |
| **Age group, weighted % (95% CI)** | | | | | | |
| 19 to 59 | Hypertension | -0.010 (-0.010 to 0.003) | -0.004 (-0.012 to 0.009) | 0.00 (-0.01 to 0.01) | 1.06 (0.95 to 1.19) | 0.86 (0.74 to 1.01) |
|  | Awareness | 0.017 (-0.012 to 0.027) | 0.051 (-0.001 to 0.064) | 0.02 (-0.01 to 0.06) | 1.07 (0.89 to 1.28) | **1.36 (1.05 to 1.75)** |
|  | Treatment | 0.002 (-0.017 to 0.022) | **0.040 (0.007 to 0.074)** | 0.04 (0.00 to 0.08) | 1.01 (0.83 to 1.21) | **1.48 (1.14 to 1.91)** |
|  | Control | 0.025 (-0.008 to 0.030) | **0.098 (0.027 to 0.092)** | **0.05 (0.01 to 0.09)** | 0.99 (0.81 to 1.21) | **1.76 (1.35 to 2.28)** |
|  | Control among treated | 0.019 (-0.005 to 0.044) | **0.055 (0.018 to 0.091)** | 0.04 (-0.01 to 0.08) | 0.96 (0.72 to 1.29) | **2.00 (1.28 to 3.13)** |
| ≥60 | Hypertension | 0.002 (-0.011 to 0.012) | -0.021 (-0.031 to 0.006) | -0.01 (-0.04 to 0.01) | 0.93 (0.83 to 1.05) | 1.01 (0.87 to 1.17) |
|  | Awareness | -0.016 (-0.016 to 0.006) | 0.011 (-0.011 to 0.020) | 0.01 (-0.01 to 0.03) | 1.02 (0.84 to 1.25) | 0.98 (0.76 to 1.27) |
|  | Treatment | -0.006 (-0.017 to 0.006) | 0.005 (-0.011 to 0.021) | 0.01 (-0.01 to 0.03) | 1.04 (0.86 to 1.26) | 0.95 (0.75 to 1.21) |
|  | Control | -0.024 (-0.026 to 0.005) | 0.039 (0.000 to 0.046) | **0.03 (0.01 to 0.06)** | 1.00 (0.85 to 1.18) | 1.13 (0.92 to 1.38) |
|  | Control among treated | -0.008 (-0.022 to 0.007) | **0.024 (0.002 to 0.046)** | **0.03 (0.00 to 0.06)** | 0.98 (0.81 to 1.18) | 1.23 (0.98 to 1.56) |
| **Educational background, weighted % (95% CI)** | | | | | | |
| High school or lower | Hypertension | **0.024 (0.000 to 0.020)** | 0.000 (-0.017 to 0.017) | -0.01 (-0.03 to 0.01) | 1.03 (0.92 to 1.16) | 1.03 (0.89 to 1.19) |
|  | Awareness | 0.005 (-0.010 to 0.015) | **0.041 (0.003 to 0.040)** | 0.02 (0.00 to 0.04) | 1.06 (0.90 to 1.25) | 1.24 (1.00 to 1.54) |
|  | Treatment | 0.001 (-0.012 to 0.014) | **0.024 (0.004 to 0.043)** | 0.02 (0.00 to 0.05) | 1.07 (0.91 to 1.25) | 1.22 (0.99 to 1.49) |
|  | Control | 0.000 (-0.014 to 0.014) | **0.069 (0.022 to 0.063)** | **0.04 (0.02 to 0.07)** | 1.06 (0.91 to 1.22) | **1.30 (1.09 to 1.56)** |
|  | Control among treated | -0.001 (-0.015 to 0.013) | **0.034 (0.015 to 0.054)** | **0.04 (0.01 to 0.06)** | 1.03 (0.86 to 1.23) | **1.31 (1.05 to 1.63)** |
| College or higher | Hypertension | -0.020 (-0.015 to 0.002) | -0.002 (-0.014 to 0.013) | 0.01 (-0.01 to 0.02) | 1.12 (0.97 to 1.30) | 0.85 (0.71 to 1.03) |
|  | Awareness | 0.040 (-0.008 to 0.043) | 0.025 (-0.026 to 0.056) | 0.00 (-0.05 to 0.05) | 1.13 (0.90 to 1.43) | 1.27 (0.92 to 1.76) |
|  | Treatment | 0.011 (-0.015 to 0.037) | 0.025 (-0.017 to 0.067) | 0.01 (-0.04 to 0.06) | 1.01 (0.79 to 1.30) | **1.45 (1.03 to 2.04)** |
|  | Control | 0.035 (-0.011 to 0.041) | 0.062 (-0.007 to 0.083) | 0.02 (-0.03 to 0.07) | 0.90 (0.70 to 1.16) | **1.78 (1.27 to 2.49)** |
|  | Control among treated | 0.013 (-0.017 to 0.043) | 0.034 (-0.012 to 0.080) | 0.02 (-0.03 to 0.08) | 0.76 (0.52 to 1.10) | **2.10 (1.22 to 3.61)** |
| **Income, weighted % (95% CI)** | | | | | | |
| Lowest or second quartile | Hypertension | 0.013 (-0.006 to 0.017) | 0.005 (-0.017 to 0.023) | 0.00 (-0.03 to 0.02) | 1.07 (0.94 to 1.23) | 1.01 (0.85 to 1.20) |
|  | Awareness | -0.021 (-0.023 to 0.006) | **0.063 (0.011 to 0.054)** | **0.04 (0.01 to 0.07)** | 1.09 (0.90 to 1.34) | 1.23 (0.94 to 1.60) |
|  | Treatment | -0.010 (-0.026 to 0.006) | **0.036 (0.013 to 0.059)** | **0.05 (0.02 to 0.07)** | 1.13 (0.93 to 1.37) | 1.18 (0.92 to 1.52) |
|  | Control | -0.029 (-0.029 to 0.004) | **0.084 (0.025 to 0.076)** | **0.06 (0.03 to 0.09)** | 1.03 (0.87 to 1.21) | **1.33 (1.08 to 1.65)** |
|  | Control among treated | -0.008 (-0.024 to 0.009) | **0.034 (0.008 to 0.059)** | **0.04 (0.01 to 0.07)** | 0.93 (0.76 to 1.15) | **1.39 (1.06 to 1.81)** |
| Third or highest quartile | Hypertension | -0.004 (-0.009 to 0.006) | 0.006 (-0.010 to 0.015) | 0.00 (-0.01 to 0.02) | 1.10 (0.98 to 1.23) | 0.92 (0.79 to 1.07) |
|  | Awareness | **0.050 (0.004 to 0.040)** | 0.018 (-0.018 to 0.040) | -0.01 (-0.05 to 0.02) | 1.11 (0.94 to 1.32) | 1.24 (0.97 to 1.58) |
|  | Treatment | **0.019 (0.000 to 0.037)** | 0.016 (-0.014 to 0.046) | 0.00 (-0.04 to 0.03) | 1.03 (0.86 to 1.23) | **1.34 (1.05 to 1.71)** |
|  | Control | **0.050 (0.004 to 0.040)** | **0.053 (0.002 to 0.064)** | 0.01 (-0.03 to 0.05) | 1.02 (0.86 to 1.21) | **1.46 (1.16 to 1.86)** |
|  | Control among treated | 0.014 (-0.005 to 0.034) | **0.033 (0.005 to 0.061)** | 0.02 (-0.02 to 0.05) | 1.00 (0.78 to 1.29) | **1.48 (1.06 to 2.06)** |

CI, confidence interval.

Numbers in bold indicate a significant difference (*P* < 0.05).

**Supplementary Table 16.** Ratio of ORs for association between the prevalence, awareness, treatment, and control of hypertension and each socioeconomic factor

| Variables | | Overall (2016 to 2021) | | Pre-COVID-19 pandemic (2016 to 2019) | | COVID-19 pandemic (2020 to 2021) | | Ratio of OR (95% CI) | p-value |
| --- | --- | --- | --- | --- | --- | --- | --- | --- | --- |
|  |  | Weighted OR (95% CI) | p-value | Weighted OR (95% CI) | p-value | Weighted OR (95% CI) | p-value |  |  |
| Hypertension | |  |  |  |  |  |  |  |  |
| Sex | Women | 1.00 (reference) |  | 1.00 (reference) |  | 1.00 (reference) |  | 1.00 (reference) |  |
|  | Men | **1.32 (1.26 to 1.39)** | **<.0001** | **1.31 (1.23 to 1.40)** | **<.0001** | **1.34 (1.23 to 1.46)** | **<.0001** | 1.02 (0.92 to 1.13) | 0.680 |
| Age | ≥60 | 1.00 (reference) |  | 1.00 (reference) |  | 1.00 (reference) |  | 1.00 (reference) |  |
|  | 19 to 59 | **0.16 (0.16 to 0.17)** | **<.0001** | **0.16 (0.15 to 0.17)** | **<.0001** | **0.17 (0.15 to 0.19)** | **<.0001** | 1.05 (0.93 to 1.18) | 0.415 |
| Region | Rural | 1.00 (reference) |  | 1.00 (reference) |  | 1.00 (reference) |  | 1.00 (reference) |  |
|  | Urban | **0.63 (0.57 to 0.70)** | **<.0001** | **0.66 (0.58 to 0.75)** | **<.0001** | **0.59 (0.50 to 0.70)** | **<.0001** | 0.90 (0.73 to 1.12) | 0.352 |
| Education | College or higher | 1.00 (reference) |  | 1.00 (reference) |  | 1.00 (reference) |  | 1.00 (reference) |  |
|  | High school or lower | **2.52 (2.36 to 2.69)** | **<.0001** | **2.51 (2.33 to 2.71)** | **<.0001** | **2.54 (2.26 to 2.85)** | **<.0001** | 1.01 (0.88 to 1.16) | 0.888 |
| Income | Third or highest quartile | 1.00 (reference) |  | 1.00 (reference) |  | 1.00 (reference) |  | 1.00 (reference) |  |
|  | Lowest or second quartile | **2.03 (1.92 to 2.15)** | **<.0001** | **2.02 (1.88 to 2.16)** | **<.0001** | **2.07 (1.87 to 2.29)** | **<.0001** | 1.02 (0.91 to 1.16) | 0.697 |
| Awareness | |  |  |  |  |  |  |  |  |
| Sex | Women | 1.00 (reference) |  | 1.00 (reference) |  | 1.00 (reference) |  | 1.00 (reference) |  |
|  | Men | **0.59 (0.53 to 0.65)** | **<.0001** | **0.57 (0.51 to 0.64)** | **<.0001** | **0.62 (0.51 to 0.74)** | **<.0001** | 1.08 (0.87 to 1.34) | 0.513 |
| Age | ≥60 | 1.00 (reference) |  | 1.00 (reference) |  | 1.00 (reference) |  | 1.00 (reference) |  |
|  | 19 to 59 | **0.21 (0.19 to 0.23)** | **<.0001** | **0.19 (0.17 to 0.22)** | **<.0001** | **0.24 (0.20 to 0.28)** | **<.0001** | 1.22 (0.99 to 1.51) | 0.066 |
| Region | Rural | 1.00 (reference) |  | 1.00 (reference) |  | 1.00 (reference) |  | 1.00 (reference) |  |
|  | Urban | **0.70 (0.62 to 0.80)** | **<.0001** | **0.65 (0.55 to 0.78)** | **<.0001** | 0.82 (0.67 to 1.01) | 0.0575 | 1.26 (0.96 to 1.64) | 0.092 |
| Education | College or higher | 1.00 (reference) |  | 1.00 (reference) |  | 1.00 (reference) |  | 1.00 (reference) |  |
|  | High school or lower | **2.42 (2.17 to 2.70)** | **<.0001** | **2.49 (2.18 to 2.84)** | **<.0001** | **2.31 (1.90 to 2.80)** | **<.0001** | 0.93 (0.73 to 1.17) | 0.527 |
| Income | Third or highest quartile | 1.00 (reference) |  | 1.00 (reference) |  | 1.00 (reference) |  | 1.00 (reference) |  |
|  | Lowest or second quartile | **1.97 (1.78 to 2.17)** | **<.0001** | **1.99 (1.77 to 2.24)** | **<.0001** | **1.95 (1.64 to 2.31)** | **<.0001** | 0.98 (0.79 to 1.20) | 0.830 |
| Treatment | |  |  |  |  |  |  |  |  |
| Sex | Women | 1.00 (reference) |  | 1.00 (reference) |  | 1.00 (reference) |  | 1.00 (reference) |  |
|  | Men | **0.56 (0.51 to 0.61)** | **<.0001** | **0.55 (0.49 to 0.61)** | **<.0001** | **0.57 (0.48 to 0.68)** | **<.0001** | 1.04 (0.84 to 1.29) | 0.701 |
| Age | ≥60 | 1.00 (reference) |  | 1.00 (reference) |  | 1.00 (reference) |  | 1.00 (reference) |  |
|  | 19 to 59 | **0.20 (0.18 to 0.22)** | **<.0001** | **0.19 (0.17 to 0.22)** | **<.0001** | **0.23 (0.19 to 0.27)** | **<.0001** | 1.19 (0.97 to 1.47) | 0.095 |
| Region | Rural | 1.00 (reference) |  | 1.00 (reference) |  | 1.00 (reference) |  | 1.00 (reference) |  |
|  | Urban | **0.69 (0.61 to 0.79)** | **<.0001** | **0.64 (0.53 to 0.76)** | **<.0001** | 0.83 (0.68 to 1.00) | 0.051 | **1.30 (1.00 to 1.68)** | **0.046** |
| Education | College or higher | 1.00 (reference) |  | 1.00 (reference) |  | 1.00 (reference) |  | 1.00 (reference) |  |
|  | High school or lower | **2.47 (2.22 to 2.75)** | **<.0001** | **2.50 (2.19 to 2.85)** | **<.0001** | **2.43 (2.01 to 2.93)** | **<.0001** | 0.97 (0.77 to 1.22) | 0.807 |
| Income | Third or highest quartile | 1.00 (reference) |  | 1.00 (reference) |  | 1.00 (reference) |  | 1.00 (reference) |  |
|  | Lowest or second quartile | **1.91 (1.73 to 2.10)** | **<.0001** | **1.90 (1.69 to 2.14)** | **<.0001** | **1.97 (1.67 to 2.32)** | **<.0001** | 1.03 (0.84 to 1.27) | 0.749 |
| Control | |  |  |  |  |  |  |  |  |
| Sex | Women | 1.00 (reference) |  | 1.00 (reference) |  | 1.00 (reference) |  | 1.00 (reference) |  |
|  | Men | **0.77 (0.71 to 0.84)** | **<.0001** | **0.78 (0.71 to 0.87)** | **<.0001** | **0.75 (0.64 to 0.88)** | **0.001** | 0.96 (0.79 to 1.16) | 0.662 |
| Age | ≥60 | 1.00 (reference) |  | 1.00 (reference) |  | 1.00 (reference) |  | 1.00 (reference) |  |
|  | 19 to 59 | **0.38 (0.35 to 0.42)** | **<.0001** | **0.36 (0.32 to 0.40)** | **<.0001** | **0.44 (0.37 to 0.52)** | **<.0001** | **1.23 (1.01 to 1.50)** | **0.041** |
| Region | Rural | 1.00 (reference) |  | 1.00 (reference) |  | 1.00 (reference) |  | 1.00 (reference) |  |
|  | Urban | **0.79 (0.70 to 0.90)** | **0.0002** | **0.73 (0.62 to 0.87)** | **0.0003** | 0.92 (0.76 to 1.11) | 0.361 | 1.25 (0.97 to 1.61) | 0.085 |
| Education | College or higher | 1.00 (reference) |  | 1.00 (reference) |  | 1.00 (reference) |  | 1.00 (reference) |  |
|  | High school or lower | **1.74 (1.57 to 1.94)** | **<.0001** | **1.75 (1.53 to 1.99)** | **<.0001** | **1.75 (1.45 to 2.10)** | **<.0001** | 1.00 (0.80 to 1.25) | 1.000 |
| Income | Third or highest quartile | 1.00 (reference) |  | 1.00 (reference) |  | 1.00 (reference) |  | 1.00 (reference) |  |
|  | Lowest or second quartile | **1.39 (1.28 to 1.52)** | **<.0001** | **1.42 (1.28 to 1.59)** | **<.0001** | **1.36 (1.17 to 1.60)** | **0.0001** | 0.96 (0.79 to 1.16) | 0.665 |
| Control among treated | |  |  |  |  |  |  |  |  |
| Sex | Women | 1.00 (reference) |  | 1.00 (reference) |  | 1.00 (reference) |  | 1.00 (reference) |  |
|  | Men | **1.27 (1.14 to 1.43)** | **0.0001** | **1.33 (1.17 to 1.53)** | **0.001** | 1.15 (0.93 to 1.43) | 0.192 | 0.86 (0.67 to 1.11) | 0.257 |
| Age | ≥60 | 1.00 (reference) |  | 1.00 (reference) |  | 1.00 (reference) |  | 1.00 (reference) |  |
|  | 19 to 59 | 1.15 (1.00 to 1.31) | 0.984 | 1.07 (0.91 to 1.27) | 0.424 | **1.33 (1.03 to 1.70)** | **0.028** | 1.23 (0.91 to 1.66) | 0.168 |
| Region | Rural | 1.00 (reference) |  | 1.00 (reference) |  | 1.00 (reference) |  | 1.00 (reference) |  |
|  | Urban | 0.99 (0.86 to 1.15) | 0.246 | 0.96 (0.80 to 1.16) | 0.354 | 1.07 (0.84 to 1.36) | 0.604 | 1.11 (0.82 to 1.51) | 0.496 |
| Education | College or higher | 1.00 (reference) |  | 1.00 (reference) |  | 1.00 (reference) |  | 1.00 (reference) |  |
|  | High school or lower | **0.83 (0.71 to 0.98)** | **0.037** | 0.81 (0.67 to 0.98) | 0.132 | 0.88 (0.65 to 1.18) | 0.383 | 1.08 (0.76 to 1.53) | 0.673 |
| Income | Third or highest quartile | 1.00 (reference) |  | 1.00 (reference) |  | 1.00 (reference) |  | 1.00 (reference) |  |
|  | Lowest or second quartile | **0.83 (0.74 to 0.94)** | **0.001** | **0.87 (0.75 to 1.00)** | **0.020** | **0.78 (0.62 to 0.98)** | **0.029** | 0.90 (0.69 to 1.18) | 0.449 |

CI, confidence interval.

Numbers in bold indicate a significant difference (*P* < 0.05).
